# Capturing technological crossovers between clay crafts: An archaeometric perspective on the emergence of workshop production in Late Iron Age northern Spain

**DOI:** 10.1371/journal.pone.0283343

**Published:** 2023-05-05

**Authors:** Beatrijs G. de Groot, Kamal Badreshany, Jesús F. Torres-Martínez, Manuel Fernández-Götz

**Affiliations:** 1 School of History, Classics and Archaeology, University of Edinburgh, Edinburgh, United Kingdom; 2 Department of Archaeology, Durham University, Durham, United Kingdom; 3 Instituto Monte Bernorio de Estudios de la Antigüedad del Cantábrico (IMBEAC), Madrid, Spain; German Archaeological Institute: Deutsches Archaologisches Institut, GERMANY

## Abstract

In the Iberian Iron Age, the transition to workshop-based pottery production involved the use of innovative tools (the potter’s wheel and kiln) and dedicated workspace. This facilitated an intensification of production, with repercussions for consumption practices and the economy. Cross-craft comparison can contribute to understanding the transmission processes underpinning this transition, as well as its impact on local craft traditions. This paper discusses an archaeometric methodology to compare the technological procedures underpinning different clay crafts to reveal crossovers and divergences that are meaningful for understanding cross craft interaction and the spread of technological innovations. We use thin-section ceramic petrography, X-Ray Fluorescence, Inductively Coupled Plasma–Mass Spectrometry, and X-Ray Diffraction to analyse the mineralogical and geochemical compositions and levels of standardisation in hand-made pottery, wheel-made ceramics, and ceramic building materials from the Late Iron Age *oppidum* of Monte Bernorio (Aguilar de Campoo, Palencia) and the kiln site of El Cerrito (Cella, Teruel). The results demonstrate that wheel-made pottery was produced according to a highly uniform clay preparation and clay selection procedure, which spanned the northern Iberian Plateau and largely existed in isolation from local pottery traditions. At Monte Bernorio, wheel-made pottery was made on-site from non-local clays, suggesting that suitable clays were brought to the site, perhaps by itinerant potters working on a seasonal basis. Technological traditions were thus largely polarised, demonstrating that knowledge, skills, and markets relating to workshop-produced pottery were enacted by a segment of society operating as part of a closed technological system.

## Introduction

Cross-craft comparison provides a framework for capturing technological crossovers between distinct craft traditions, which may reflect the social interactions underpinning innovation processes [1–4]. Raw material selection and provenance are particularly important indicators of social interaction, because such processes tend to conform to social rules and conventions instead of relating to distance and the convenience of procurement exclusively [5–8]. Comparing the raw materials utilised in the production of different classes of objects can therefore provide significant insights into the transmission of environmental and technological knowledge between craft practitioners.

One area of study to which cross-craft comparison can provide novel perspectives is the rise of specialised, workshop-based ceramic industries. The emergence of pottery workshops, marked by dedicated architecture and the use of innovations such as the potter’s wheel and double-chambered kiln, has been correlated to processes of economic differentiation and social hierarchisation [9, 10]. Workshop production implies a level of craft specialisation, which tends to occur during processes of intensification of production, in relation to a growing need for specific consumer goods [11–16]. In the Iberian Peninsula, the spread of the potter’s workshop is an asymmetrical process, with regional variation in the contexts and speed of its introduction [17–20]. Pottery workshops first appear on the southern Iberian coastline, in the context of Phoenician trading colonies, which were established during the 9^th^ and 8^th^ centuries BCE [17, 20, 21]. In such contexts, ceramic production forms part of an economic strategy based on the trade of foodstuffs in custom-made amphorae and luxury tableware. In the centuries that followed, pottery workshops appeared inland, becoming particularly prevalent in central and eastern Iberia during the second half of the first millennium BCE [19, 22]. The amphorae and luxury tablewares such workshops produced have been related to the emergence of novel consumption practices and trade networks. In eastern Iberia, such practices centre on the production and consumption of wine, popularising drinking cups of eastern Mediterranean style and wheel-made amphorae for storage and trade [23–25]. First modelled after Phoenician and Greek examples, Late Iron Age wheel-made pottery took its own form in central and eastern Iberia, exemplified by decorative ‘Celtiberian’ style ceramics [26, 27].

**Fig 1:**
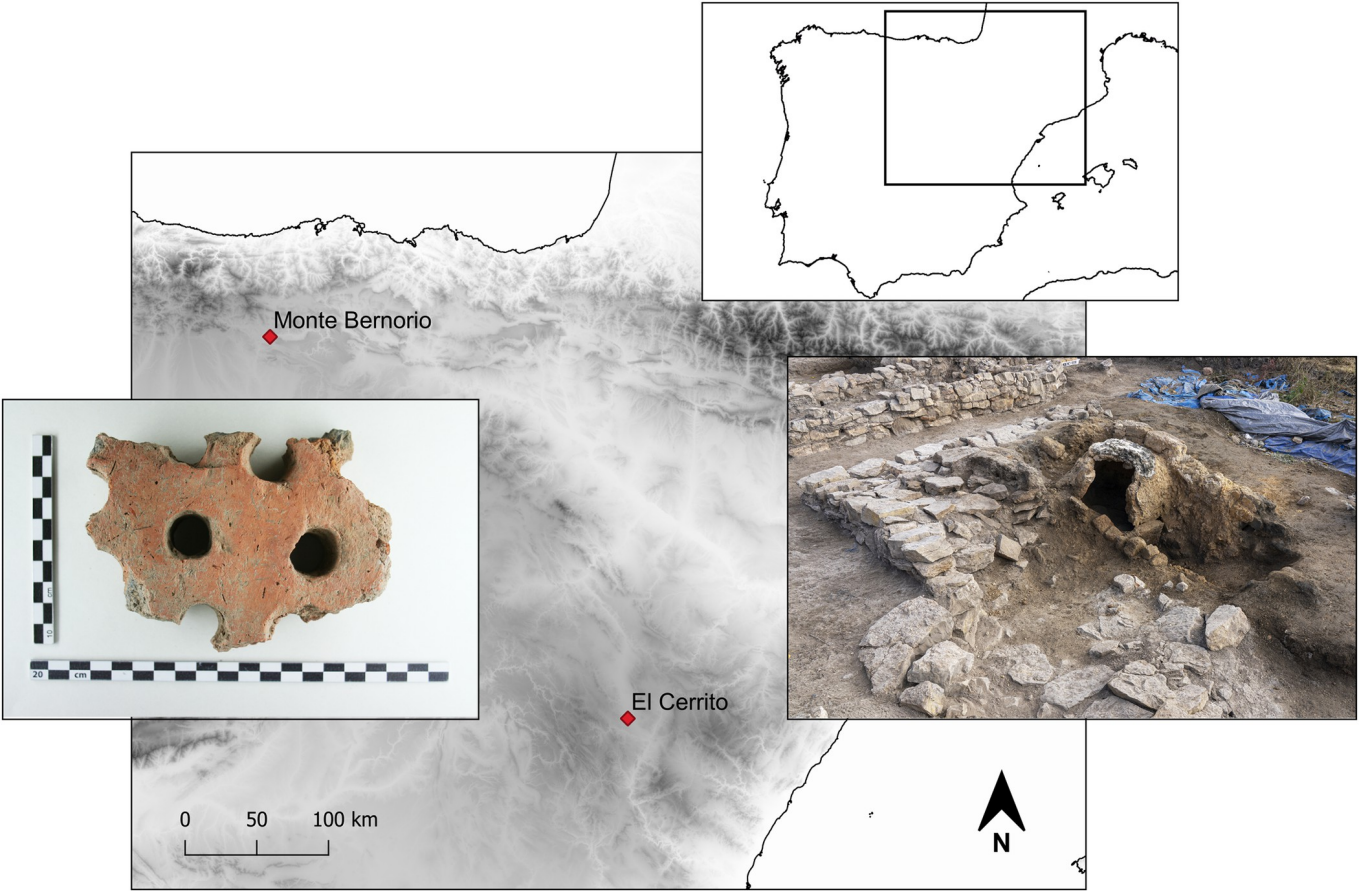
Locations of Monte Bernorio and El Cerrito. Left: piece of a grill from a double chambered kiln excavated from trench 3 at Monte Bernorio in 2007 (photography by J.F. Torres-Martínez, IMBEAC). Right: Ceramic kiln and combustion chamber at El Cerrito (photograph by A. Seisdedos, IMBEAC Cerrito team) (relief map is a hillshade based on Digital Elevation Models from Copernicus: https://doi.org/10.5270/ESA-c5d3d65).

Little is known about the way in which technological skills and knowledge were developed and transmitted, and what the role of ‘traditional’ potters may have been in the development of workshop-based ceramic production. It has been implied that the spread of workshop industries in the first millennium BCE Iberian Peninsula marks a discontinuous process, in which hand-made ceramics were produced in isolation from mass produced wheel-made pottery [22]. The continued production of hand-made pottery up until the Roman period suggests that distinct communities of practice developed and persisted alongside each other. Similar processes of polarisation have been observed in different craft contexts, indicating that technological innovations, though sometimes perceived as more efficient, do not spread automatically [6, 28, 29]. The present study utilises archaeometric methods to compare craft sequences, or *chaînes opératoires*, of hand-made and wheel-made ceramics from the northern part of the Central Iberian System, focusing on the Late Iron Age kiln site of El Cerrito (Cella, Teruel) and the *oppidum* of Monte Bernorio (Aguilar de Campoo, Palencia) (Fig 1). These sites were selected because both were excavated recently and date to the same time-period of 400–200 BCE, a period of adoption and consolidation of wheel-made Celtiberian-style pottery across the Central Iberian Plateau. The sites are located at the northern and eastern extent–respectively–of the distribution of such Celtiberian-style pottery, and therefore offer opportunities to explore variation in technological characteristics underpinning the production of these wares. Reflecting different archaeological contexts–an early urban centre with a large population in the case of Monte Bernorio and a small settlement with El Cerrito–these sites represent two ends of the spectrum of the type of settlements producing wheel-made ceramics during the same time-period.

Archaeometric and quantitative methods are combined to consider the characteristics of clay extraction and preparation within the workshop contexts, and if some of these procedures could have been inspired by different technological traditions, represented by hand-made pottery of local typology. Thus, by focusing on the provenance and preparation of clay and temper, this study considers how novel ceramic technologies (the potter’s wheel, double-chambered updraught kiln) were embedded into, and reshaped, the cultural landscapes of Late Iron Age communities in the Iberian Peninsula.

The scientific study of archaeological materials enables characterising the clay mixtures of archaeological ceramics, as well as providing insights into other technological steps in the ceramic production process [30]. As such, archaeometric methods can provide empirical evidence of varied techno-economic strategies relating to clay procurement and preparation, thus also supporting the investigation of the technological choices that underpinned the production of novel ceramics produced in Late Iron Age pottery workshops. Despite the emergence of a body of archaeometric work on Iron Age ceramics in the western Mediterranean [31, e.g. 32–42], the dynamic between hand-made and wheel-made potting technologies and clay extraction for building materials has not yet been explored in this context prior to the present study. In response, this paper assesses whether traditional knowledge could have underpinned novel clay extraction habits associated with the potter’s wheel and how this knowledge was shared across technological boundaries. This study also examines and characterises the raw materials used in the construction of buildings and kiln architecture as a way to assess the exploitation strategies of clay materials more broadly. Finally, the paper addresses the association between Celtiberian-style pottery and fabric standardisation [43, 44] in order to more fully understand the role of the pottery workshop in trajectories towards mass production in Late Iron Age northern Spain.

Focusing on ceramic clay and temper is important for two reasons:

- Clay extraction and clay recipes are characterised as ‘insensitive’ to innovation through forming part of culturally transmitted practices and traditions [5, 7, 8] or ‘technological styles’ [45].
- Wheel-made pottery tends to contain smaller and less angular inclusions so as to avoid tearing marks on the surface and to protect potters’ hands [10, 46].

These observations suggest a conflict; if potters choose to shift to workshop production we can expect continuity in the selection of clay. This has been observed at sites like Setefilla (southern Spain) where some fabrics were used for the production of both hand-made and wheel-made pottery [47]. On the other hand, the nature of, particularly, the wheel-*throwing* process is such that it requires suitable clay recipes, allowing for the retention of the structural integrity of the clay during throwing as well as a suitable size and shape of inclusions so as to avoid damaging the vessel surface and potter’s hands. Thus, the potter’s wheel might introduce conflicting priorities, potentially leading to the abandonment of clay sources or temper recipes despite the traditional conformity to such technological choices.

### Capturing polarised ceramic crafts in Late Iron Age Iberia

Technological clustering, or ‘polarisation’, can be expected when technological standards of social groups conform to social boundaries, obstructing the diffusion of techniques between such groups [29]. Examining the diffusion of technological practices at the onset of workshop-based pottery production thus allows for investigating questions surrounding the social composition of a society, being composed of separate ‘polarised’ socio-technical groupings or reflecting a more homogeneous society in which technical skills are shared and adopted freely.

Archaeological evidence for the first pottery workshops derives from excavations of ceramic kiln sites, the earliest of which date to the beginning of the 6^th^ century BCE [19]. Workshops have also been recognised by proxy, through the identification of locally produced wheel-made ceramics of Phoenician, Greek, or Celtiberian typology. Archaeometric evidence demonstrates that such ceramics were produced locally as early and the 9^th^ and 8^th^ centuries BCE [39], though no kiln architecture accompanying these findings has been discovered to date. Hand-made ceramics of local/non-Phoenician or Greek typology were probably generally produced in household contexts and fired in bonfires or pit-kilns, suggesting that such ceramics were produced in separate context from wheel-made ceramics. However, both hand-made and wheel-made grey-ware ceramics were produced by Phoenician workshops, demonstrating the fluidity of production methods employed in such contexts [39, 48].

Furthermore, little research has gone into the production organisation and technology of Iron Age hand-made ceramics from Central Iberia, and there is some evidence for the specialisation and centralisation of production of such ceramics (i.e. Cogotas type pottery) prior to the introduction of Celtiberian workshops proper [49]. It is thus likely that the organisation underpinning the production and distribution of Iron Age hand-made pottery is more complex than often assumed. The present study contributes to this discussion through systematically comparing technological characteristics of hand-made and wheel-made pottery from the selected contexts.

### El Cerrito (Cella, Teruel)

**Fig 2:**
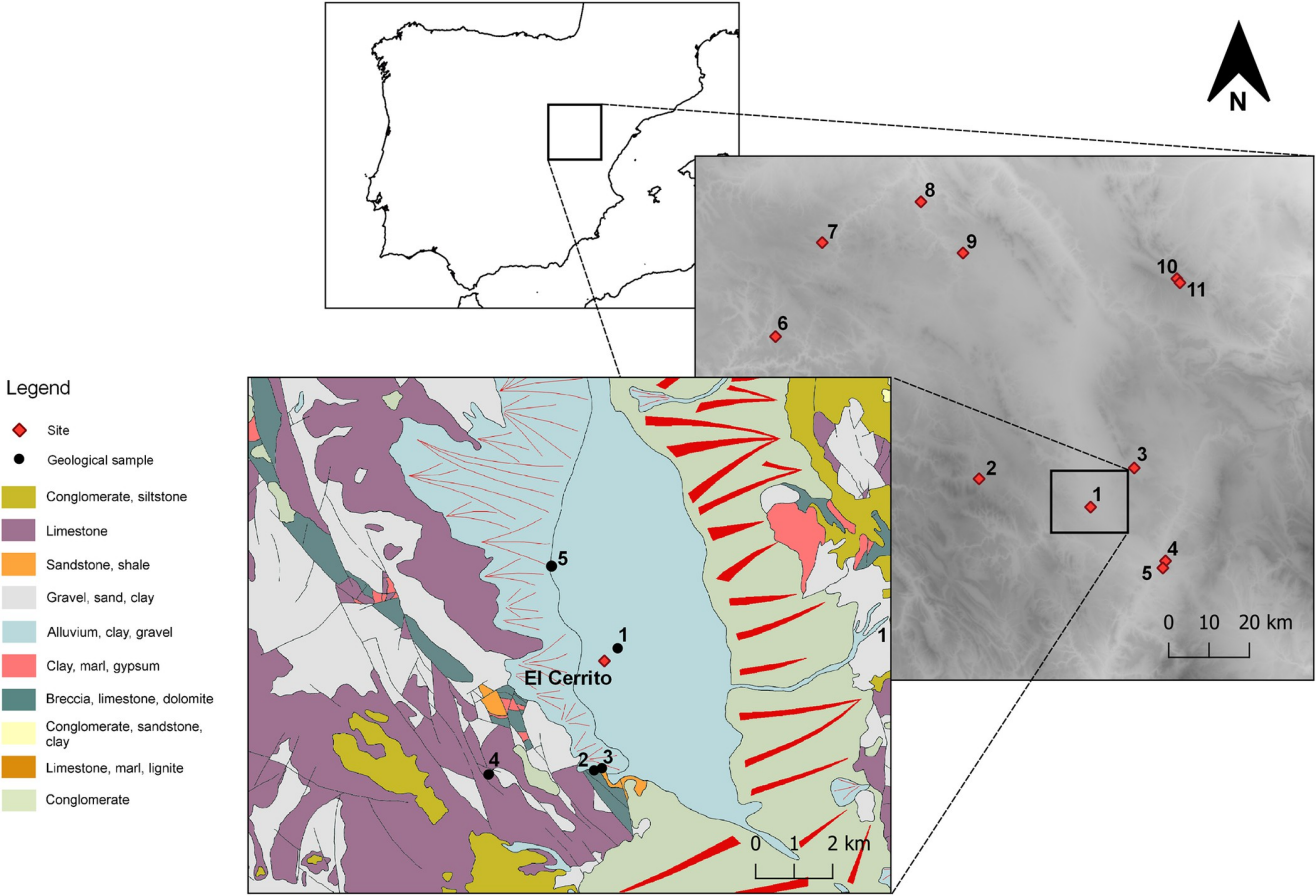
Geological map of El Cerrito with locations of geological samples, and contemporary sites included in the study. 1) El Cerrito, 2) Las Tejadas, 3) Las Veguillas, 4) Lavadero-Escobares, 5) Los Vicarios, 6) Hortezuelas III, 7) Modojos II, 8) Mojón de Ibdes II, 9) Barranco de la Cañada, 10) Cerra la Viña, 11) Allueva II (relief map is a hillshade based Digital Elevation Models from Copernicus: https://doi.org/10.5270/ESA-c5d3d65. Geological data is copied and simplified from the National Centre Geological and Mining Institute of Spain [51]).

El Cerrito is a small hillfort near the present-day town of Cella (Teruel), located within the lagoon of El Cañizar, which is a humid depression located at 989 m above sea level on the Cordillera Ibérica in the ‘Fosa del Jiloca’ basin [50]. This basin, shaped through Upper Pliocene and Quaternary glaciation, is determined by north-west, south-east running fault lines. The lagoon is situated on Quaternary lacustrine deposits affected by large alluvial fans depositing heterogeneous materials from the Sistema Ibérico. These materials include Mesozoic limestone and Jurassic dolomite, Ordovician and Silurian slate, sandstone, greywacke, and quartzite, as well as Permo-Triassic shale, sandstone, breccia, conglomerate, dolomite, marl, and limonite [50, 53] (Fig 2).

Excavations at El Cerrito started in 2012, revealing the remains of a small fortified settlement surrounded by a rampart that enclosed an area of ca. 2,800 m^2^. Some of the houses were built into the rampart, following a pattern common in the east of the Iberian Peninsula. The surveys and excavations undertaken in recent years have shown that the hillfort had an area for houses, a ritual space, and workshops. Within the area of the workshops there is evidence for textile production as well as a potter’s kiln, next to which the remains of ceramics that had broken during the production process and ashes from the kiln have been identified. The kiln is located within a hollow inside the hillfort, using the slope in order to have the combustion chamber on the lower level and the kiln on the upper one. The dome of the kiln was surrounded by a mudbrick wall, whereas the combustion chamber was semi-subterranean and covered by mudbricks forming a tunnel. The entire structure was surrounded by stone walls on three of its sides and mudbricks on the other, separating the kiln from an area identified as a textile workshop. Overall, the kiln was small and used primarily for the production of small and medium size ceramics. The existence of additional kilns cannot be excluded in the areas of the hillfort that remain unexcavated.

Fieldwork at El Cerrito has produced a broad range of ceramic finds, including loom weights, fired clay, large hand-made and wheel-made storage vessels, and wheel-made fine ware [52]. Larger storage vessels may have been constructed using coils and finished on the wheel, as has been suggested for similar vessels from the contemporary potters workshop at Mas de Moreno (Foz-Calanda, Teruel) [53]. Hand-made, slab-, and coil-built pottery appeared around the kiln of El Cerrito (average wall-thickness of c 1.5 cm) and several large sherds were embedded in the dome, possibly placed there to contribute to its structural integrity and thermal shock resistance. Smaller vessels were probably largely wheel-thrown, although macro-traces of coils could be discerned in some of the samples studied in this paper (Fig 4). The wheel-made pottery often contains distinctive Celtiberian decorations: black, red and brown painted bands, spirals, and dashes. Such pottery is thin-walled (c. 0.5 cm) and largely fired in oxidising atmospheres. Dark-coloured, reduced fired, hand-made coarse-ware pottery was also found, suggesting such pottery was used for cooking rather than display or transport, and that such ceramics were fired in a bonfire or in the anoxic conditions of a pit kiln.

It is understood that kiln-sites like El Cerrito contributed to the intensified production of new types of storage and table-ware, emerging in unison with the increase of agricultural production and regional and long-distance trade [22, 54]. Standardisation among El Cerrito and neighbouring sites is reflected by the layout of the kilns excavated and by the typology and fabrics of their ceramics [55–57]. Archaeometric studies point out that wheel-made pottery in the region is made from illite-muscovite-rich clays with varied amounts of carbonates and ferruginous inclusions [56]. Existing studies focus exclusively on wheel-made pottery, and therefore we cannot confirm whether the production of hand-made pottery was common at such sites. The presence of hand-made coarse-ware pottery embedded in the kiln at El Cerrito suggests that such ceramics were produced on-site, but the archaeometric study discussed below will consider this interpretation further.

### Monte Bernorio (Aguilar de Campoo, Palencia)

**Fig 3:**
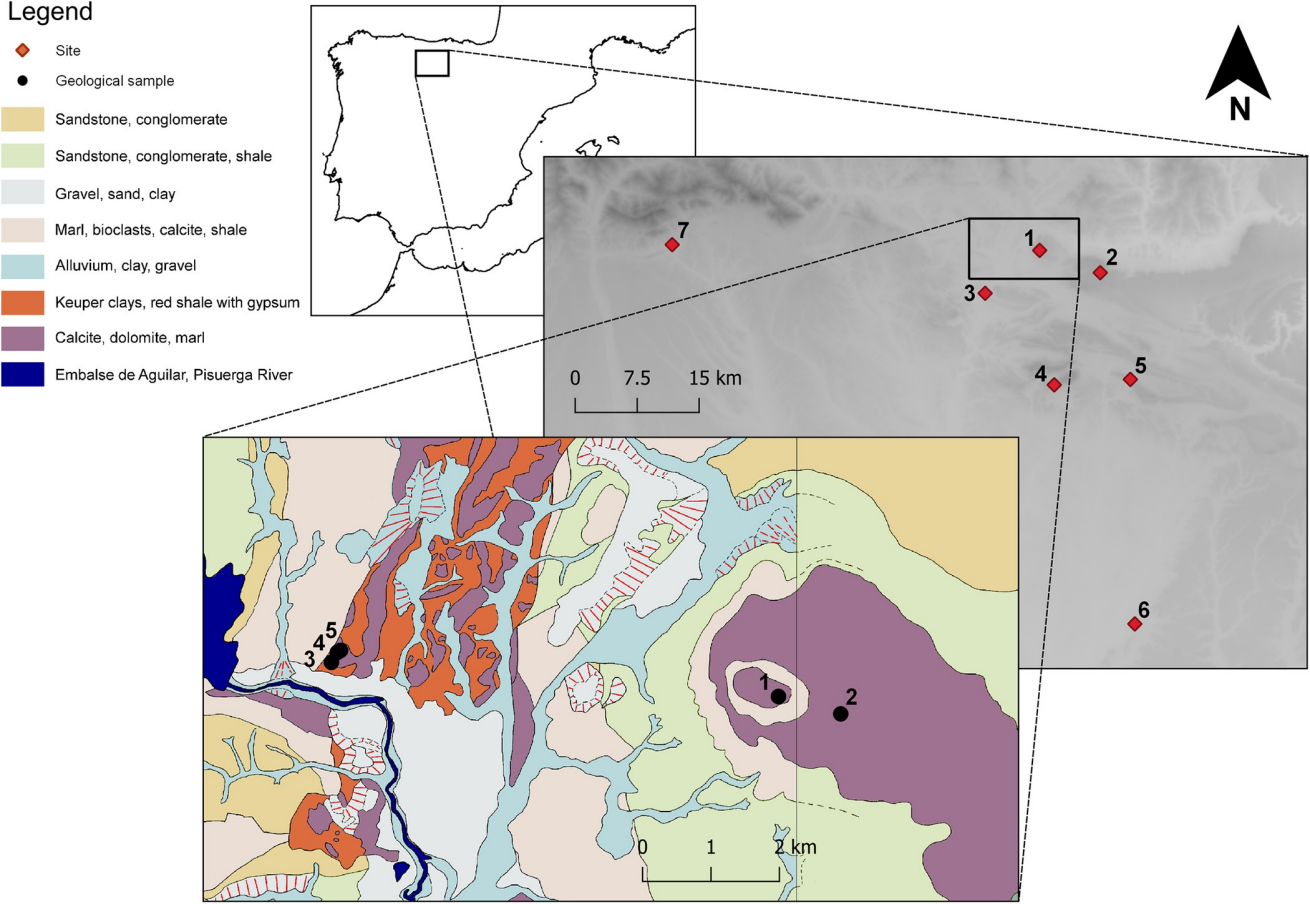
Geological map of Monte Bernorio with locations of geological samples and contemporary surrounding sites. 1) Monte Bernorio, 2) Las Loras, 3) Monte Cildá, 4) Peña Amaya, 5) La Ulaña, 6) Castarreño, 7) La Loma (relief map is a hillshade based Digital Elevation Models from Copernicus: https://doi.org/10.5270/ESA-c5d3d65. Geological data is copied and simplified from the National Centre Geological and Mining Institute of Spain [51]).

Monte Bernorio is situated at the intersection of the Central Iberian Plateau and the Cantabrian Mountain Range (Fig 3). Its geologically dynamic surroundings are part of the ‘plataforma burgalesa’ domain, characterised by an ESE-dipping monocline (a step-like fold in rock strata) bounded to the south by the right-lateral Ubierna fault system and by the Sierra Cantabria Thrust to the north [58: 523, 59]. The region is characterised by Triassic Keuper facies, which are formed by evaporites, anhydrites, and Lower Jurassic dolomites. These facies form a belt of red clays west of the *oppidum*. Monte Bernorio itself is a massif of Upper Cretaceous carbonates and lacustrine sediments surrounded by a base of marine Lower Cretaceous and Jurassic rock. The geological stratigraphy of the site can thus be divided into three phases, starting with a base of sandstone deposited through fluvial activity around 95 Mya, a middle section composed of marine calcareous sediment containing bivalves, deposited around 88–90 Mya, and a top section composed of calcareous rocks and marls deposited around 88–80 Mya [60: 336].

During the late 1st millennium BCE, Monte Bernorio was one of the largest fortified settlements (*oppida*; singular *oppidum*) on the Iberian Peninsula. While the site was already frequented in the Bronze Age, occupation intensified in the final stages of the Iron Age. In the 1st century BCE, the upper part of the mountain was surrounded by a wall and ditch system that enclosed an area of 28 ha. In addition, a series of outer linear earthworks (‘multivallate’) on the slopes and at the foot of the site extended the enclosed area to a total of c. 90 ha. Excavations within the site, while still limited in their extension, have revealed some house structures and an extraordinarily high amount of archaeological finds, including large quantities of pottery and animal bones, as well as some metalwork and other objects such as glass beads [61, 62]. In addition to the settlement information, several cremation cemeteries are known and have been partly excavated, revealing complex ritual practices in which fragmentation played a key role [63]. The end of the *oppidum* was the result of the Roman conquest by the troops of Emperor Augustus, probably around 26/25 BCE as part of the so-called Cantabrian and Asturian Wars (*Bellum Cantabricum et Asturicum*). There is ample evidence of destruction on the site, as well as considerable numbers of Roman military finds which, together with the information from a large Roman camp located in front of the *oppidum*, testify to the brutal end of the Iron Age occupation [64].

Monte Bernorio represents one of the most northerly production locations of Celtiberian-style pottery in the Iberian Peninsula. Wheel-made pottery, decorated with painted spirals, lines, and grids, reminiscent of the pottery styles of the Duero and Ebro valleys, has been found in abundance across the site [61]. There is a wide range of hand-build pottery types, some of which are made of fine clays and contain incised and applique decorations. Macroscopic analysis suggests that such ceramics are commonly tempered with crushed calcite, a technological choice common for the production of hand-made pottery in Cantabria [65] and Galicia [66]. Shapes include globular and s-shaped bowls, as well as tripods, all common forms for the Cantabrian region [67]. Macroscopic analysis suggests that hand-made pottery was usually coil- or slab built. Some vessels appear to have been finished and shaped utilising a slow wheel, as is common in the production of ceramics at the Late Bronze Age site of Las Cogotas [49]. Such ceramics are comparable to other hand-made vessels in the fabrics and firing treatments employed but also display traces of wheel-use. Similarly, some vessels formed using coiling or slab building were fired in oxidising conditions, suggesting these might have been fired in the double-chambered kilns normally used for the production of the Celtiberian pottery [67]. This evidence counters the categorical separation between hand-made and wheel-made pottery, pointing instead to the permeability of the production processes associated with either shaping method.

## Materials and methods

**Fig 4:**
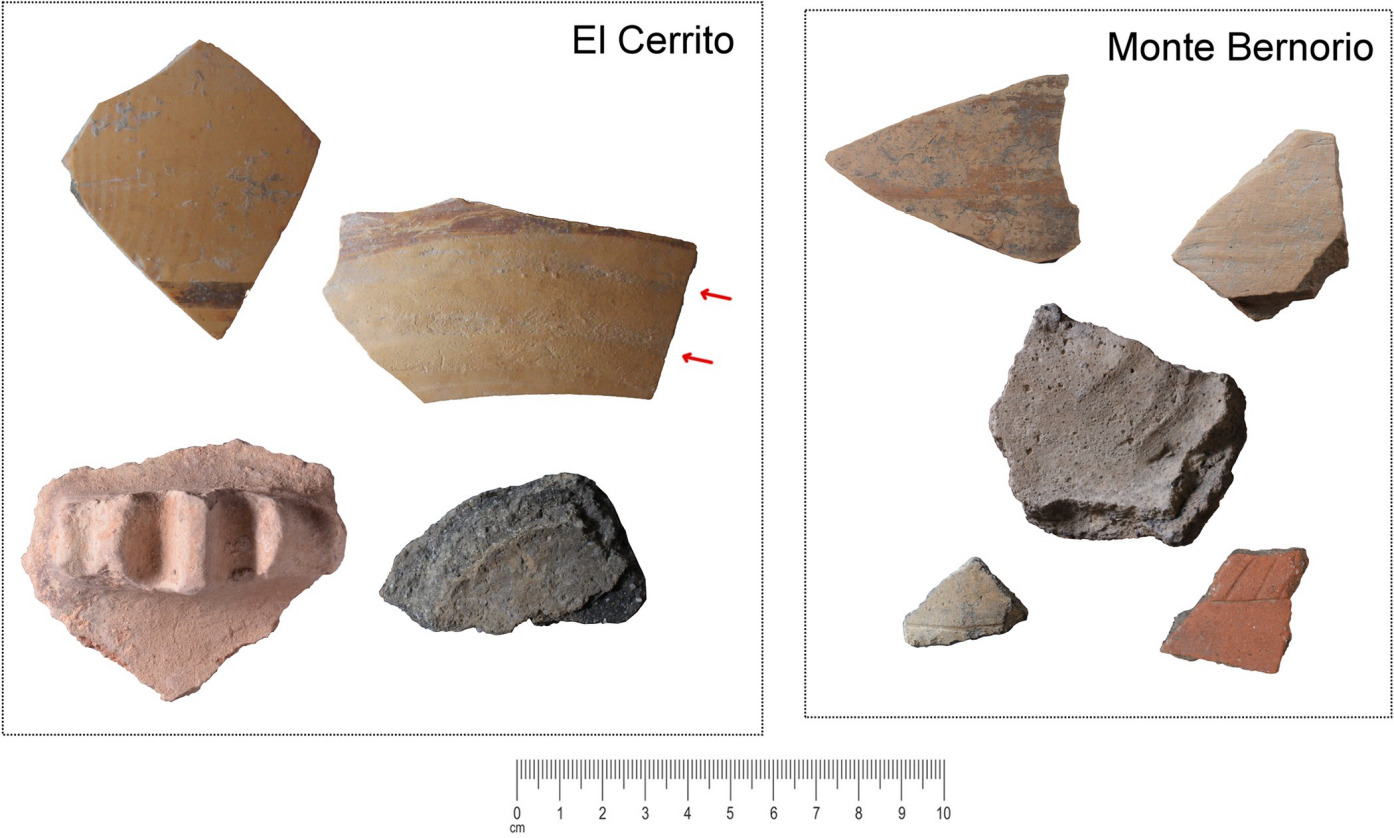
Examples of pottery samples from Monte Bernorio and El Cerrito. The red arrows indicate the location of coil joints suggesting wheel-coiling was used at El Cerrito.

In order to capture technological crossovers between hand-made and wheel-made pottery, as well as architectural clays, we utilise a methodology to reveal the mineralogical, compositional, textural, and microstructural composition of clay artefacts. Evidence for these different strands of information aids cross-comparison through providing quantitative or qualitative information (Table 1). A total of 91 ceramic fragments, 20 building materials (mortar, adobe, and structural ceramics), and 10 geological samples were selected for analysis from Monte Bernorio and El Cerrito (S4.1 in S1 File, Fig 4). Wheel-made pottery was further subdivided, where possible, into more detailed shaping categories (wheel-coiling or wheel-throwing), based on macro-observations and the orientation of inclusions in thin-section [68]. Due to the small dimensions of the pottery sherds, macro-trace analysis could not be conducted on each sherd, and therefore subdivision beyond the wheel-made category is speculative. The samples were analysed using a combination of Thin-Section Ceramic Petrography (TSCP), X-Ray Fluorescence Spectroscopy (XRF), X-Ray Diffraction (XRD), Scanning Electron Microscopy with Energy Dispersive Spectroscopy (SEM-EDS), and Inductively Coupled Plasma Mass Spectrometry (ICP-MS). A full description of the methodological procedures can be found in the S1 File. XRF and ICP-MS measurements are provided as parts per million in the S4.2-S4.4 in S1 File.

**Table 1:** Overview of the methodology and associated classes of information [adapted from 69]. XRF = X-Ray Fluorescence, SEM = Scanning Electron Microscopy, ICP-MS = Inductively Coupled Plasma–Mass Spectrometry, TSCP = Thin Section Ceramic Petrography, XRD = X-Ray Diffraction, Ternary = Ternary diagram, CV = Coefficient of Variation.

Technology	Manufacturing stage	Class of information	Technique	Analytical method	Comparative value
Hand-made, wheel-made ceramics, building materials	Raw material selection	Chemical composition	XRF, SEM, ICP-MS	Ternary	Provenance
				CV	Standardisation
		Mineralogical composition	TSCP, XRD	Fabric description	Provenance, clay recipes, fabric diversity (standardisation)
	Clay preparation	Kneading, purification, levigation	TSCP	Fabric description	Fabric diversity (standardisation)
Hand-made, wheel-made ceramics	Shaping	Properties of build	Photography	Macro-trace analysis	Diversity of technical skills
			TSCP	Orientation of voids	
	Heat treatment	Thermal transformation	XRD	Descriptive classification	Max. firing temperature
		Vitrification	SEM	Imagery	Max. firing temperature
		Coloration differences	Photography	Macroscopic observation	Firing atmosphere
	Finishing	Surface texture	Photography	Macroscopic observation	Surface treatment
		Paints	Photography, XRF	Macroscopic observation	Provenance, diversity of decoration

TSCP and XRF analyses were carried out at the University of Edinburgh archaeology laboratories, while XRD, SEM-EDS, and ICP-MS took place at the Durham Archaeomaterials Research Centre (DARC). Ceramic fabric classification took place utilising a polarising light microscope and fabrics were described utilising the fabric description system developed by Whitbread [70, 71] and modified by Quinn [30] (S2 File).

Statistical analyses of the XRF and ICP-MS data were conducted using R software package 4.0.2 (R Core Team) with package ggplot2 [72]. Due to budgetary constraints, we analysed a subset of samples using ICP-MS, which provides highly precise and accurate bulk chemical data using small amounts of sample. Selected samples reflect the spectrum of fabric groups identified by thin-section petrography. XRF provided a supplementary method for analysing the full dataset. The geochemical analysis focused primarily on comparing clay raw materials used in the production of hand-made and wheel-made pottery in order to compare clay procurement strategies and provenance associated with these different shaping modes. This comparison focuses on rare earth elements (henceforth REE), because these are a good indicator of the original composition of raw materials since they remain largely immobile during weathering and hydrothermal alteration [73–76]. Even though REEs are generally enriched in argillaceous sediments relative to most types of rock [77: 3, 78: 188], recent studies show that there is no fractionation of these elements from the firing process [74: 2389]. The REE contents were normalised to the Post Archaean Australia Shale (PAAS). Normalisation indicates whether sediments are enriched or deficient of REE relative to PAAS, which closely approximates the original REE content of the crust [73, 79]. We adopted an approach focused primarily on element ratios rather than absolute values to mitigate the impact of variations among the samples in volatile organic matter, known post-depositional impacts, and tempering with quartz, calcite, and sedimentary rocks [80]. The ratio of Light REE (La, Ce, Pr, Nd, Sm, Eu; LREE) to Heavy REE (Gd, Tb, Dy, Ho, Er, Tm, Yb, Lu; HREE) is governed by clay protolith(s) [74] and has been shown to remain stable in spite of the addition of tempering materials where these are similar to the original clay protolith(s) or are relatively deficient in trace elements [74: 2389]. The value is, therefore, useful for determining whether ceramics tempered differentially (utilising quartz, calcite, or feldspars) are derived from the same original source rocks.

Coefficient of variation (CV) measures are used to compare levels standardisation in ceramic pastes in the wheel-made and hand-made ceramics, following methods developed by Eerkens and Bettinger [81] and more recently employed for the comparison of different modes of potter’s wheel use in Bronze Age Anatolia by Fragnoli [44]. The CV has been utilised to assess variation in the relative proportions of elements measured through ICP-MS as a means of understanding paste standardisation, which can be seen as an important indicator of overall standardisation in raw material procurement and clay preparation [43]. The CV was calculated for each element from the ratio between the mean and standard deviation, expressed as %.

## Results

### Mineralogical and geochemical profile of El Cerrito samples

**Fig 5:**
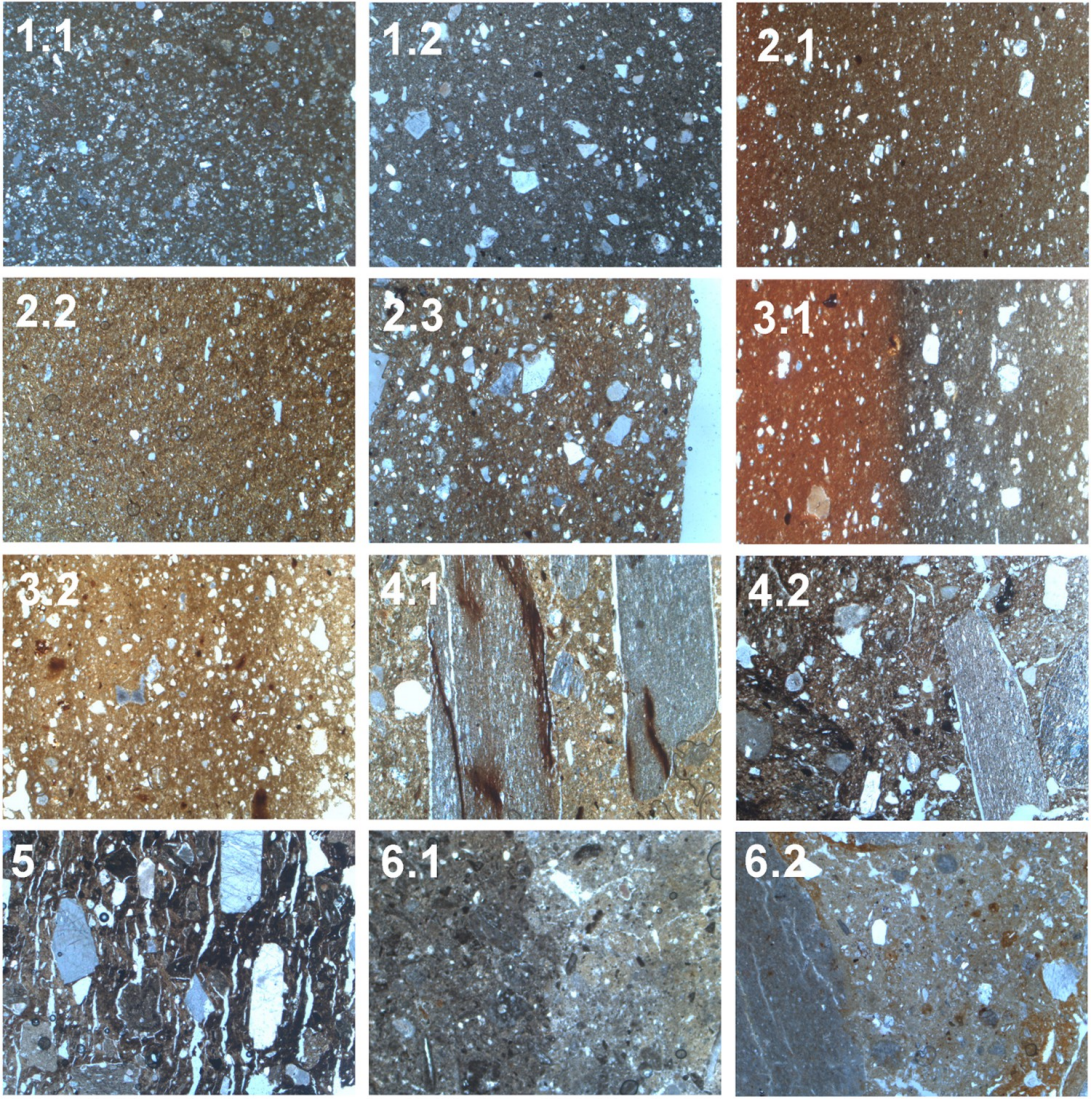
Microphotographs of ceramic fabric groups distinguished for El Cerrito (field of view of each microphograph = 0.3mm). 1.1: Calcareous clay, 1.2: Clay with quartz and micrite, 2.1: Fine micrite and quartzite (non-calcareous), 2.2: Fine quartz (non-calcareous), 2.3: Sandy temper, 3.1: Quartz-temper and micrite-rich fabric, 3.2: Oolitic limestone and ferruginous inclusions, 4.1: Shale tempered fabric, 4.2: Shale and grog tempered fabric, 5: Grog and calcite temper, 6.1: Calcareous building material, 6.2: Calcareous clay with oolitic limestone.

The fabrics distinguished through petrographic analysis at El Cerrito are summarised in Tables 2, 3 and Fig 5. For the samples identified as wheel-made, four fabric groups were distinguished, which were further divided into subgroups. The majority of the wheel-made pottery (*n* = 14) is made of non-calcareous clay with fine micrite, quartzite, and rare ferruginous inclusions (fabric 2.1). Non-plastic inclusions are very fine suggesting that clays were purified carefully, probably utilising levigation. The samples are fired in oxidising conditions resulting in bright orange and red surface colours. Incomplete oxidisation is visible in C1.10, C1.14, C6.5 and C7.1, resulting in a grey and orange zoning parallel to the margins of the sample. The inclusions are often distributed through the radial section in an ‘imbricate’ pattern suggestive of wheel-throwing, in which inclusions incline from the vessel wall to the centre of the section and are oriented more vertically at the centre [82]. In C1.3 and C6.2 inclusions are more randomly oriented, perhaps clustering together at the coil breaks, although the sample is too small to be certain. Possibly vessels were shaped through wheel-coiling and wheel-throwing. Fabrics 1.1, 1.2, 2.3 and 3.2 represent ‘loners’, fabrics represented by only one sample.

**Table 2:** Fabrics and associated samples from El Cerrito. Where possible, the presence of wheel-coiling or wheel-throwing has been indicated, with *n* = number of samples attributed to each fabric.

Type	Fabric	Description	Samples
Wheel-made	1.1	Calcareous clay (*n* = 1)	C1.5
Wheel-thrown	1.2	Clay with quartz and micrite (*n* = 1)	C9.1
Wheel-thrown, wheel-coiled	2.1	Fine micrite and quartzite (non-calcareous) (*n* = 14)	C1.1, C1.2, C1.3, C1.4, C1.10, C1.11, C1.12, C1.14, C1.15, C6.1, C6.2, C6.3, C6.5, C7.1
Wheel-made	2.2	Fine quartz (non-calcareous) (*n* = 2)	C1.8, C1.9
Wheel-made	2.3	Sand temper (*n* = 1)	C1.7
Wheel-made, wheel-coiled	3.1	Quartz-tempered in micrite-rich matrix (*n* = 5)	C1.6, C1.13, C6.6, C7.2, C8.1
Wheel-coiled	3.2	Oolitic limestone and ferruginous inclusions (*n* = 1)	C6.4
Hand-made	4.1	Shale tempered fabric (*n* = 11)	C3.1, C4.1, C4.2, C4.3, C10.2, C11.1, C11.2, C12.1, C13.4, C13.5
Hand-made	4.2	Shale and grog tempered fabric (*n* = 9)	C10.1, C12.2, C12.3, C13.6, C13.7, C13.8, C14.1, C14.2, C14.3
Hand-made	5	Grog and calcite temper (*n* = 3)	C13.1, C13.2, C13.3
Adobe from combustion chamber	6.1	Calcareous building material (*n* = 1)	CBM10
Hand-made ceramic building material used in combustion chamber	6.2	Calcareous clay with oolitic limestone (*n* = 3)	CBM1, CBM2.1, CBM2.2, CMB3, CBM4, CBM5

**Table 3:** Main features of the petrographic groups distinguished for ceramics from El Cerrito. Mi = micrite, Qu = quartz, FS = feldspar, Fe = ferruginous inclusions, Ca = calcite, OoLi = oolitic limestone, CP = clay pellets, Sh = shale, Gr = grog, She = shells.

Fabric	Inclusions in coarse fraction, ordered from predominant to rare	Inclusion max size/amount
1.1	Mi, Qu, FS, Fe	0.3mm/20%
1.2	Qu, FS, Mi, Ca	0.4mm/15%
2.1	Mi, Ca, Qu, FS, Fe	0.3mm/5-12%
2.2	Qu, FS, Fe	0.25mm/5%
2.3	Qu, FS, Mi, Fe	0.6mm/24%
3.1	Qu, FS, Mi, Fe	1.25mm/5-18%
3.2	OoLi, Mi, Fe, Qu, FS	0.75mm/16%
4.1	Sh, Mi, Fe, CP, Qu, FS, Ca	11.0mm/20-30%
4.2	Sh, Mi, Fe, CP, Gr, Qu, FS, Ca	11.0mm/20-30%
5	Ca, Gr, Fe, CP, Sh, Qu, FS	1.75mm/35%
6.1	Ca, CP, Qu, She	5.0mm/10%
6.2	OoLi, Mi, Qu, Ca	13.0mm/30%

The twenty-three samples of coarse hand-made pottery have a clearly distinct fabric, characterised by the presence of shale, grog, and dolomite temper. The majority of the samples (*n* = 11) contain coarse shale (<11.0mm) with lamellar microstructure with coarser (±0.1mm-wide) grains of rounded sericite mica inclusions (fabric 4.1). The fabric has a heterogeneous matrix due to the presence of clay pellets and elongate streaking. Shale inclusions are often strongly aligned to the vessel walls, and in some cases seem to follow coil or slab joints. Rounded micrite inclusions in this fabric often contain oolites and other microfossils indicating a local oolitic limestone origin. SEM imagery of ceramics of the shale tempered fabric (4.1) and wheel-thrown pottery (fabric 2.1) demonstrates that these fabrics underwent a similar heat treatment leading to sintering of the matrix (S2.1 Fig in S2 File). Fabric 4.2 is similar in composition to fabric 4.1 but also contains grog with a greyish colour. Grog fragments (<5.0mm) contain quartz, mica, and rare sandstone or shale inclusions. Possibly grog derives from finer tempered, reduced fired hand-made pottery. Grog also appears in fabric 5 alongside rhombic calcite fragments with parallel twinning at a 120° angle. The grog contains well-sorted calcite and dolomite inclusions suggesting ceramics derived from a different temper source from the shale-grog in fabric 4.2.

The clays used in the construction of the kiln (including fired adobe fragments from the kiln wall and fired ceramics utilised in the construction of the dome) represent yet another fabric group, characterised by the calcareous clays and presence of microfossils. Fabric 6.2 has been tempered with rounded limestone pebbles, which probably derived from loose alluvial deposits.

XRD analysis was conducted on 13 ceramic samples (S3 File). The results indicate that smectite-illite rich clays were used in both hand-made and wheel-made ceramics. Ceramics containing shale temper (fabric 4) also show a peak for temperature-altered vermiculite, which should occur when firing temperatures exceed 700°C. The presence of illite in all samples indicate that firing temperatures generally did not exceed 950–1050°C [83]. Sample C8.1 (fabric 3.1) contains ghelenite, which forms at the expense of silica, phyllosylicates, and calcite at temperatures between 700–1150°C [83]. The maximum temperatures achieved within the kiln are thus likely to have fluctuated between 700 and 950°C. The presence of vermiculite in fabric 4 samples and some other distinguishing features (fully oxidized cores, hardness of fabric relative to fabric 5 samples, and equal sintering of the matrix to the wheel-made samples demonstrated by SEM) suggests that these were fired in the kiln. Thus, hand-made coarse ware of fabric 4 seems to have been produced alongside wheel-made ceramics, using the same basic clay materials, while hand-made pottery of fabric 5 is not related in composition or firing conditions so could have been imported to the site as cooking ware.

There are clear differences in the clay recipes utilised in the production of hand-made and wheel-made pottery at El Cerrito. Wheel-made pottery contains fine inclusions, generally dominated by quartz. Hand-made pottery contains either coarse shale (fabric 4), or grog and crushed calcite (fabric 5). Ceramic building materials contain similar inclusions, while building plasters and adobe are high in calcareous material. Given the reduced firing observed in the hand-made samples of fabric 5, and due to the general lack of shale temper in this fabric, it is possible that these represent vessels produced off-site. Samples of fabric 5 also have a higher density of elongate voids, which result from the shrinkage of the clay during firing in mixed atmospheres. Despite the evidence for standardisation among wheel-made pottery from contemporary sites in northern Spain, reflected in the use of fine non-calcareous fabrics with fine silicate inclusions [55, 56, 84–86], subtle variation among the wheel-made fabrics from El Cerrito could point to diverse clay selection strategies, or variation within the same clay source. Geochemical analyses below will further address the issue of fabric standardisation and provenance.

**Fig 6:**
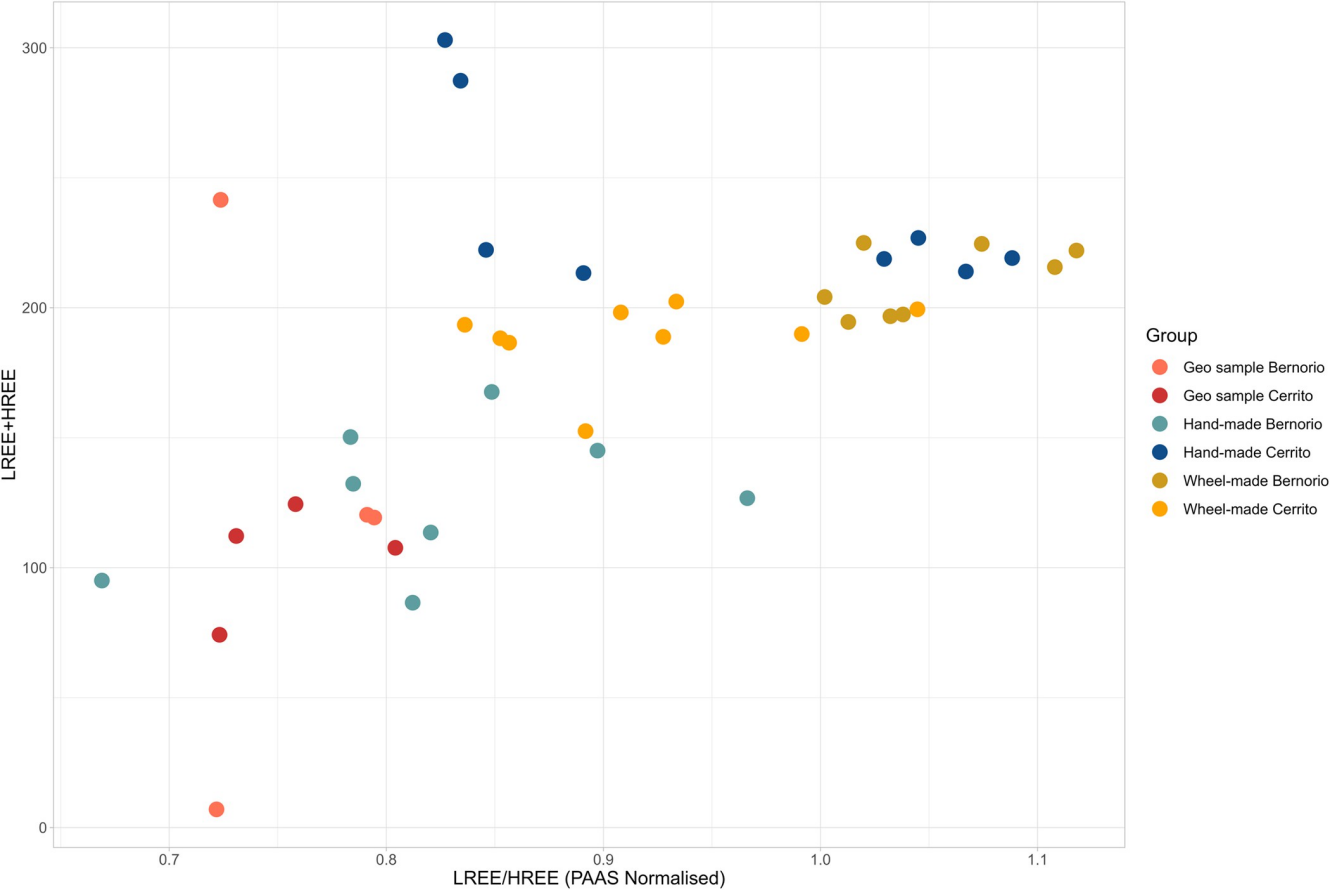
Plot of LREE+HREE and LREE/HREE (PAAS Normalised) ratio. The overall sum (y-axis) reflects relative clay richness while LREE/HREE (x-axis) reflects inputs independent of temper.

**Fig 7:**
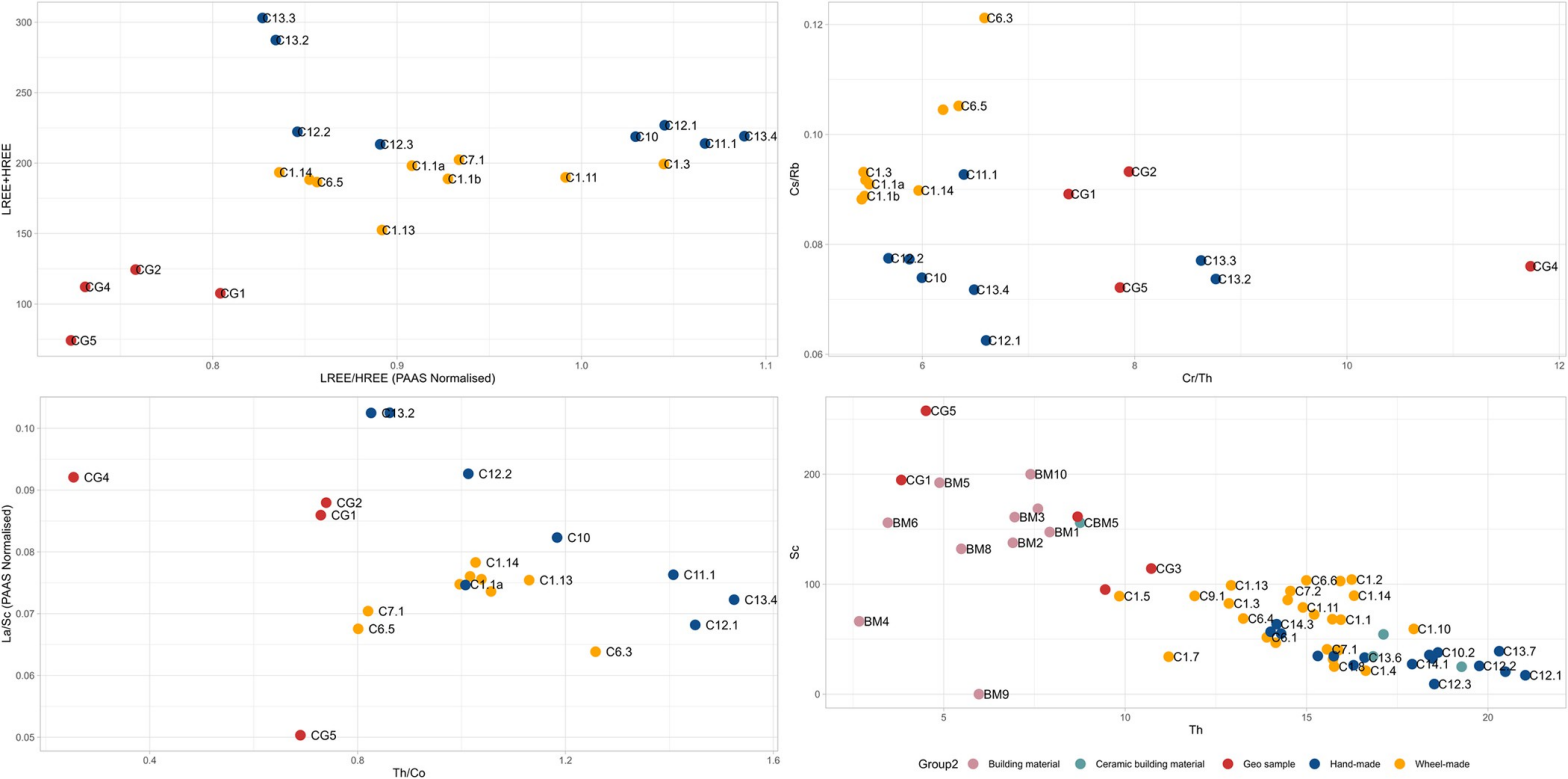
Bivariate plots of ICP-MS and XRF data from El Cerrito. A) Total REE and ratio between LREE/HREE (PAAS Normalised) indicating commonalities in hand-made and wheel-made pottery clay materials along x-axis. B) plot of Cs/Rb and Cr/Th ratios, C) plot of La/Sc (PAAS Normalised) and Th/Co ratios. D) plot of XRF readings of Scandium and Thorium (ppm) suggesting overlap between hand-made pottery, wheel-made pottery, and ceramics used in the construction of the kiln and a divergence between the geological samples and architectural clays [73].

**Fig 8:**
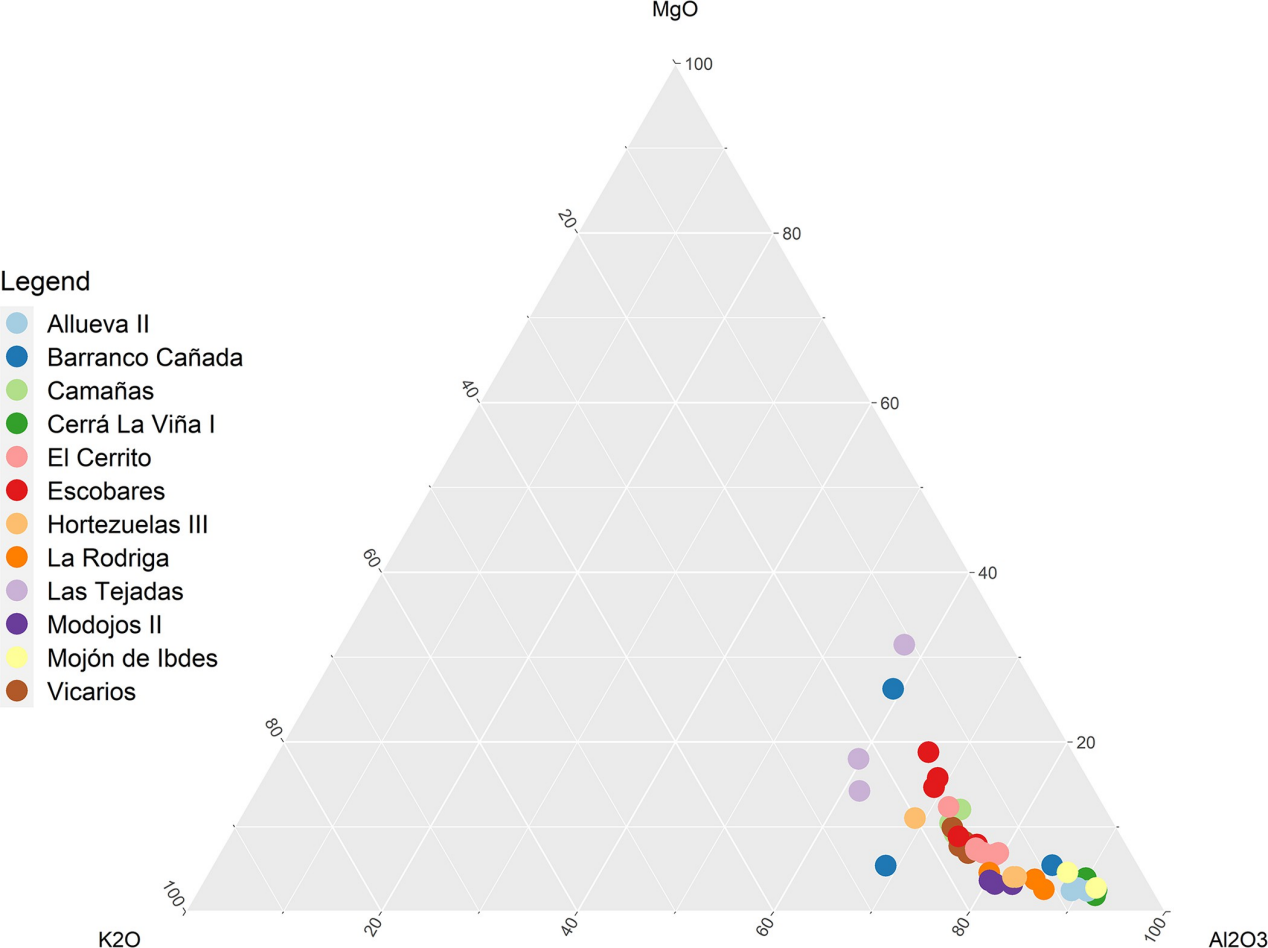
Ternary diagram of MgO, K_2_O and Al_2_O_3_ of wheel-made pottery samples from El Cerrito and surrounding sites [55, 56], showing no clear inter-site clustering in ceramic materials although samples from Barranco Cañada and Las Tejadas are outliers.

The XRF and ICP-MS data, visualised in Figs 6 and 7, indicate that hand-made and wheel-made pottery from El Cerrito could have been produced from similar clays. Fig 6 shows the distribution of ICP-MS analysed samples based on the sum of LREE and HREE, and LREE/HREE (PAAS Normalised), where the latter (x-axis) reflects inputs independent of temper, while the sum of HREE and LREE (y-axis) is a marker of clay richness. Hand-made and wheel-made samples from El Cerrito show no clustering along the x-axis and could therefore be produced from similar clays. Part of the scatter in the data along the x-axis can be explained by the presence of grog in some of the hand-made samples, because grog has a variable trace element profile rendering it unsuitable for an element ratios approach. However, it is likely that grog tempered pottery reflects a different *chaîne opératoire* altogether given the different firing practices and tempering strategies utilised (see previous section). Fig 8 provides further detail into the effects of this.

A similar pattern appears in Fig 7A, in which El Cerrito samples are analysed independently. The figure shows that hand-made pottery clusters in two groups along the x-axis, indicating there are two broad provenance groups in this class of ceramics. These pertain to grog and calcite tempered cooking pottery (C13.2, C13.3; fabric 5) and shale and grog tempered oxidised storage vessels (C12.2, C12.3; fabric 4.2). The grog tempered samples are enriched in REE, which is a probable effect of the presence of crushed ceramics of different mineralogical composition than the matrix.

Clay materials for wheel-made pottery seem relatively heterogeneous with no clear clustering along the x-axis of Fig 7A. Geological samples largely cluster separately from the ceramic samples. A plot of Th/Co and La/Sc (Fig 7C), which provides a way to evaluate and classify geological inputs in the clay, is inconclusive while Fig 7B (Cs/Rb and Cr/Th) suggests geological source CG1 is a close match with the ceramic samples. TSCP analysis of CG1 demonstrates that the clay contains very fine quartz and feldspar inclusions, next to ferruginous inclusions and rare fine mica. The clay is naturally fine and probably needed little processing. The clay body is heterogeneous with greyish streaking, which is not clearly visible in the fabrics of wheel-made ceramics, but does occur in the hand-made samples of fabric 4.1 (C3.1, C10.2, C12.1, C13.5). It is therefore likely that clays utilised in the production of wheel-made pottery were more carefully processed than clay used in the local production of hand-made pottery. Based on petrographic similarities and due to location of the clay source near the site it is most likely that clays for the production of wheel-made pottery and hand-made pottery of fabric 4 derived from the alluvial deposits around CG1. Fig 7A and 7B show that grog and calcite tempered pottery (fabric 5) reflects a separate group, with samples C13.2 and C13.3 clustering away from the other samples. Because these samples have different REE compositions from the rest of the group (particularly along the Cr/Th axis in Fig 7B) they may reflect a different raw material group from the other samples, in both temper and potentially clay provenance.

XRF readings of Scandium and Thorium (S4.4 in S4 File) show that geological and building samples are rich in materials associated with calcareous deposits, while the ceramic samples reflect the mixed alluvial deposits around the site. The separation between hand-made and wheel-made samples could be an effect of differential tempering, with hand-made samples containing relatively more shale from a non-calcareous source. The ICP-MS readings were compared with ICP-AES analyses conducted on wheel-made pottery from pre-Roman sites in the surroundings of El Cerrito (Fig 2), published in Igea et al. [55] and Saiz et al [56]. Ternary and PCA analyses show no clear clustering within this comparative dataset, confirming the general approach to clay sourcing and preparation between these sites was very similar (Fig 8).

Following Fragnoli [44] we utilised the ICP-MS data to examine geochemical standardisation within the hand-made and wheel-made samples. Standardisation in ceramic fabrics can contribute to assessing the overall level of standardisation within the ceramic manufacturing process [44, 87], although several pre-and post-manufacture processes can affect the geochemical signatures of ceramics [88]. Standardisation within the wheel-made category of ceramics produced at El Cerrito and surrounding sites is suggested by the lack of clear inter-site clustering based on MgO, K_2_O, and Al_2_O_3_ content (Fig 8). These sites shared a similar approach to clay selection and preparation, preferring non-calcareous clays with fine silicate inclusions, purified of any inclusions larger than 0.6mm [56].

**Fig 9:**
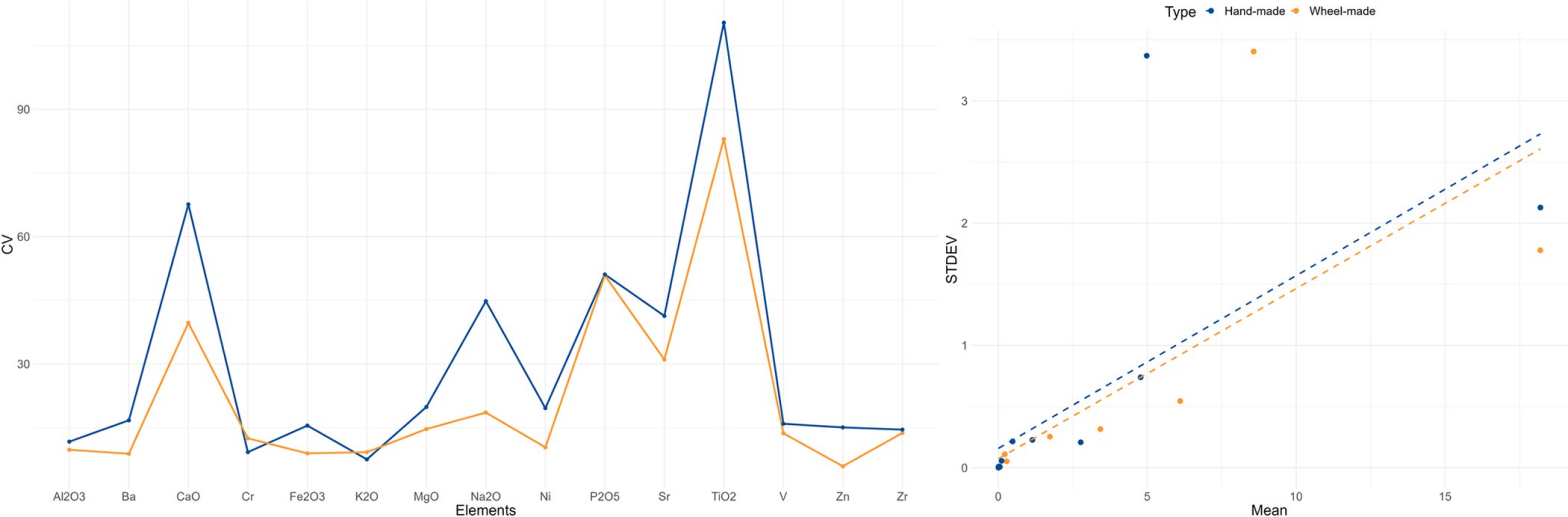
Coefficient of variation calculated for each element (based on ICP-MS data) within the hand-made and wheel-made categories at El Cerrito (left). Relationships between mean (x-axis) and standard deviation (y-axis) for all chemical elements.

At El Cerrito, however, this standardisation within the wheel-made samples is not greatly supported by the coefficient of variation (Fig 9). The elemental CVs of the wheel-made pottery (orange line) is only marginally lower than that of the hand-made samples (blue line), suggesting that geochemical variability within this category is almost equal to that within the hand-made pottery samples. A scatter plot of the relationship between the mean and the standard deviation provides no further information. Following Eerkens and Bettinger [81], steeper regression lines indicate more variation in elemental concentrations. Fig 9 shows, however, that the regression lines are more or less equal, suggesting a similar geochemical variability within the hand-made and wheel-made categories.

The relatively high CVs for elements in the wheel-made category could be explained by the fabric diversity identified through TSCP. Three different wheel-made fabrics have been distinguished suggesting that, despite the general absence of tempering, clay mixtures were relatively varied. It is likely that the observed variation corresponds to natural variation in the local clay beds of the Laguna del Cañizar, which borders on diverse geologies. This result, however, suggests that from a geochemical perspective hand-made and wheel-made pottery are nearly equally standardised at El Cerrito.

### Mineralogical and geochemical profile of Monte Bernorio samples

**Fig 10:**
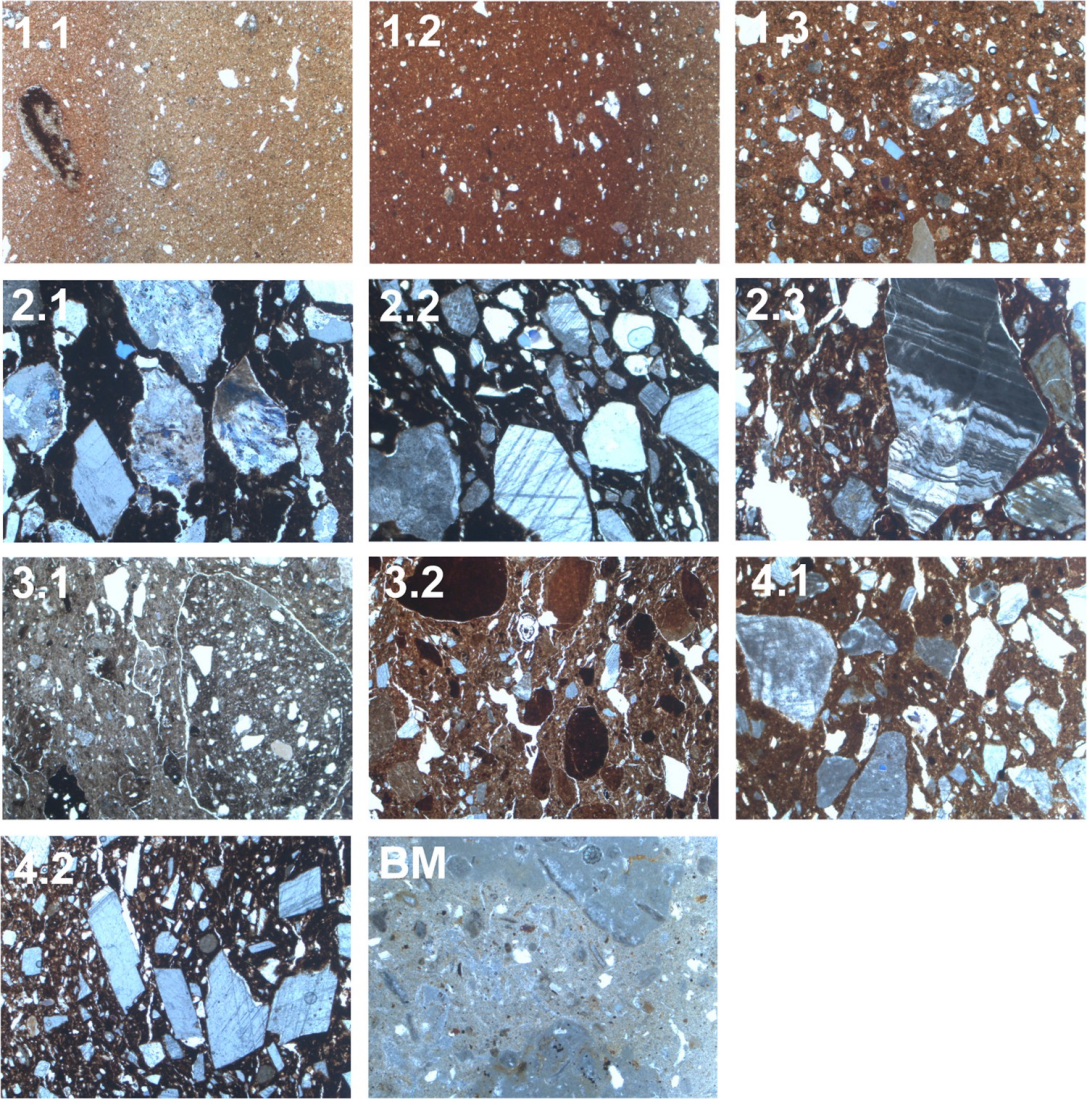
Microphotographs of ceramic fabric groups distinguished for Monte Bernorio (field of view of each microphotograph = 0.3mm). 1.1: Fine sand fabric, 1.2: Moderately ferrous clay with fine sand, 1.3: Sand temper, 2.1: Crushed gypsum, 2.2: Crushed metamorphic inclusions, 2.3: Calcareous clay with sparry limestone inclusions, 3.1: Clay pellets/grog, 3.2: Clay pellets/grog and calcareous inclusions, 4: Calcite temper, 5: Calcareous clay with microfossils.

The fabrics distinguished at Monte Bernorio are summarised in Tables 4, 5 and Fig 10. The wheel-made pottery sampled is generally undecorated, aside from a thin orange or brown slip visible on the pale surface of several of the samples (Fig 4). No diagnostic macroscopic features could be discerned which would provide information about the shaping process, aside from the occasional presence of rilling, indicating that a rotary device was used. The inclination of inclusions in the wheel-made samples is often diagonal, vertical, or randomly oriented, suggesting that the pottery would have been predominantly wheel-coiled rather than wheel-thrown. Wall-thicknesses of the wheel-made samples are also a bit wider than at El Cerrito, generally exceeding 1 cm.

**Table 4:** Fabrics and associated samples from Monte Bernorio, with *n* = number of samples attributed to each fabric.

Type	Fabric	Description	Samples
Wheel-made	1.1	Fine sand fabric (*n* = 11)	MB1.2, MB1.3, MB1.4, MB1.5, MB1.6, MB1.7, MB1.8, MB1.11, MB1.13, MB1.16, MB1.19
Wheel-made	1.2	Moderately ferrous clay with fine sand (*n* = 7)	MB1.1, MB1.9, MB1.15, MB1.17, MB1.18, MB1.20, MB1.21
Wheel-made	1.3	Sand temper (*n* = 2)	MB1.10, MB1.12
Hand-made	2.1	Crushed gypsum (*n* = 1)	MB2.20
Hand-made (incised dec)	2.2	Crushed metamorphic inclusions (*n* = 1)	MB2.3
Hand-made	2.3	Calcareous clay with sparite (*n* = 1)	MB2.10
Hand-made	3.1	Clay pellets/grog (*n* = 3)	MB2.16, MB2.25, MB2.27
Hand-made	3.2	Clay pellets/grog and calcareous inclusions (*n* = 2)	MB2.17, MB2.23
Hand-made	4	Calcite temper (*n* = 19)	MB2.1, MB2.2, MB2.4, MB2.5, MB2.6, MB2.7, MB2.8, MB2.9, MB2.11, MB2.12, MB2.13, MB2.14, MB2.15, MB2.18, MB2.19, MB2.21, MB2.22, MB2.24, MB2.26
Building material	5	Calcareous clay with microfossils (*n* = 3)	MBMW1.1, MBMW1.2, MBMW1.3

**Table 5:** Main features of the petrographic groups distinguished for ceramics from Monte Bernorio. Mi = micrite, Qu = quartz, FS = feldspar, Fe = ferruginous inclusions, Ca = calcite, CP = clay pellets, Gr = grog, Gy = gypsum, Do = dolomite, Mm = metamorphic inclusions.

Fabric	Inclusions in coarse fraction, ordered from predominant to rare	Inclusion max size/amount
1.1	Qu, FS, Mi, Fe	0.75mm/3-5%
1.2	Qu, FS, Mi, Fe	0.8mm/5-15%
1.3	Qu, FS, Mi, Fe	0.75mm/25%
2.1	Gy, Ca, Mi, Fe	2.5mm/50%
2.2	Mm, Do, Qu, FS, Fe	2.5mm/40%
2.3	Ca, Mi, Fe, Qu, FS	2.25mm/40%
3.1	CP, Gr, Ca, Mi, Qu, FS	2.25mm/40%
3.2	CP, Gr, Ca, Mi	1.5mm/30%
4	Ca, Mi, Qu, CP, Fe, FS, Gr	3.5mm/30-40%

Petrographic analysis has provided evidence for one fabric group in the wheel-made category, which could be subdivided further into three subgroups (1.1, 1.2, and 1.3). In general, samples of fabric 1 are characterised by a homogeneous, non-calcareous clay containing fine quartz, micrite, and feldspar inclusions, which are generally larger than at El Cerrito (<0.8mm). The sample of fabric 1.2 diverges by containing more quartz in fine fraction and the matrix is also more ferrous. It is possible that variation between fabrics 1.1 and 1.2 relates to natural differences in the same clay source. There is no patterning in the type of fabric and the specifics of the surface treatment. Fabric 1.3 contains coarser, well-sorted angular quartz and feldspar. The bimodal grain-size distribution of this fabric suggests that these non-plastic inclusions were added as temper.

Three fabric groups were distinguished within the hand-made category. Loners are represented by the subgroups of fabric 2. These are samples with (moderately) calcareous matrices containing crushed gypsum (2.1), crushed metamorphic inclusions (2.2), and calcareous clay with sparite inclusions (2.3). Samples of fabric 2.1 and 2.2 have a generally similar matrix suggesting that different tempers were added to similar clays. Fabric 2.3 has a calcareous matrix, which was also tempered with crushed sparry calcite samples of fabric 3 clearly differ due to the presence of clay pellets and grog. In fabric 3.2, crushed calcite and grog tempers are combined. Grog tempered pottery is generally porous and fired in reduced atmospheres, while both oxidising and reducing atmospheres are common in the calcareous tempered fabrics. Fabric 4 is represented most frequently among the hand-made samples, being characterised by the presence of coarse angular calcite inclusions in a low to non-calcareous matrix.

XRD analysis conducted on 12 ceramic samples demonstrate that non-calcareous, illite-montmorillonite-rich clays were used for the production of wheel-made pottery while hand-made pottery generally contained calcite, quartz, and illite (S3 File). Firing temperatures generally will not have exceeded 800°C except for in sample MB1.3 (fabric 1.1) in which diopside has formed. Diopside typically forms in a temperature range between 800–950°C [83].

**Fig 11:**
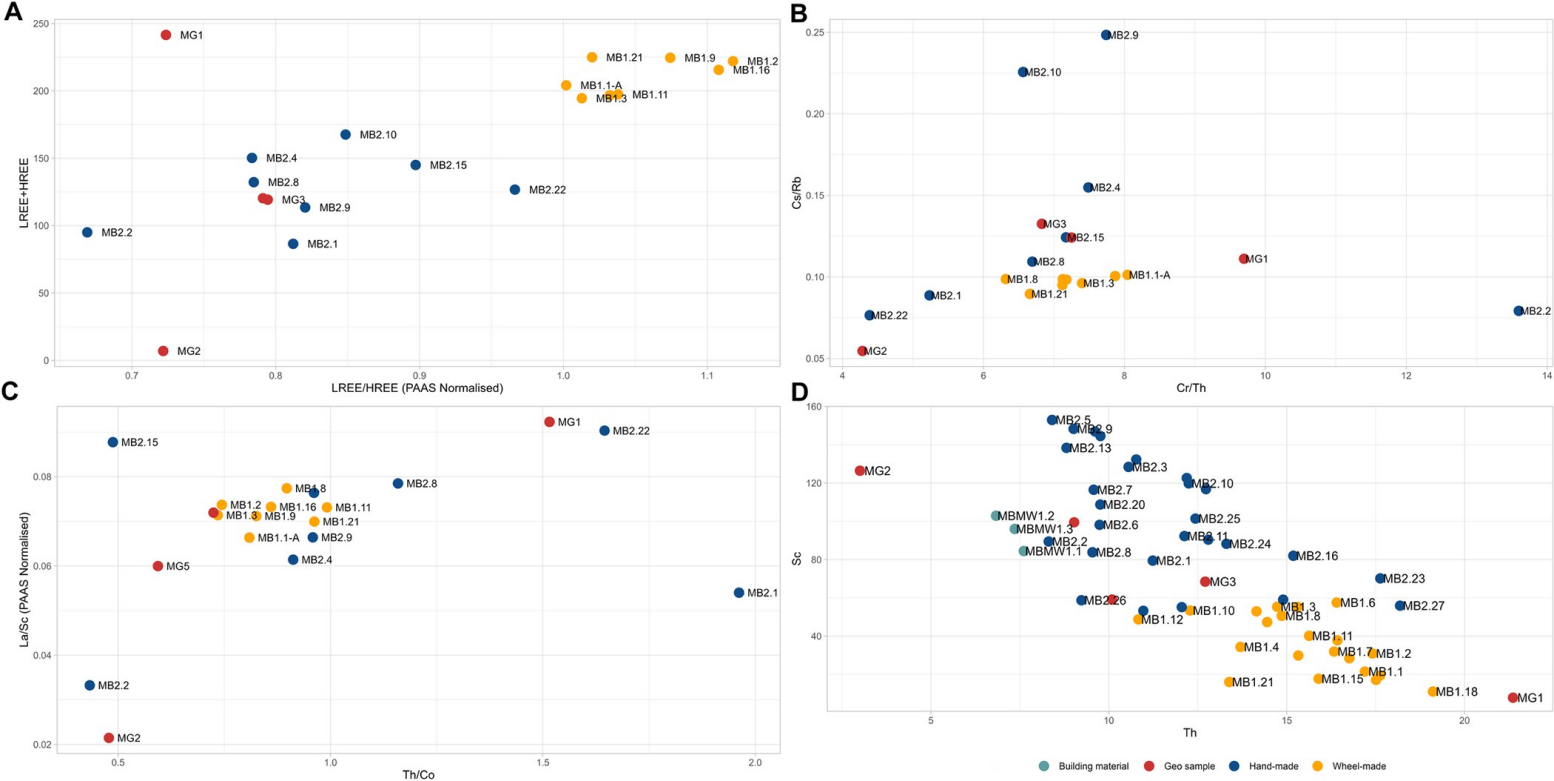
Bivariate plots of ICP-MS and XRF data from Monte Bernorio. A) Total REE and ratio between LREE/HREE (PAAS Normalised) indicating differential clustering along x-axis, suggesting different clays were used. B) plot of Cs/Rb and Cr/Th ratios, C) plot of La/Sc (PAAS Normalised) and Th/Co ratios. D) plot of XRF readings of Scandium and Thorium (ppm) suggesting wheel-made pottery derives from detrital clays while wheel-made pottery is more likely to derive from ophiolite clays [73].

The chemical profile provides evidence for the source areas of the clays used in the production of pottery from Monte Bernorio, suggesting that hand-made and wheel-made pottery were produced from different clays. Fig 11A and 11D show that hand-made and wheel-made samples cluster in different areas. Geological samples MG1 and MG2 cluster less clearly with the ceramic samples. MG1 is a ferruginous terra rossa clay that formed between limestone rocks in the western profile of Area 4. MG2 derives from a calcareous deposit east of the site. The samples of hand-made pottery, however, closely match with MG3 and MG4, which are samples deriving from Keuper deposits some 7km west of the site, next to the Embalse de Aguilar and Pisuerga River. These deposits contain evaporites and dolomite, which also appear in the fabrics of hand-made pottery.

Fig 11A demonstrates that hand-made pottery clusters with the geological samples relating to the Keuper clays (MG3 and MG4), while wheel-made pottery clusters at the right-hand side of the x-axis. This suggests that hand-made pottery was made from local Keuper clays, while wheel-made pottery derives from an unidentified, non-calcareous source. Fig 11B and 11C confirm the homogeneity of the wheel-made pottery compared to hand-made pottery. The XRF plot of Scandium and Thorium shows that building materials have a broadly similar geochemical composition as the hand-made samples, confirming the high calcareous content of hand-made pottery and building materials as opposed to the wheel-made pottery. Microfossils common in the building materials fabric are not present in the calcite-tempered pottery, however, suggesting no overlap between raw materials used in both categories of samples.

**Fig 12:**
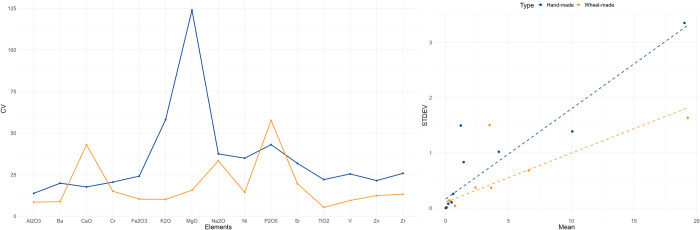
Coefficient of variation calculated for each element (based on ICP-MS data) within the hand-made and wheel-made categories at Monte Bernorio (left). Relationships between mean (x-axis) and standard deviation (y-axis) for all chemical elements.

The coefficient of variation of the chemical elements for the production of hand-made and wheel-made pottery provide insights into the standardisation of clay processing at Monte Bernorio. Hand-made pottery has a less standardised chemical composition than wheel-made pottery, as demonstrated by the generally lower CV of the chemical elements within the wheel-made category, and the steeper regression line of relating to the comparison of the mean and standard deviation calculated for each chemical element in the hand-made category (Fig 12). Fabric standardisation is also suggested by the clustering of wheel-made samples in Fig 11, confirming the relative chemical homogeneity within fabric 1 compared to the more heterogeneous hand-made pottery fabrics.

## Discussion

### Provenance of clays for hand-made and wheel-made pottery

The comparison of the fabrics of hand-made and wheel-made pottery largely points to the persistence of polarised ceramic technologies at the level of raw material procurement, preparation, and firing treatment, relating to the workshop-based production of largely wheel-made pottery, and the production of hand-made pottery. At both Monte Bernorio and El Cerrito, wheel-made pottery is distinctive from the broader assemblage through the fine texture of its fabrics, containing only rarely quartz and feldspar temper. The introduction of the workshop-based production of wheel-made, Celtiberian-style pottery thus related to a fixed approach to clay sourcing and preparation, based on the careful purification and limited tempering of non-calcareous clays. This is demonstrated by the close geochemical and petrographic similarity of samples from sites surrounding El Cerrito and by the generally similar clay recipes used in the production of Late Iron Age ceramics at other sites on the Central Iberian Plateau [84–86, 89, 90]. This specific way of preparing the clays was thus introduced as a concept and part of a package of technological innovations, including the potter’s wheel and the double-chambered updraught kiln.

The geochemical signature of hand-made and wheel-made pottery also indicates the polarisation between such ceramics. At Monte Bernorio, the REE compositions of hand-made and wheel-made pottery demonstrates that different clays were used in their production, with hand-made pottery utilising clays deriving from local deposits such as the Keuper red beds. Wheel-made pottery was made from non-calcareous, illite-rich clays that do not match the local, calcareous geology. Rather than being imported to the site, however, it is likely that wheel-made pottery was produced on the *oppidum*, as suggested by the discovery of unfired wheel-made pottery and clay in the ditch south of the site as well as a piece of the grill (Fig 1) part of a double-chambered updraught kiln [61]. The non-local signature of the clays and the possibility of local production suggest that unfired clays were imported to the site, where they were prepared for the production of ceramics. Archaeometric evidence from nearby sites indicates that the clay recipes of hand-made pottery formed part of a broader technological tradition, in which crushed calcite and grog was used to temper ceramics [65]. Continuity in the production of hand-made pottery utilising widespread clay recipes thus suggests that such ceramics retained a level of importance in Late Iron Age society, as also indicated by the greater typological variability, with thin-walled and decorated pieces suggesting such vessels were used for display instead of cooking exclusively.

At El Cerrito, instead, geochemical groupings among the hand-made and wheel-made samples are more difficult to discern, pointing to an overlap in the extraction of clays for hand-made and wheel-made pottery, except for in the grog and calcite-tempered samples (fabric 5). Fabric 5 ceramics might have been brought to the site as cooking ware, while shale tempered hand-made samples (fabric 4) were produced in the workshop context. Grog and calcite tempered pottery is generally well-suited to dealing with thermal shock resistance [91], while the shale tempered pottery could reflect a means of toughening the clay for the production of large storage vessels. Again, the different tempering recipes observed in the hand-made category reflect a separation in production tradition, based within and outside of the workshop context. Thus, rather than drawing upon existing skills and knowledge regarding to clay preparation and tempering, potters operating in the workshops experimented with different clay preparation procedures for the production of hand-made pottery.

Architectural features such as pieces of adobe wall from the kiln at El Cerrito, and the pieces of fired adobe from Monte Bernorio are made of highly calcareous clay, of different provenance than the ceramic clays. Pieces of pottery embedded in the kiln at El Cerrito have yet another fabric, comprising calcareous clays tempered with pebbles. Clays utilised, however, have a broadly similar chemical signature as the hand-made and wheel-made sherds, indicating that such ceramics were made locally, utilising a specialised clay recipe differing from the shale-tempered, hand-made storage jars produced on-site.

### The organisation of ceramic production

The separation between the technological procedures underpinning the production of hand-made and Celtiberian-style pottery suggests a separation between production in the domestic and workshop spheres. Although there is currently no evidence for the location or scale of production of hand-made pottery in Late Iron Age northern Iberia, the quantities of such ceramics and the quality of decoration and finish at Monte Bernorio shows that such ceramics retained an important role in daily activities, despite the uptake of Celtiberian-style fine wares. It is therefore unclear why the *oppidum* also made a transition to workshop production. Domestic production is often considered to reflect small-scale production for local or domestic use, while workshop-produced pottery could correspond to market driven production for trade [11, 15, 92–95]. However, given the broad evidence for local production of Celtiberian-style pottery at multiple centres across the northern Iberian Plateau, the local production of Celtiberian-style pottery instead may have marked a way for populations across this region to take part in shared consumption practices rather than trade exclusively.

Based on findings from a pottery workshop at Mas de Moreno (Foz-Calanda, Teruel), Alexis Gorgues (2007) suggests that workshops might have operated on a seasonal rather than full-time basis [53]. Seasonal production by itinerant crafts people could also explain the utilisation of imported clays for the production of wheel-made pottery at Monte Bernorio. In this scenario, itinerant potters would bring along clays they saw as suitable for ceramic production rather than using locally available raw materials and recipes. Intermittent production by itinerant craftspeople would also explain the high levels of standardisation of clay recipes for the production of Celtiberian-style ceramics more broadly, as this technological homogeneity might have resulted from the transmission of skills within a small pool of specialised potters, organising themselves as a closed technological system [96].

El Cerrito is located in a landscape with dispersed pottery workshops and few other, larger, settlements. The high (geochemical) uniformity between the wheel-made pottery fabrics from such nearby sites suggests again that skills and knowledge were tightly managed and transmitted within a closed technological system. It is likely that potters were part of a shared production system equalling nucleated corvée [12], in which labour, though operating on a seasonal basis, was attached to an institution such as an elite or mercantile system, producing for a specific purpose (i.e. interregional trade). Seasonality in the production of ceramics at El Cerrito could explain the relatively low geochemical standardisation of wheel-made compared to hand-made pottery. Following observations by Fragnoli [44], production rates affect raw material supply and processing, leading to and increasing standardisation of the clay recipes utilised in the workshop-based production of wheel-made pottery. The seasonal production of pottery might instead have led to minor variations in the sourcing of clays prior to shaping, causing the minute variations in sorting and size of inclusions within the wheel-made category of ceramics. As such, workshop-based pottery production remained a relatively exclusive task, conducted by a limited number of expert craftspeople operating on a seasonal basis. The full-time mass production of ceramics thus seems a process emerging in Roman contexts, during which the scale of production of domestically produced hand-made pottery gradually declined.

## Conclusion

The archaeometric analysis of ceramic raw materials for the production of pottery and kiln architecture has shown that the emergence of workshop-based pottery production in the Iberian Peninsula formed part of a heterogeneous process, which was largely cut-off from existing modes of pottery production. Potters operating in the workshops introduced new clay recipes for the production of wheel-made pottery, and, as shown by the evidence from El Cerrito, invented new tempering practices for producing hand-made coarse ware. The results suggest that workshop modes of pottery production in Late Iron Age northern Iberia emerged as a discontinuous process, enacted by a small group of specialist potters that operated as a closed technological system. Our study demonstrates the importance of comparing different clay crafts using archaeometric methods in order to understand the transmission, standardisation, and organisation of technological skills during the shift to workshop modes of pottery production, revealing similarities and divergences at microscopic and elemental scales.

## Supporting information

S1 File Methodology. (PDF)

S2 File Ceramic petrographic fabric descriptions. (PDF)

S3 File XRD spectra. (PDF)

S4 File Geochemical data. Excel file with raw data in separate sheets; S4.1: table with samples and descriptions, S4.2: ICP-MS raw data in parts per million (ppm), S4.3: XRF raw data for Monte Bernorio samples in ppm, S4.4: XRF raw data for El Cerrito samples in ppm, S4.5: CV data for Monte Bernorio samples based on ICP-MS weights, S4.6: CV data for El Cerrito based on ICP-MS weights, S4.7: combined ICP-MS data of wheel-made samples for Fig 8.
(XLSX)

S5 File R code. (R)
