# Supplementary material for: Capturing technological crossovers between clay crafts: An archaeometric perspective on the emergence of workshop production in Late Iron Age northern Spain
Source: PLoS One. 2023 May 5;18(5):e0283343. doi: 10.1371/journal.pone.0283343 (PMC10162541; doi:10.1371/journal.pone.0283343)
Supplement: S1 File — (PDF) [file pone.0283343.s001.pdf]

Appendix 1 – Methodology

Thin-Section Ceramic Petrography

111 of the samples were selected for Thin Section Ceramic Petrography. Ceramic thin sections were prepared and analysed at the School of History, Classics and Archaeology of the University of Edinburgh. The first author conducted ceramic fabric classification utilising a GX polarising light microscope. Fabrics were described utilising the fabric description system developed by Whitbread (1, 2), modified by Quinn (3). Inclusion sizes were estimated under the microscope and percentages were measured using ImageJ software (4). Microphotographs were made using a Leica MC170 HD polarising microscope and camera. Fabric descriptions and microphotographs are in Appendix 2 and 3.

X-Ray Fluorescence

All samples (n = 121) were analysed using X-Ray Fluoresence to analyse their chemical composition. XRF has been selected as a supplementary method to ICP-MS because this provides a cheaper alternative and thus has allowed us to analyse all samples instead of a selection. We focus on the Thorium/Scandium readings as these have been shown to allow for distinguishing between different clay bodies (5). XRF values is provided in Supplementary File S4.

Sample preparation and analysis took place at the University of Edinburgh. Building materials and ceramic samples were ground in a mortar and sieved through a 355mu grid laboratory test sieve. Powder was placed in a sample cup before analysis. XRF analysis was conducted using a Thermo Scientific Niton FXL, FM-XRF analyser using the soil setting, using two readings of 180 seconds each. The averages of these two readings were utilised in statistical analyses.

Inductively Coupled Plasma – Mass Spectrometry

ICP-MS was chosen as the method of analysis for distinguishing groupings on non-temper materials within the fabrics of 32 pottery samples and 8 geological samples. ICP-MS has been selected because it provides highly precise and accurate bulk chemical data using small amounts of sample. The samples were prepared at the Durham Archaeomaterials Research Centre (DARC). Sample preparation involved the extraction of 100 mg of powder from across the profile of each sherd using a 12-volt dental drill, fitted with a 2mm diameter solid tungsten carbide bit. The powders obtained were acid digested using hydrofluoric acid and analysed by ICP-MS at the Department of Earth Sciences,

Durham University using protocols established by Ottley et al. (2003) (6). 0.100 ± 0.001 g of powder was digested in a 4 ml 40% HF e 1 ml 69% HNO_3 solution for 48h before evaporating to dryness and redissolving in HNO_3 acid, the resulting solution being 3.5% HNO_3. This solution then had a Re and Rh internal standard added to compensate for possible calibration drift, matrix suppression and dilution errors. The analysis measured for 42 elements (Supplementary table S4). The major elements, analysed by ICP-MS as weight percentage oxide, include Al_2O_3, Fe_2O_3, MgO, CaO, Na_2O, K_2O, TiO_2, and P_2O_5. The minor and trace elements analysed by ICP-MS as parts per million (ppm) include Co, Cr, Cu, Ni, Mn, Sc, Sr, V, Zn, Rb, Y, Zr, Nb, Cs, Ba, La, Ce, Pr, Nd, Sm, Eu, Gd, Tb, Dy, Ho, Er, Tm, Yb, Lu, Hf, Ta, Pb, Th, and U. Calibration was achieved via the use of in-house standards and international reference materials (W-2, BHVO-1 and AGV-1 standards) as well as a blank and standard sample being run every 10 samples to ascertain instrument calibration stability. A Log-ratio transform (base 10) was applied to the raw data (7) before processing.

X-Ray Diffraction

XRD analysis was conducted on 25 ceramic samples from different fabric groups to strengthen petrographic analysis, gain further insights into the mineralogical profile of different fabric groups and examine the firing temperatures of the hand-made and wheel-made pottery (8). XRD patterns and estimates of firing temperature of samples are provided in Supplementary file S5. Sample preparation and analysis took place at the DARC, utilising 1.250 g of powdered sample.

XRD analysis took place on a Panalytical Aeris XRD with a CuKα1 emitter. Measurements were taken from 5 to 70° 2Θ at a step size of approximately 0.0054° at 39.5 s per step. The powdered sample was placed in a circular sample holder with a diameter of 32 mm and a depth of 3 mm. A nickel-beta filter was used on the incident side, along with 0.04 rad soller-slits inserted on the both the incident and detector side of the beam. The analytical configuration also included a ¼° divergence slit, a 20 mm beam mask, a beam knife in the 'hi' position, and a 9 mm antiscatter slit. The total time for the analysis of each sample was 20 minutes. The analysis of the results was completed using the 'HighScore' proprietary software package by Panalytical and the International Centre for Diffraction 'Minerals' Database (ICDD).

1. Whitbread IK. A proposal for the systematic description of thin sections towards the study of ancient ceramic technology. In: Maniatis Y, editor. Archaeometry: proceedings of the 25th international symposium. Amsterdam: Elsevier Science Publishers B.V.; 1989. p. 127-38.
2. Whitbread IK. Greek transport amphorae: a petrological and archaeological study. Athens: British School at Athens; 1995.
3. Quinn PS. Ceramic petrography: The interpretation of archaeological pottery related artefacts in thin section. Oxford: Archaeopress; 2013.

4. Schneider CA, Rasband WS, Eliceiri KW. NIH Image to ImageJ: 25 years of image analysis. Nature methods. 2012;9(7):671-5.
5. Degryse P, Braekmans D. Elemental and isotopic analysis of ancient ceramics and glass. Treatise on Geochemistry. 2014;14:191-207.
6. Ottley C, Pearson G, Irvine GJ. A routine method for the dissolution of geological samples for the analysis of REE and trace elements via ICP-MS. In: Holland J, Tanner S, editors. Plasma source mass spectrometry: applications and emerging technologies. Cambridge: Royal Society of Chemistry; 2003. p. 221-30.
7. Papageorgiou I. Ceramic investigation: how to perform statistical analyses. Archaeological and Anthropological Sciences. 2020;12(9):1-19.
8. Maggetti M, Neururer C, Ramseyer D. Temperature evolution inside a pot during experimental surface (bonfire) firing. Applied Clay Science. 2011;53(3):500-8.
