# Supplementary material for: Capturing technological crossovers between clay crafts: An archaeometric perspective on the emergence of workshop production in Late Iron Age northern Spain
Source: PLoS One. 2023 May 5;18(5):e0283343. doi: 10.1371/journal.pone.0283343 (PMC10162541; doi:10.1371/journal.pone.0283343)
Supplement: S2 File — (PDF) [file pone.0283343.s002.pdf]

Appendix 2 – Ceramic fabric descriptions

Fabric descriptions of Late Iron Age ceramics from El Cerrito (Cella, Teruel)

Table S2.1. Fabrics and associated samples.

Type	Fabric	Description	Samples
Wheel-made	1.1	Calcareous clay (*n* = 1)	C1.5
Wheel-made	1.2	Clay with quartz and micrite (*n* = 1)	C9.1
Wheel-made	2.1	Fine micrite and quartzite (non-calcareous) (*n* = 14)	C1.1, C1.2, C1.3, C1.4, C1.10, C1.11, C1.12, C1.14, C1.15, C6.1, C6.2, C6.3, C6.5, C7.1
Wheel-made	2.2	Fine quartz (non-calcareous) (*n* = 2)	C1.8, C1.9
Wheel-made	2.3	Sand temper (*n* = 1)	C1.7
Wheel-made	3.1	Quartz-tempered in micrite-rich matrix (*n* = 5)	C1.6, C6.6, C8.1, C1.13, C7.2
Wheel-made	3.2	Oolitic limestone and ferruginous inclusions (*n* = 1)	C6.4
Hand-made	4.1	Shale tempered fabric (*n* = 11)	C3.1, C4.1, C4.2, C4.3, C10.2, C11.1, C11.2, C12.1, C13.4, C13.5, C13.8
Hand-made	4.2	Shale and grog tempered fabric (*n* = 9)	C10.1, C12.2, C12.3, C13.6, C13.7, C14.1, C14.2, C14.3, CP2.1, CP2.2
Hand-made	5	Grog and calcite temper (*n* = 3)	C13.1, C13.2, C13.3
Adobe from combustion chamber	6.1	Calcareous building material (*n* = 1)	CBM10
Hand-made ceramic building material used in combustion chamber	6.2	Calcareous clay with oolitic limestone (*n* = 3)	CBM1, CBM2, CMB3, CBM4

Table S2.2. Microphotographs of El Cerrito fabrics.

**Sample nr.**	**Petro Fabric**	**Image** (not to scale)	**Micro-photograph** XPL, field of view: 3.0mm	**Micro-photograph** PPL, field of view: 3.0mm
C1.1	2.1	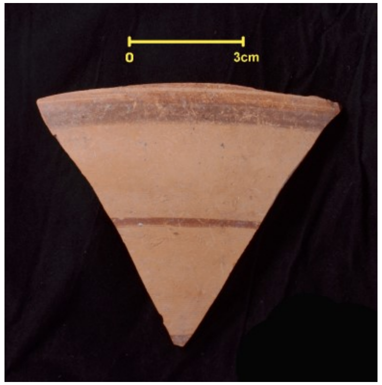	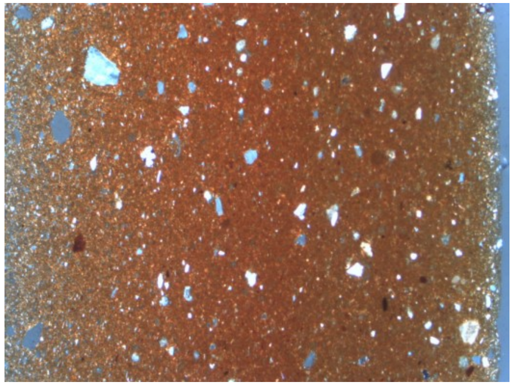	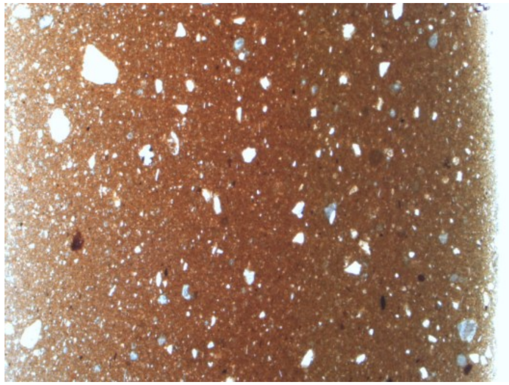
C1.2	2.1	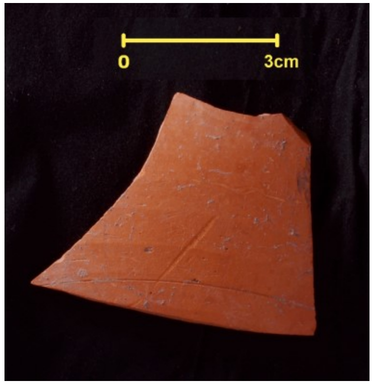	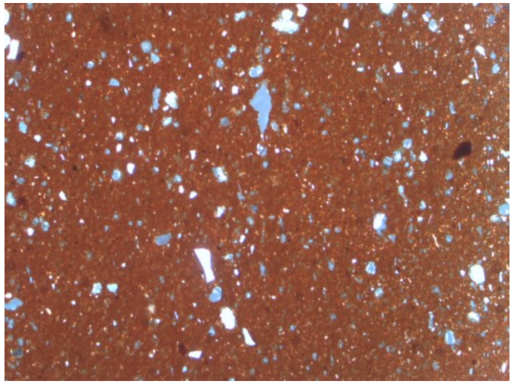	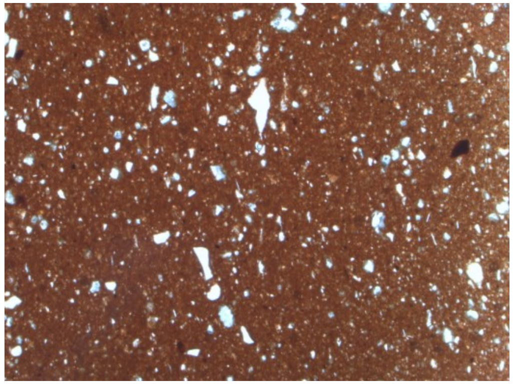

C1.3	2.1	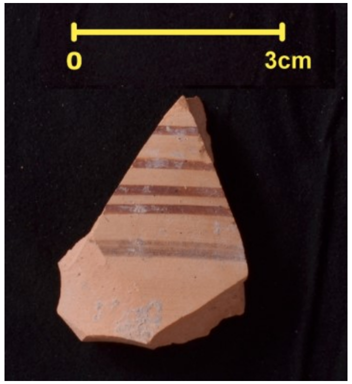	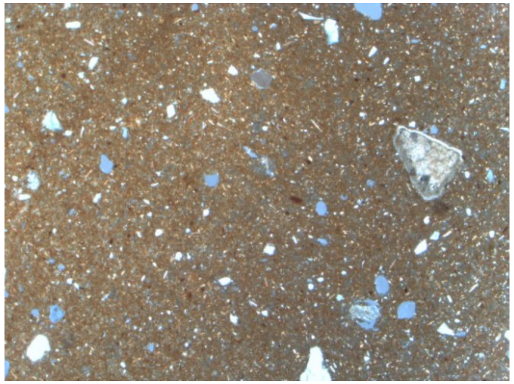	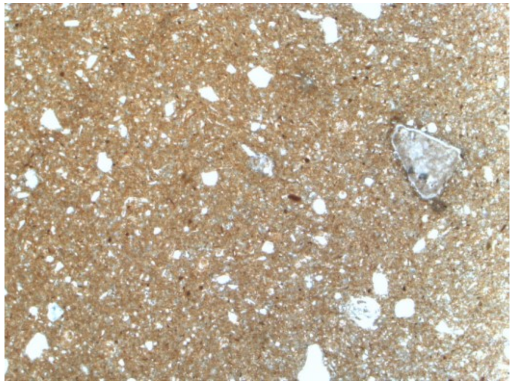
C1.4	2.1	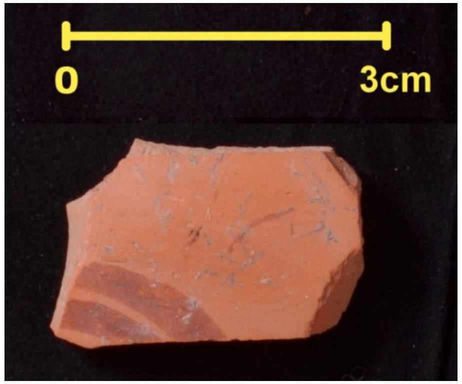	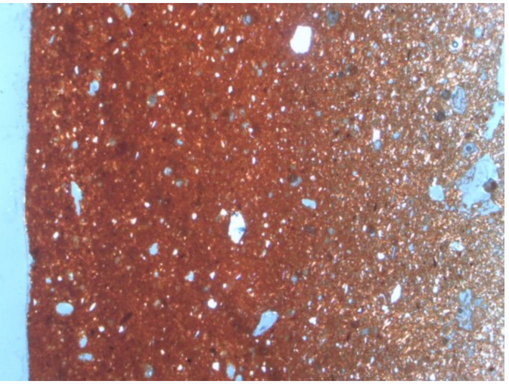	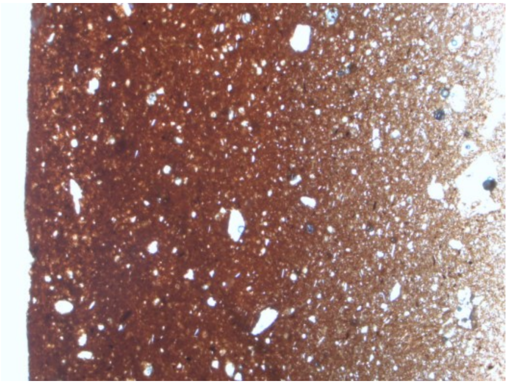
C1.5	1.1	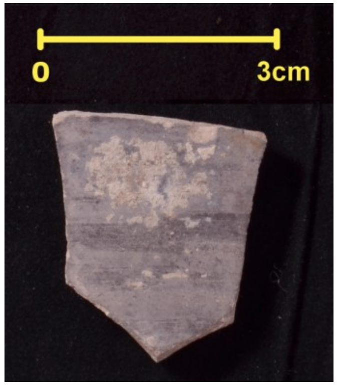	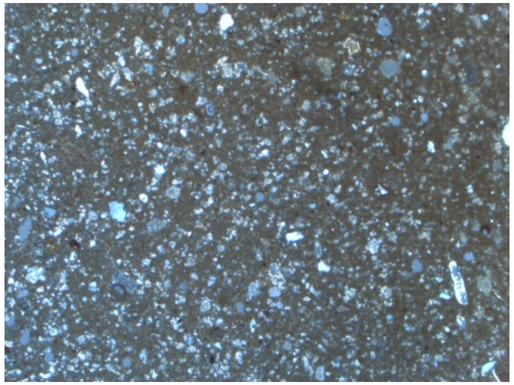	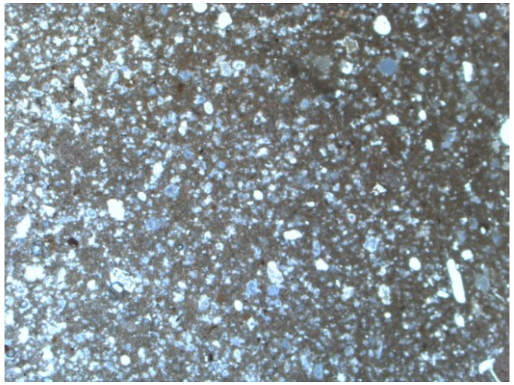

C1.6	3.1	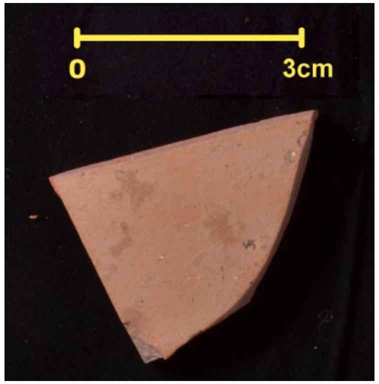	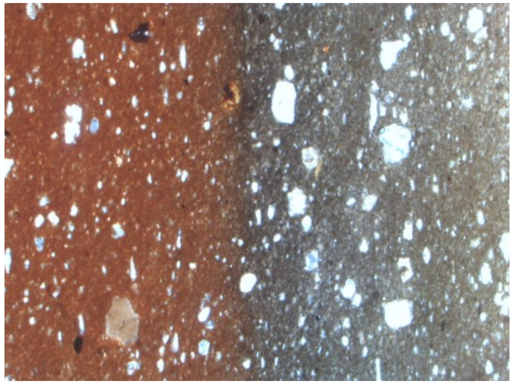	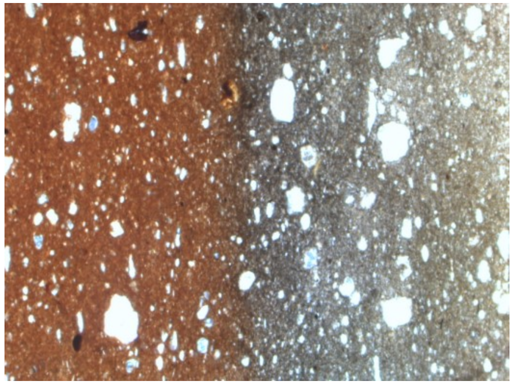
C1.7	2.3	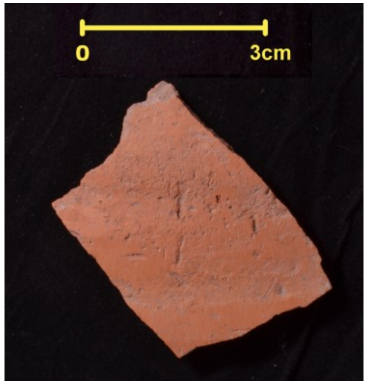	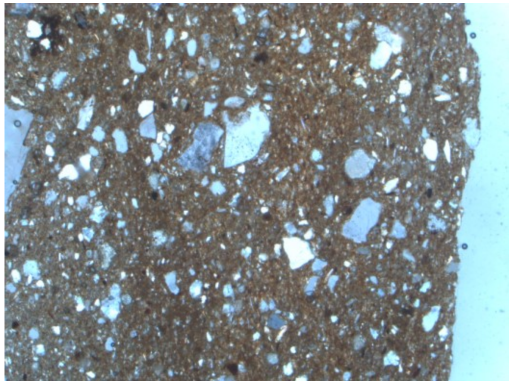	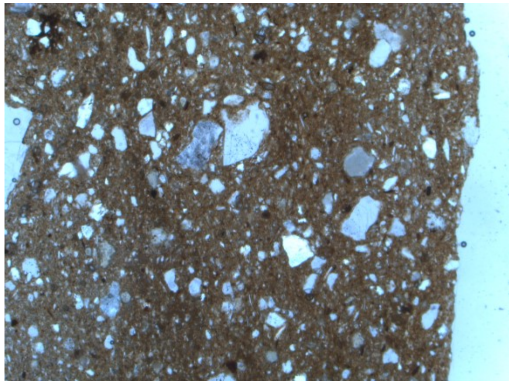
C1.8	2.2	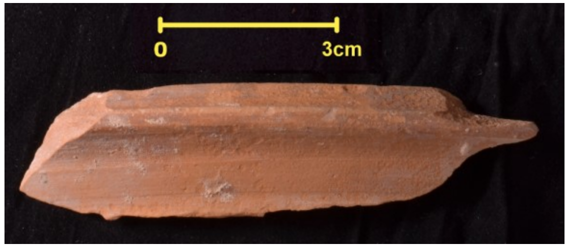	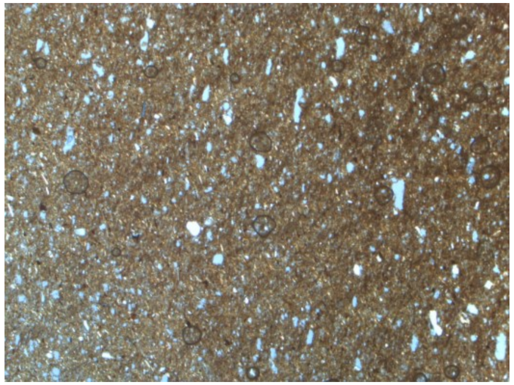	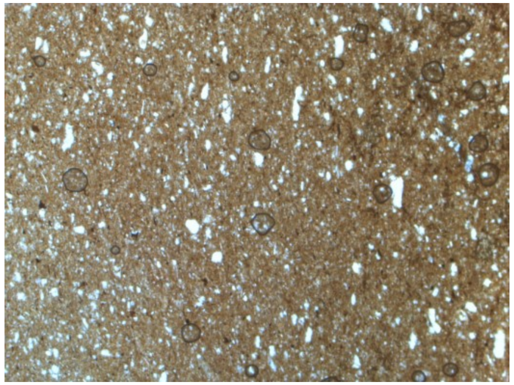

C1.9	2.2	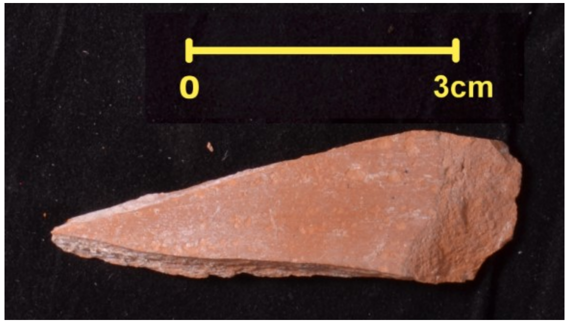	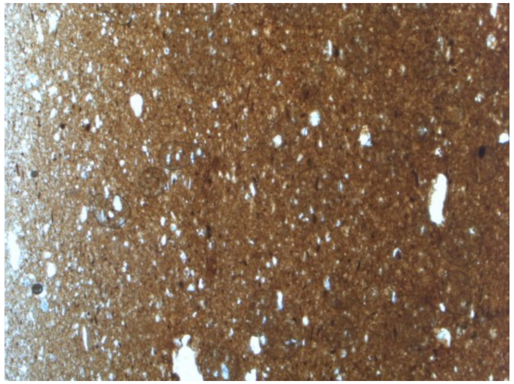	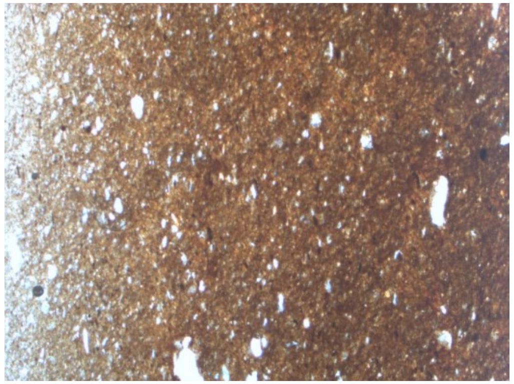
C1.10	2.1	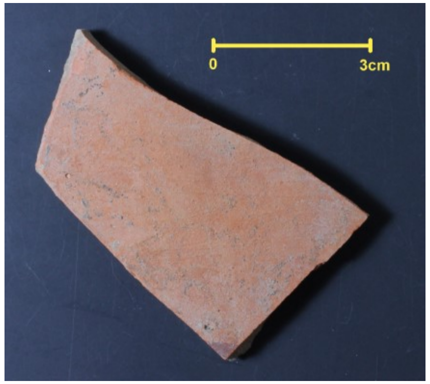	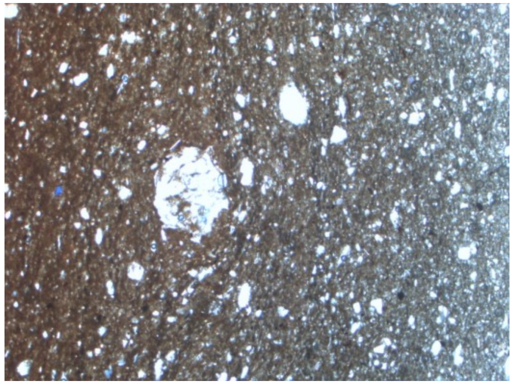	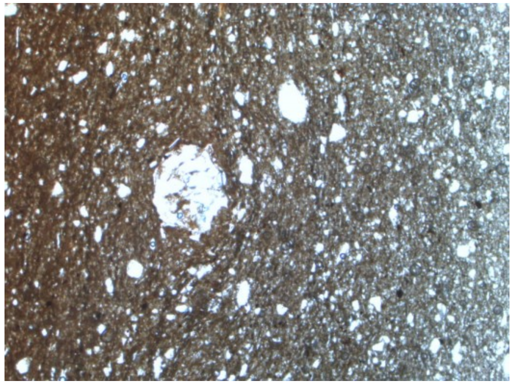
C1.11	2.1	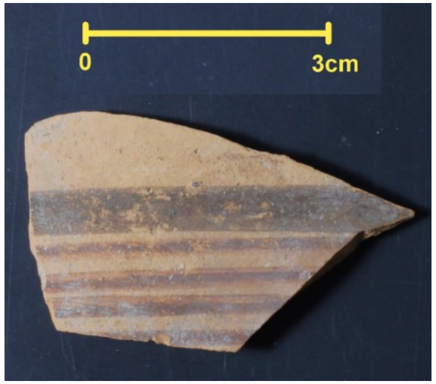	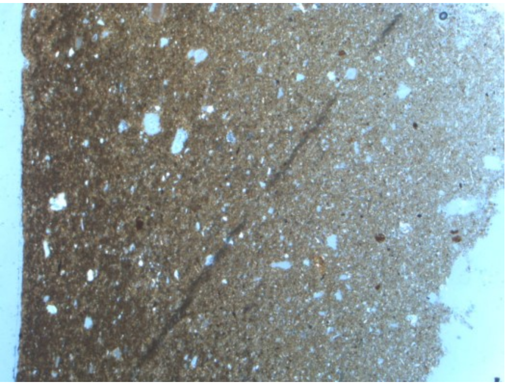	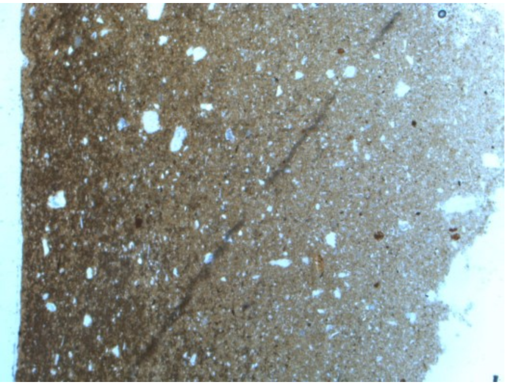

C1.12	2.1	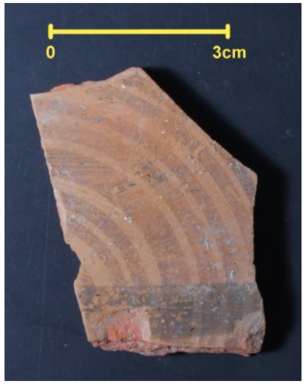	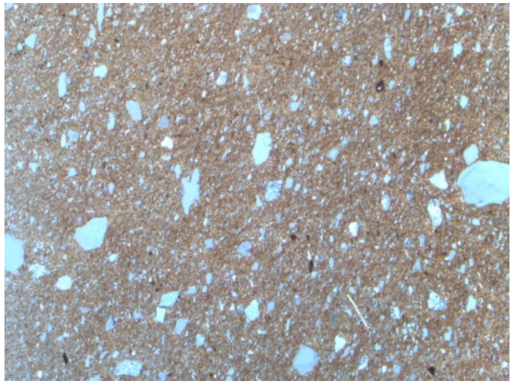	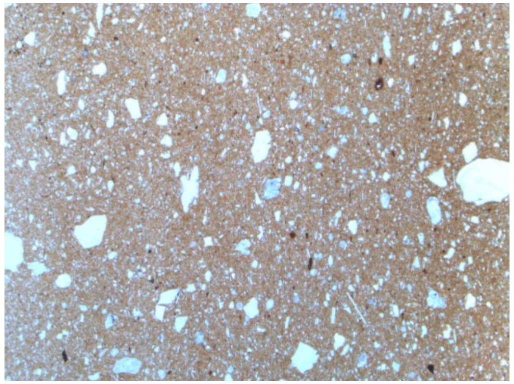
C1.13	3.1	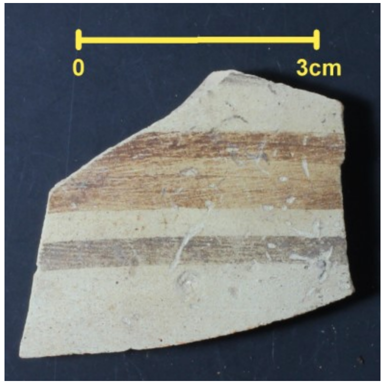	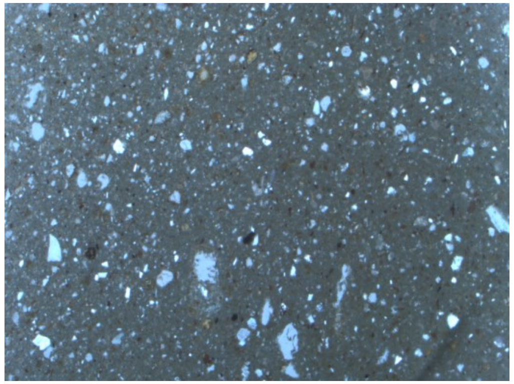	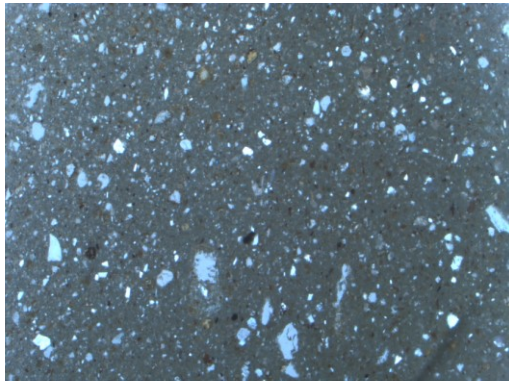
C1.14	2.1	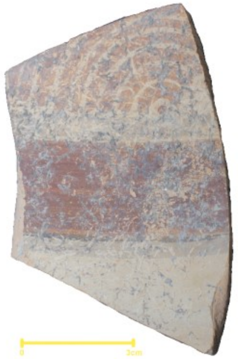	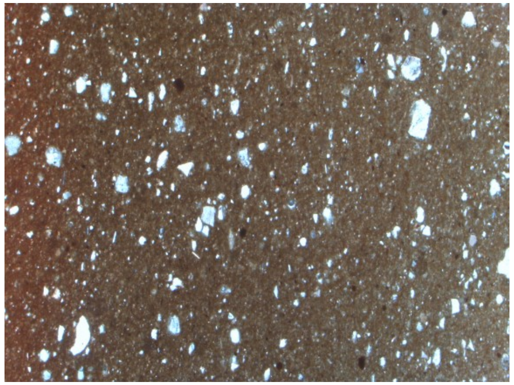	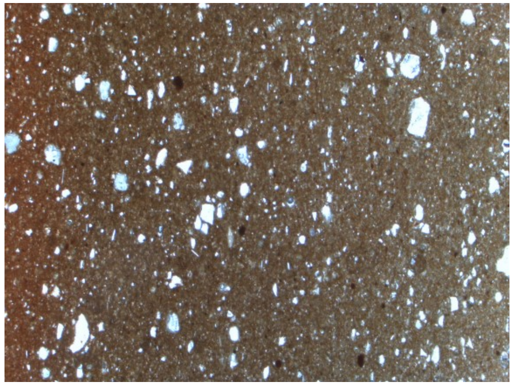

C1.15	2.1	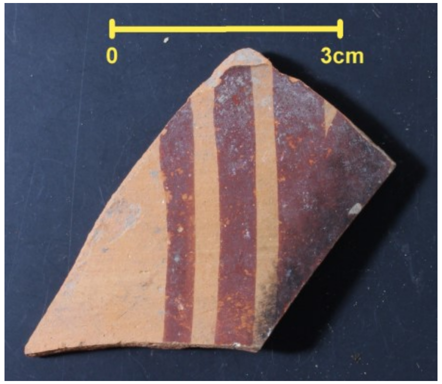	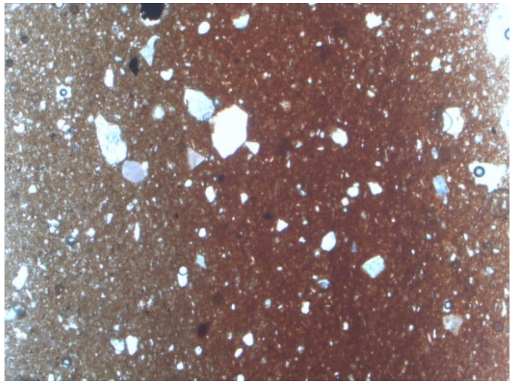	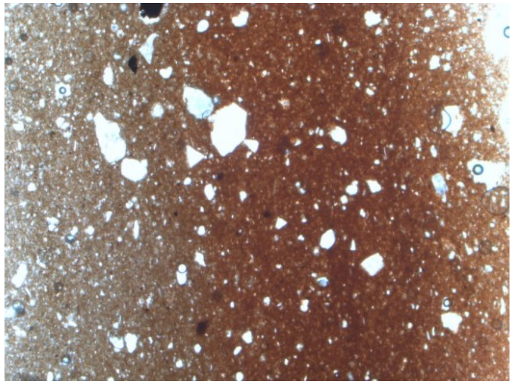
C6.1	2.1	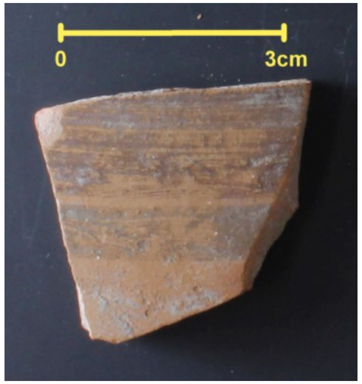	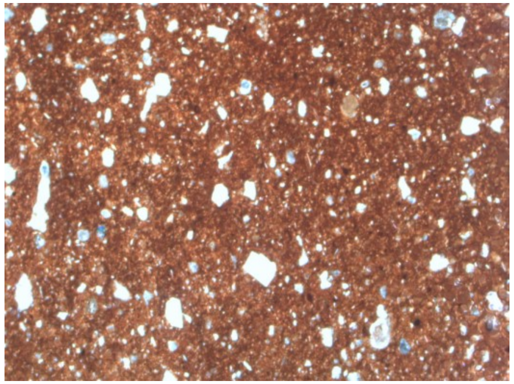	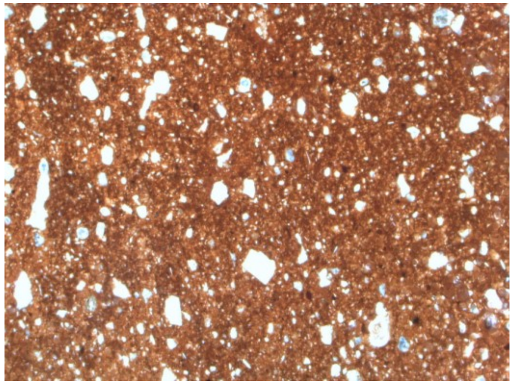
C6.2	2.1	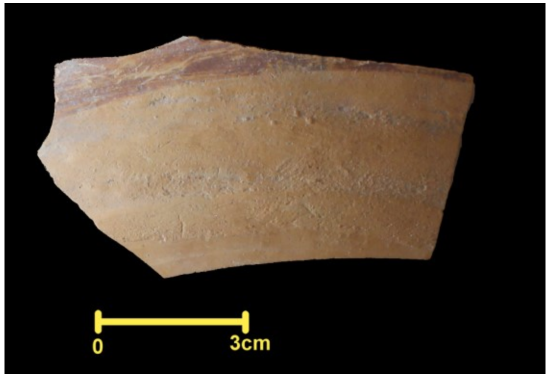	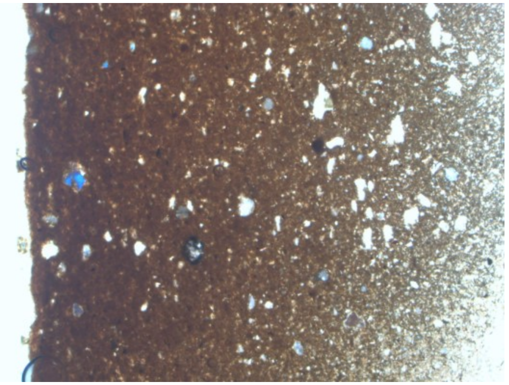	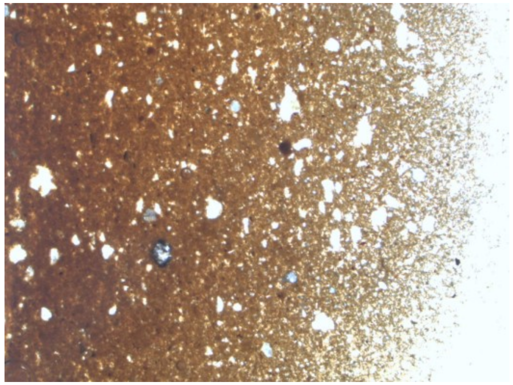

C6.3	2.1	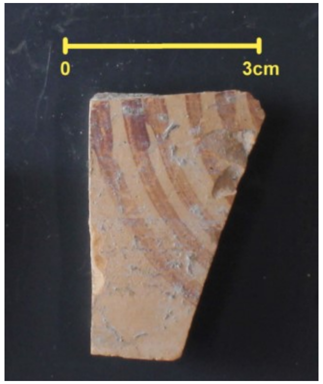	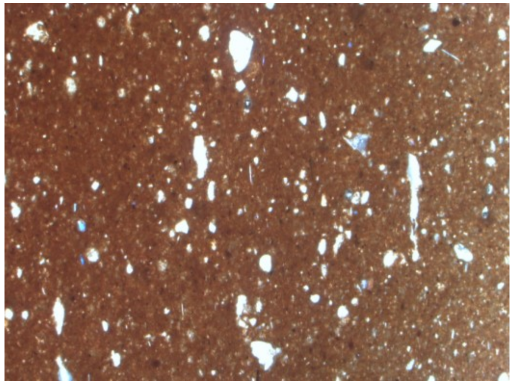	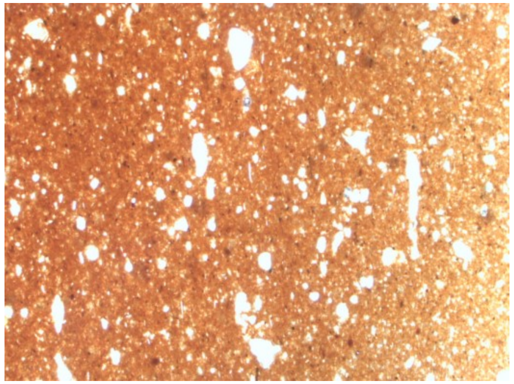
C6.4	3.2	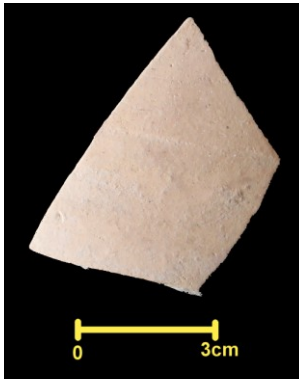	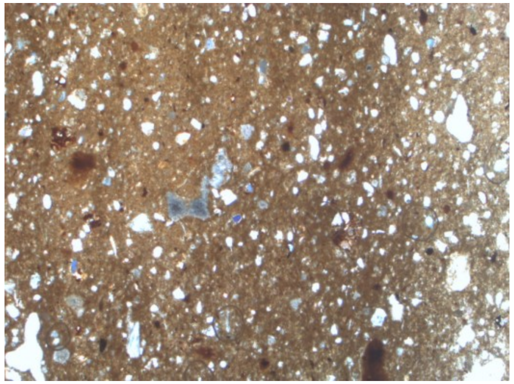	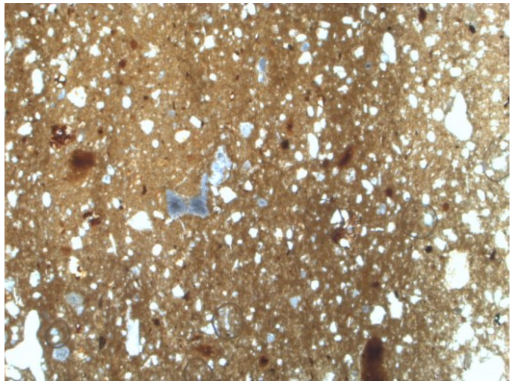
C6.5	2.1	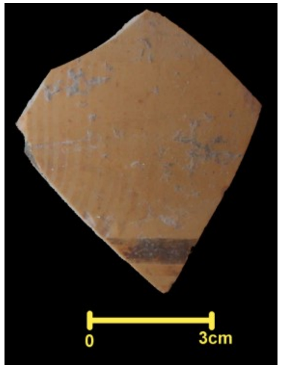	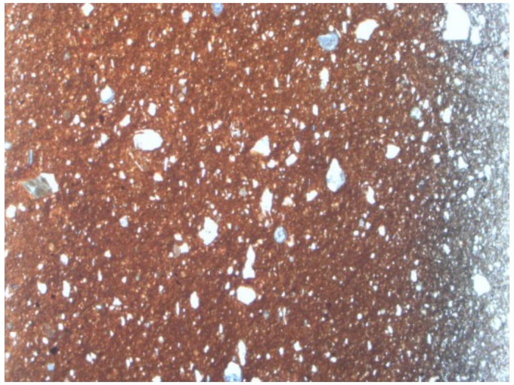	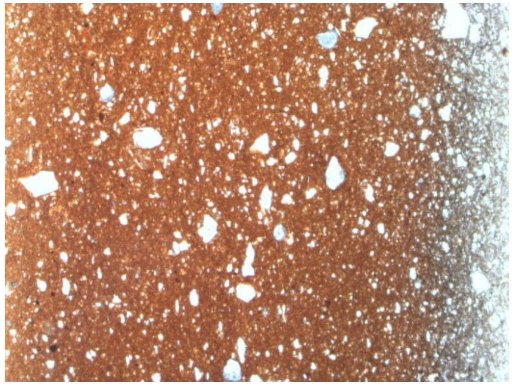

C6.6	3.1	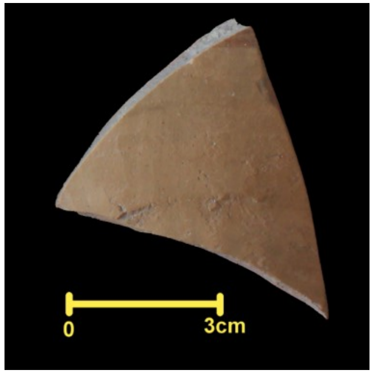	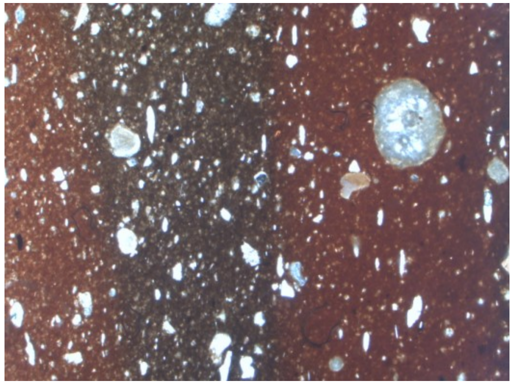	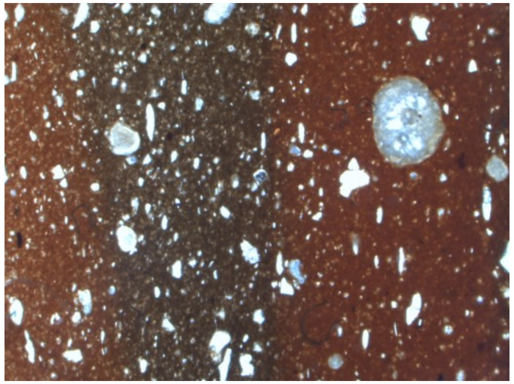
C7.1	2.1	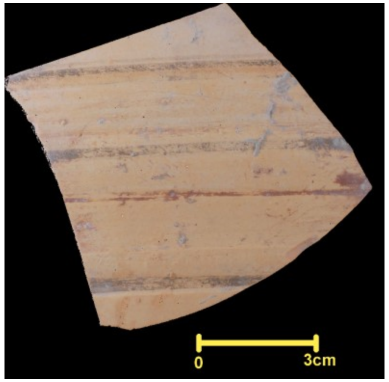	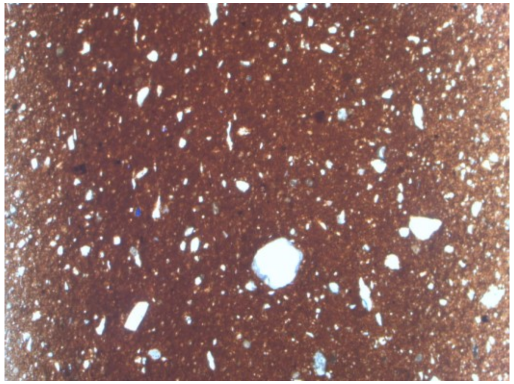	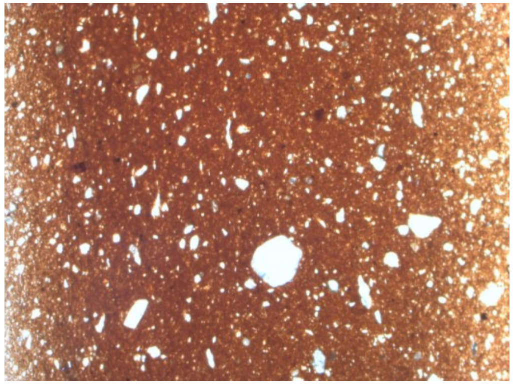
C7.2	2.1	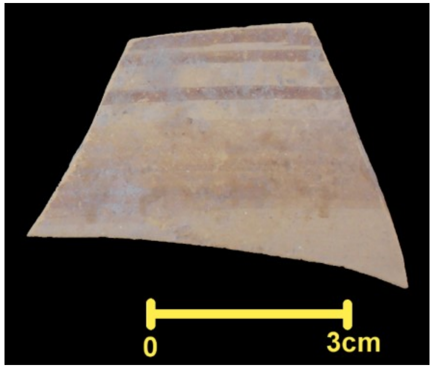	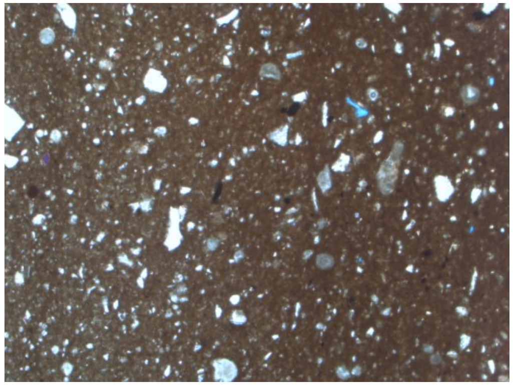	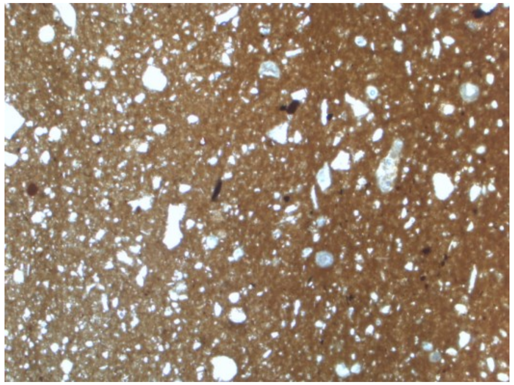

C8.1	3.1	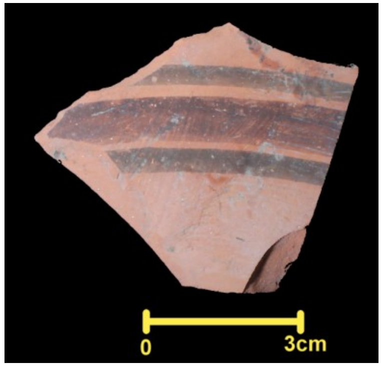	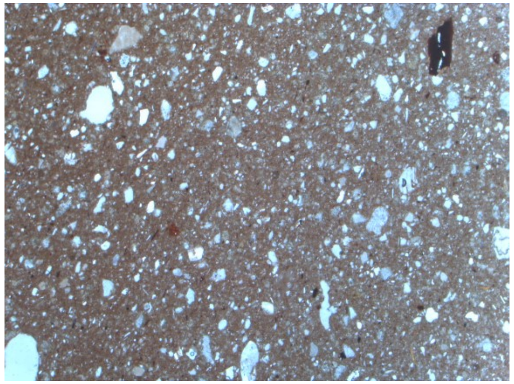	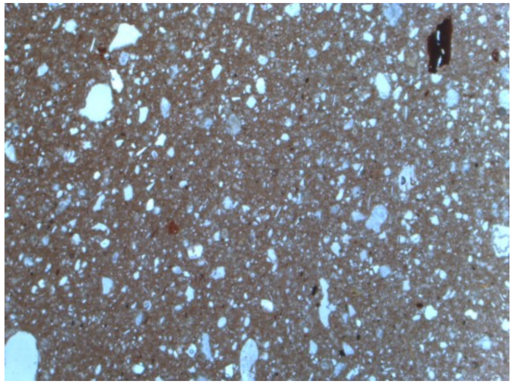
C9.1	1.2	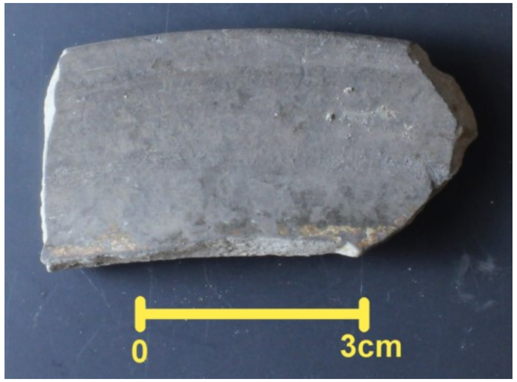	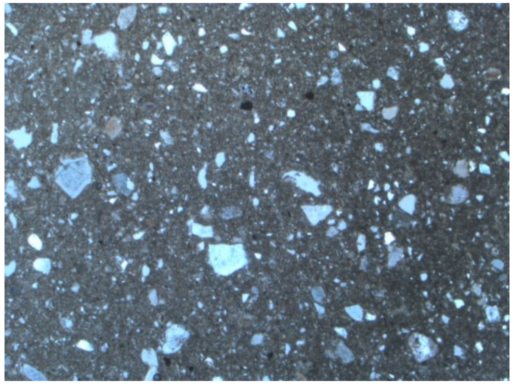	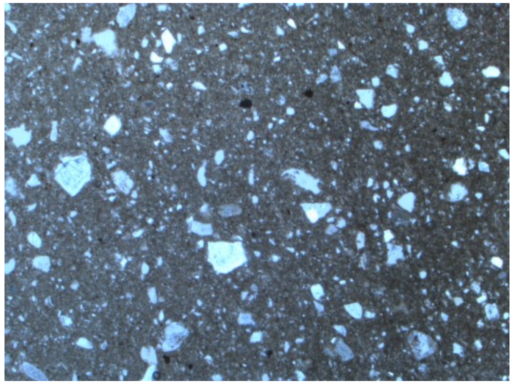
C3.1	4.1	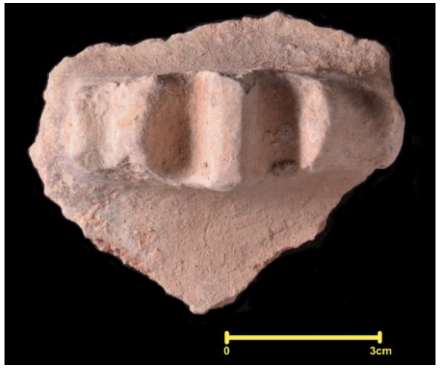	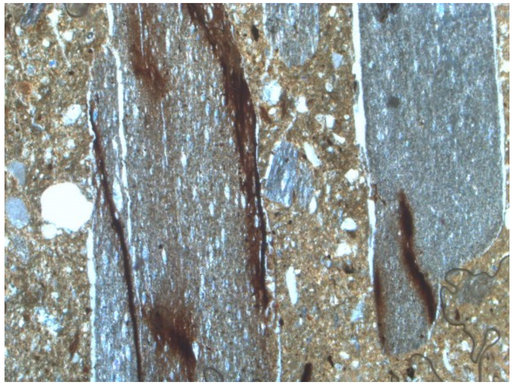	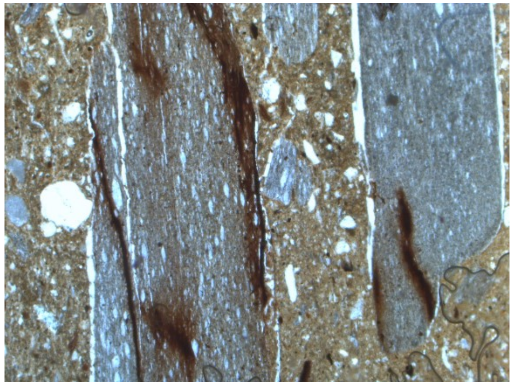

C4.1	4.1	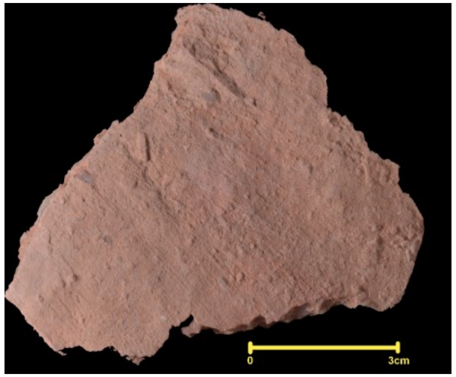	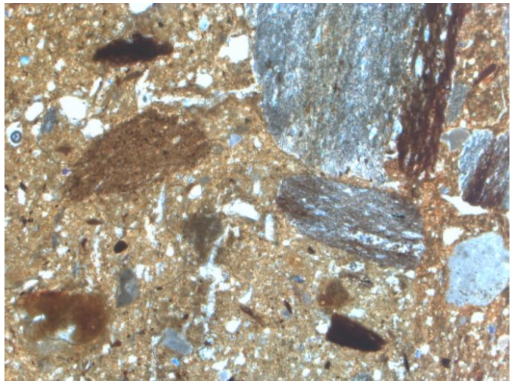	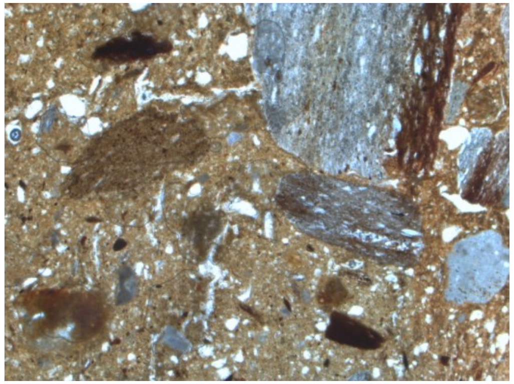
C4.2	4.1	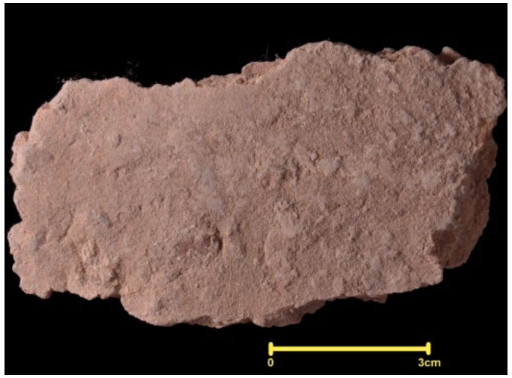	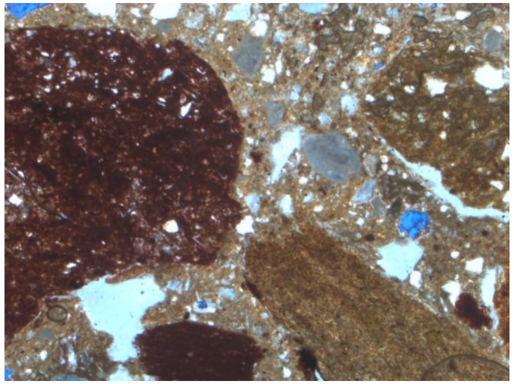	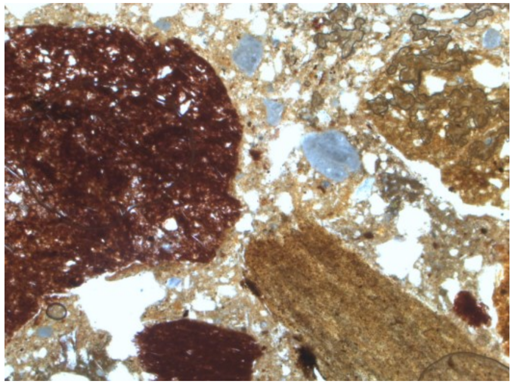
C4.3	4.1	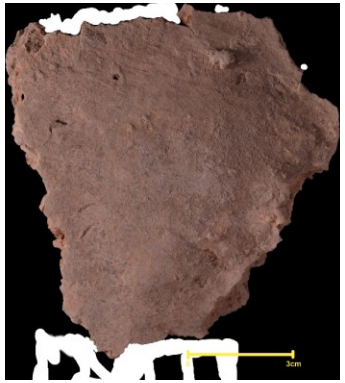	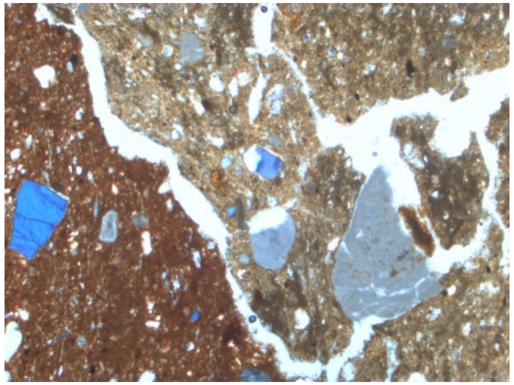	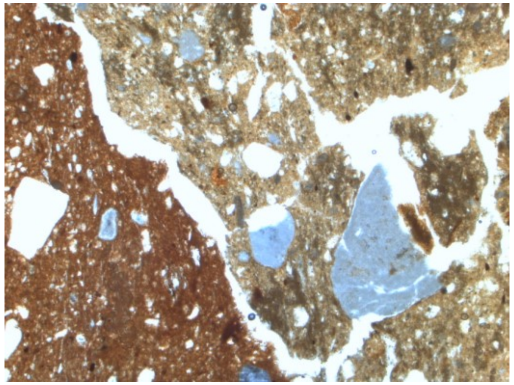

C10.1	4.2	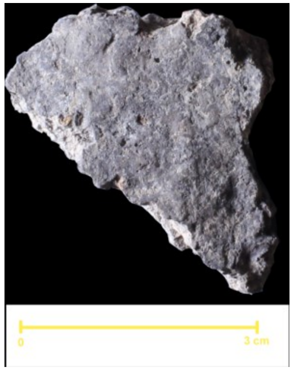	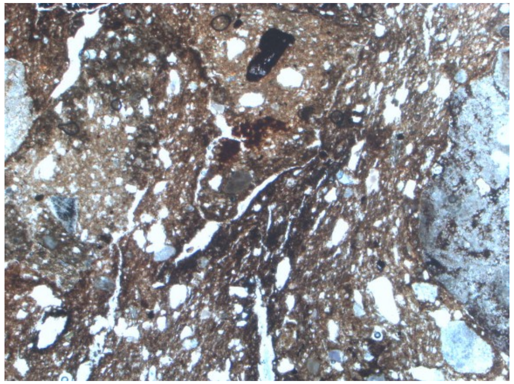	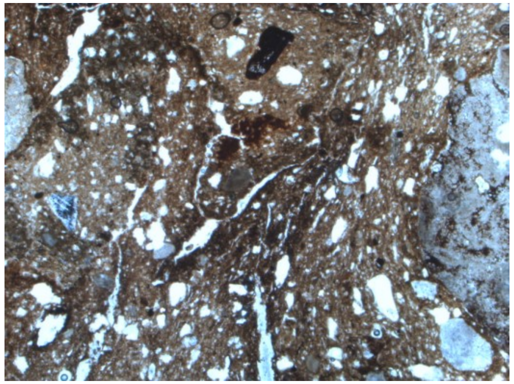
C10.2	4.1	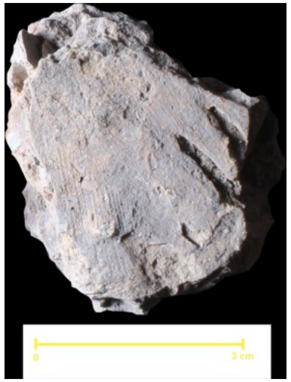	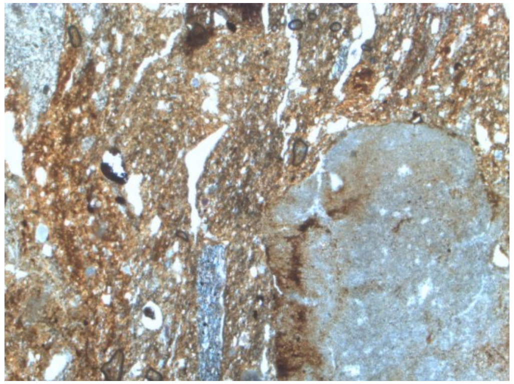	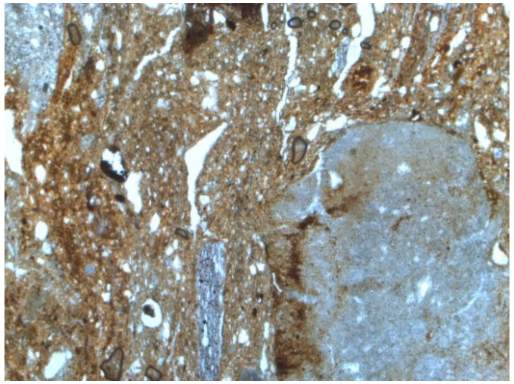
C11.1	4.1	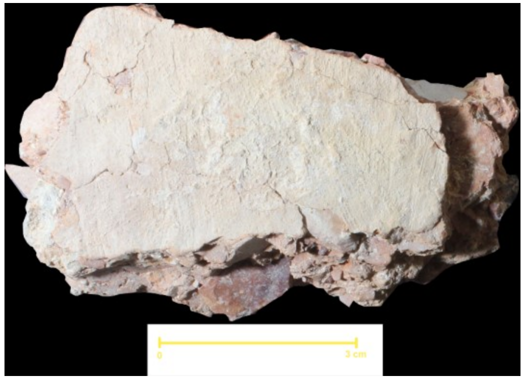	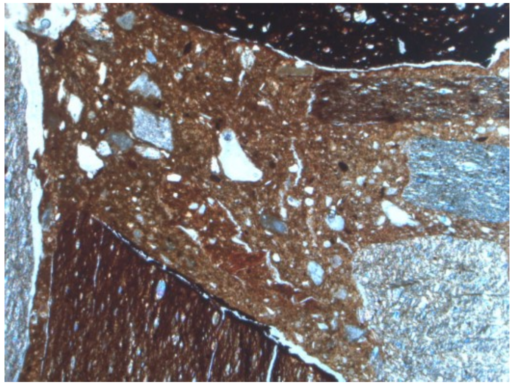	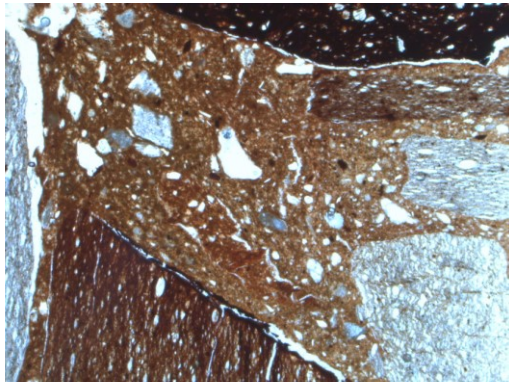

C11.2	4.1	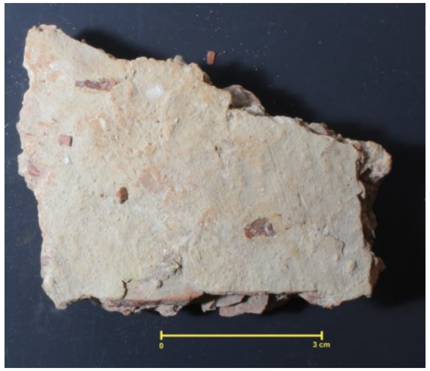	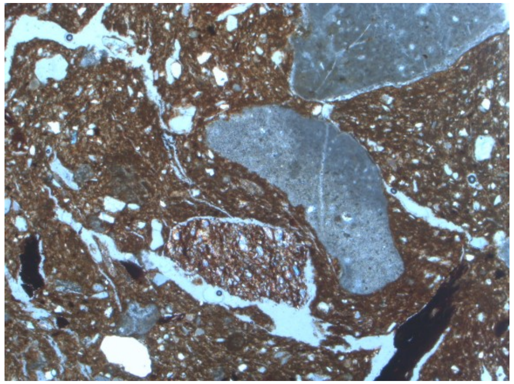	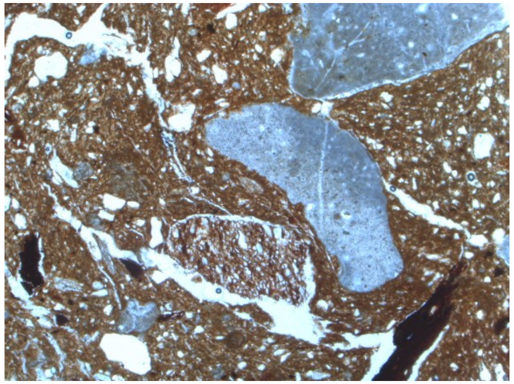
C12.1	4.1	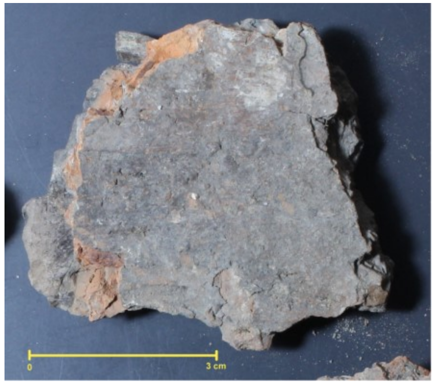	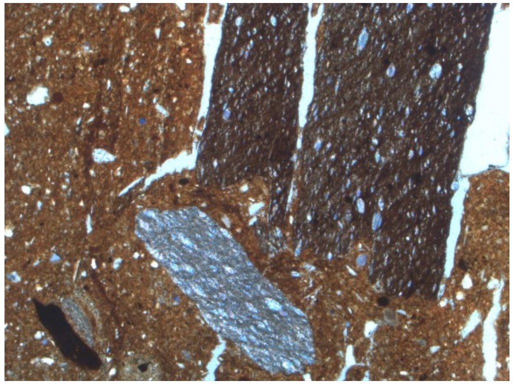	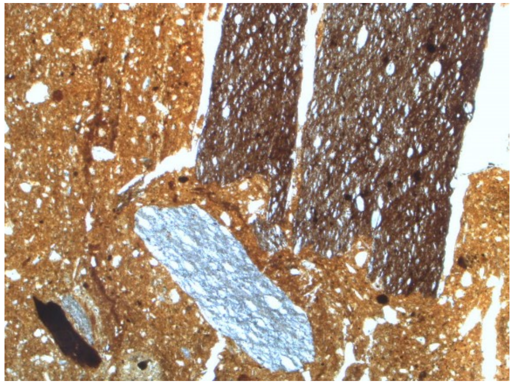
C12.2	4.2	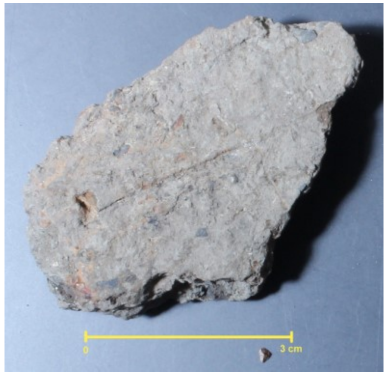	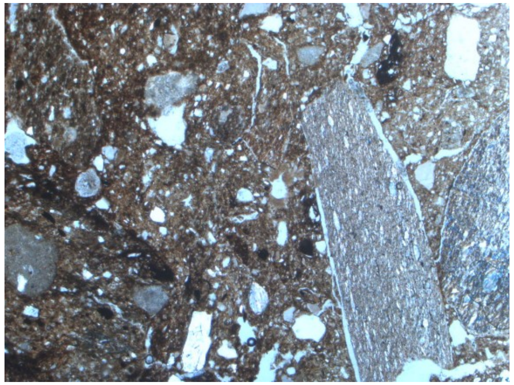	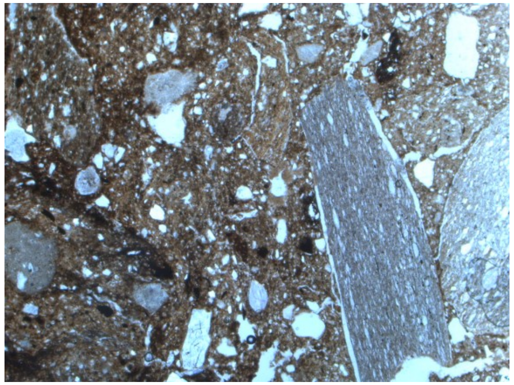

C12.3	4.2	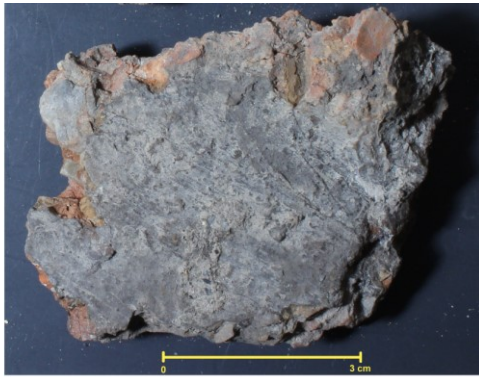	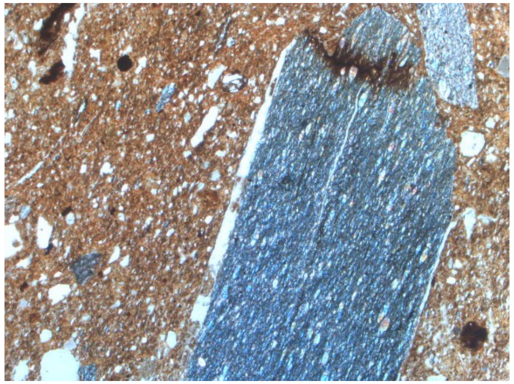	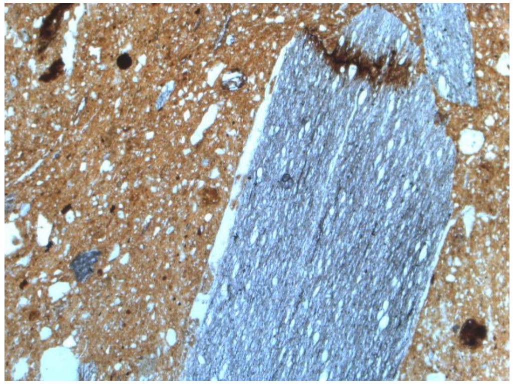
C13.1	5	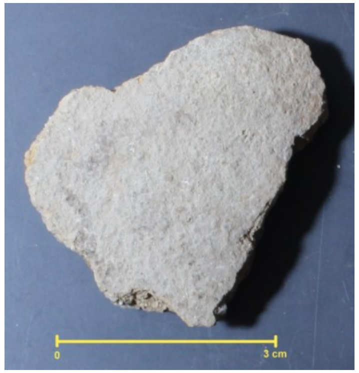	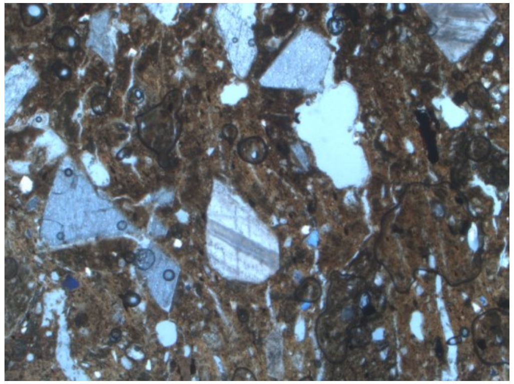	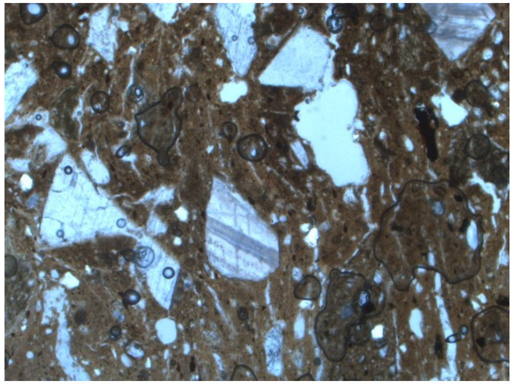
C13.2	5	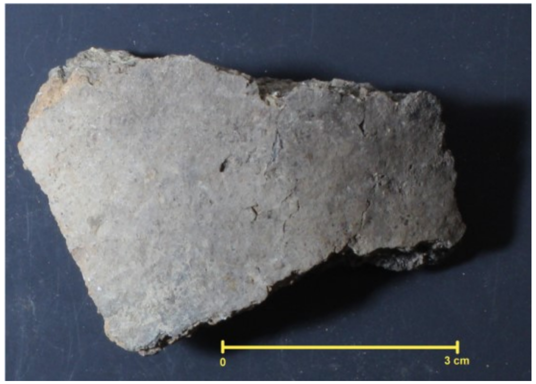	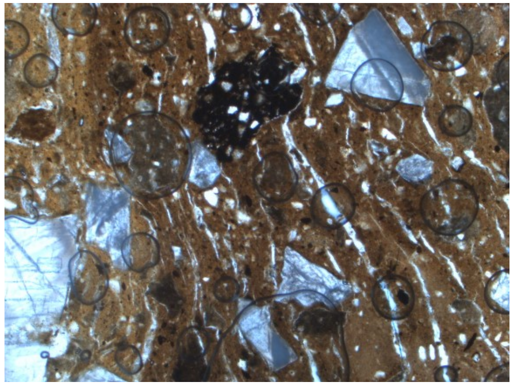	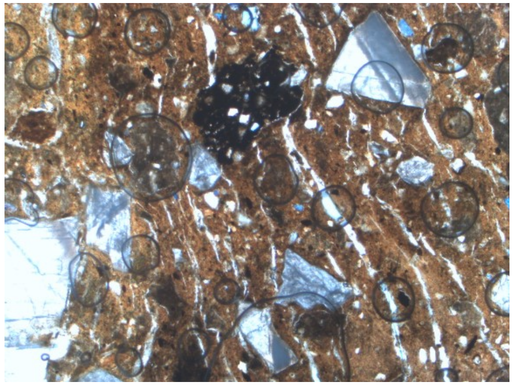

C13.3	5	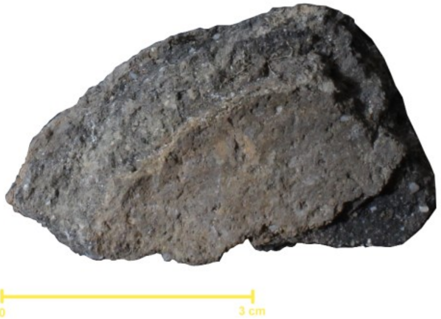	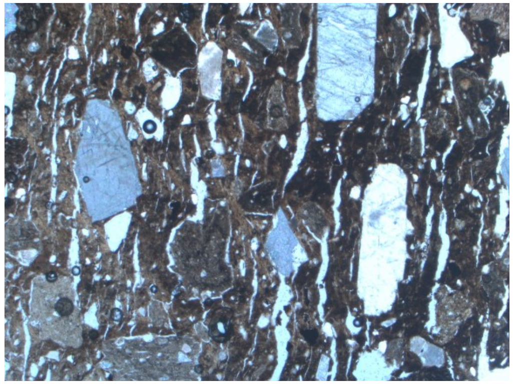	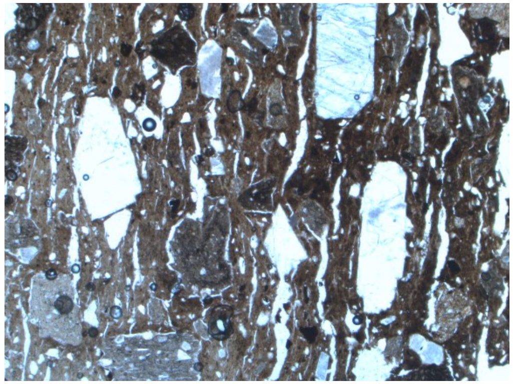
C13.4	4.1	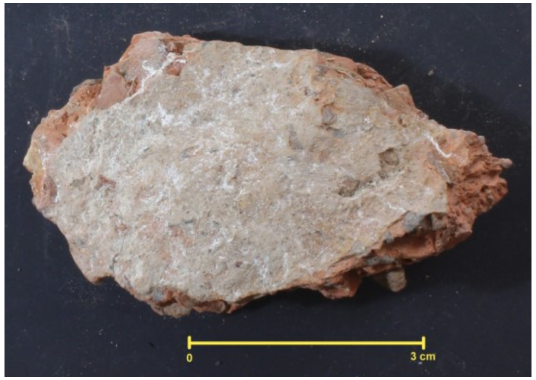	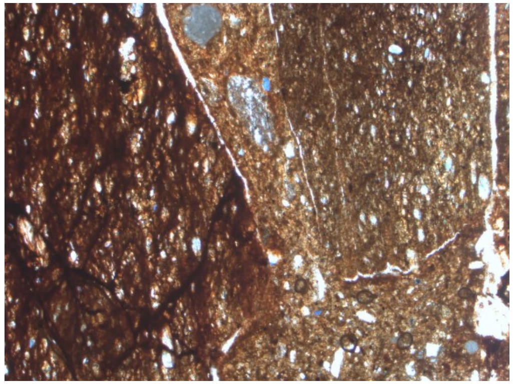	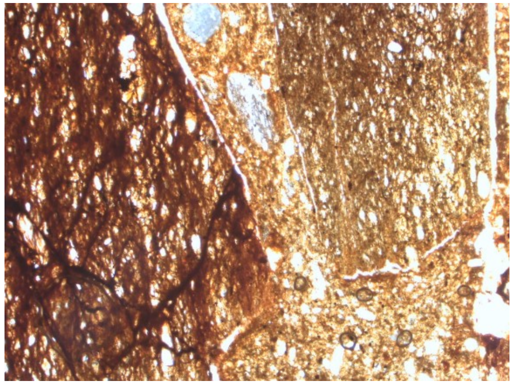
C13.5	4.1	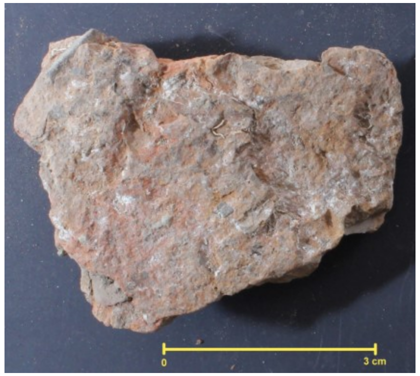	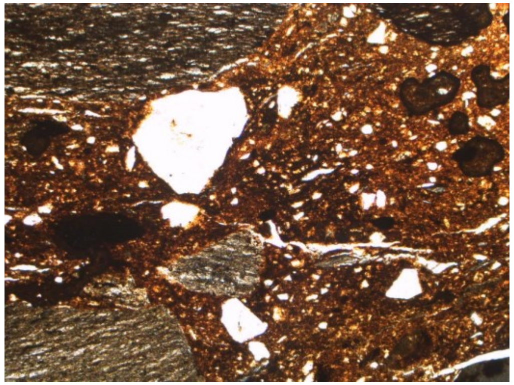	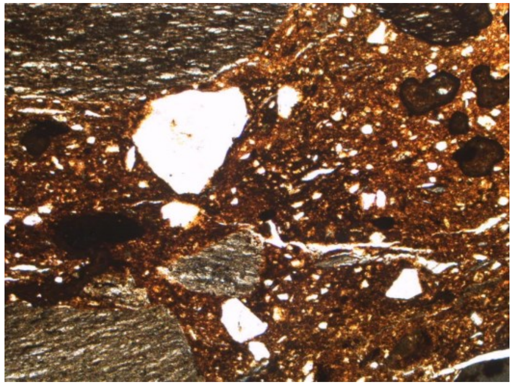

C13.6	4.2	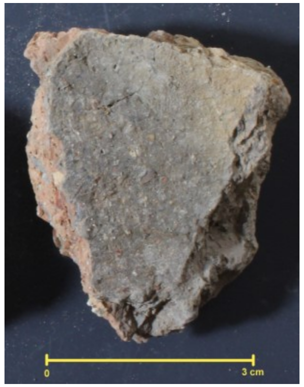	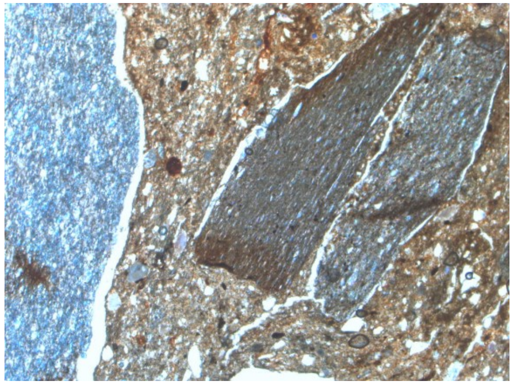	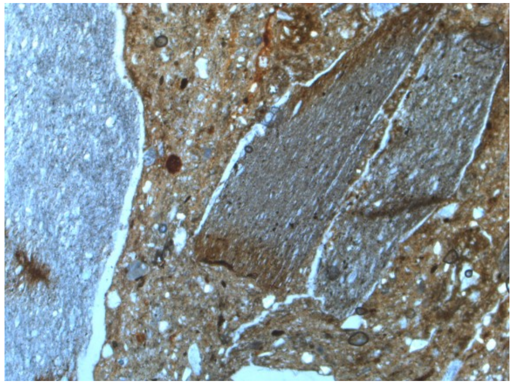
C13.7	4.2	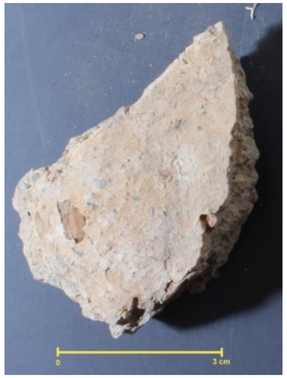	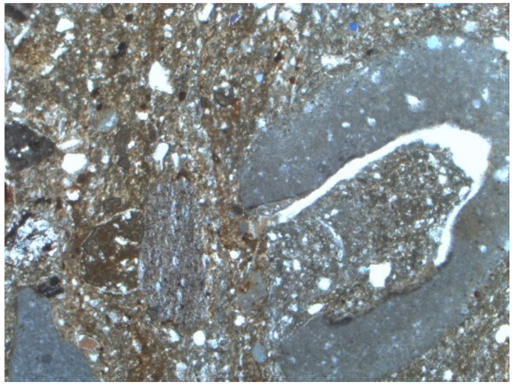	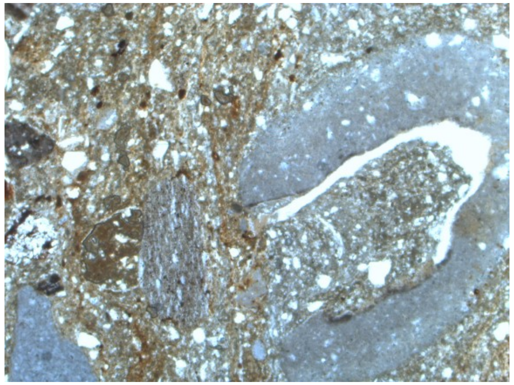
C13.8	4.1	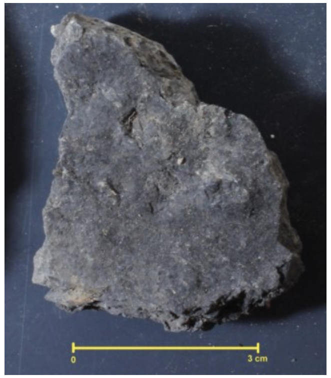	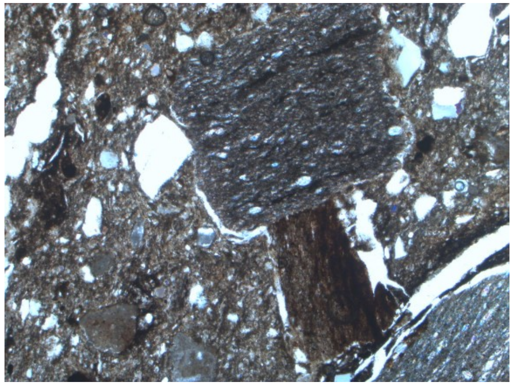	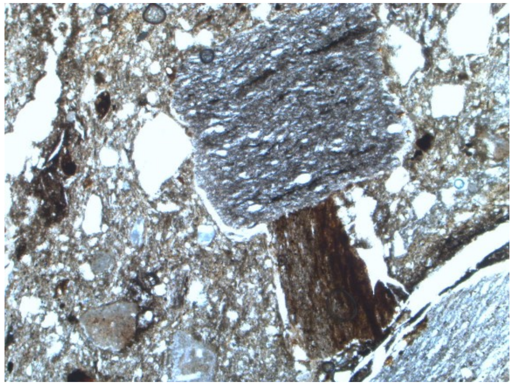

C14.1	4.2	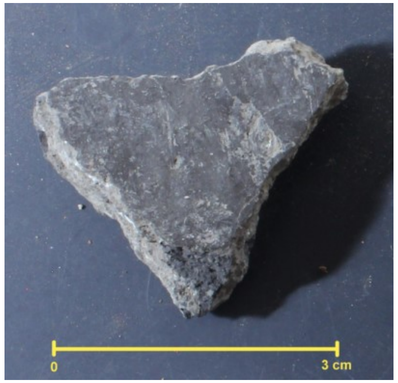	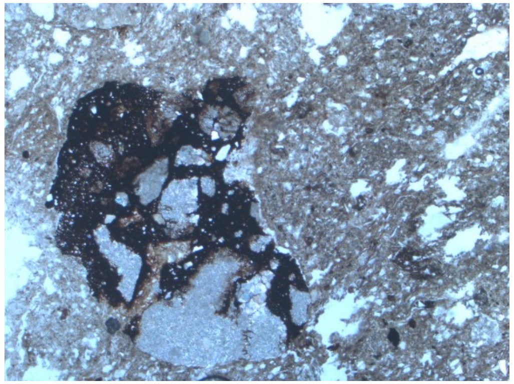	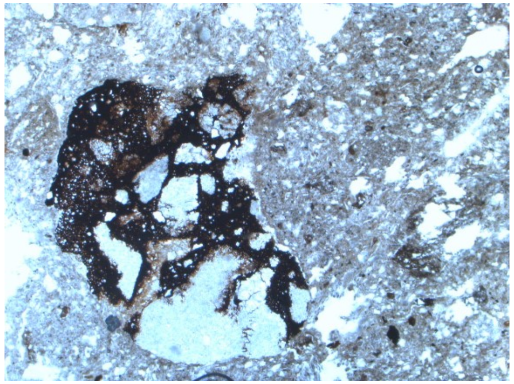
C14.2	4.2	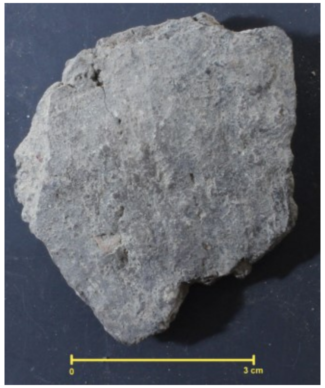	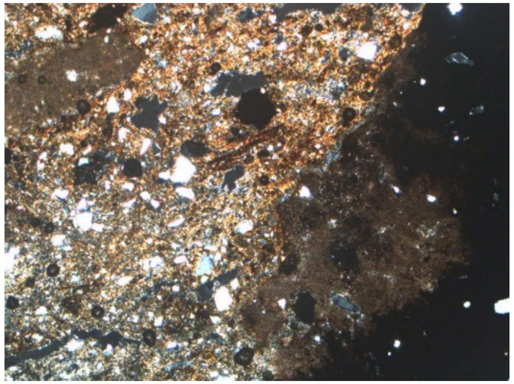	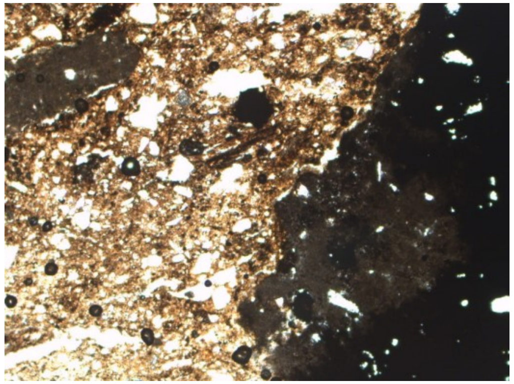
C14.3	4.2	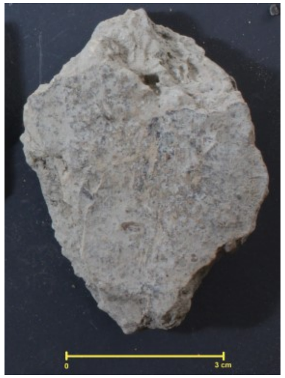	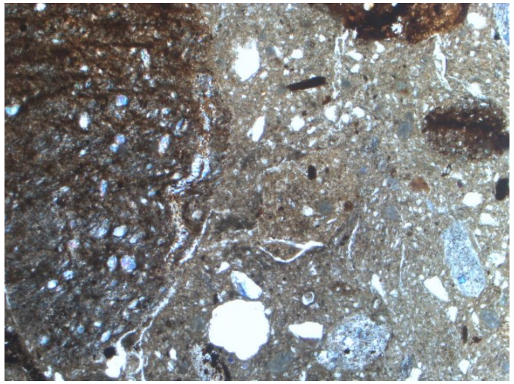	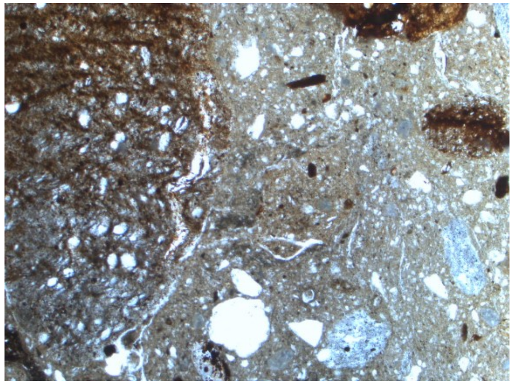

CBM1	6.2	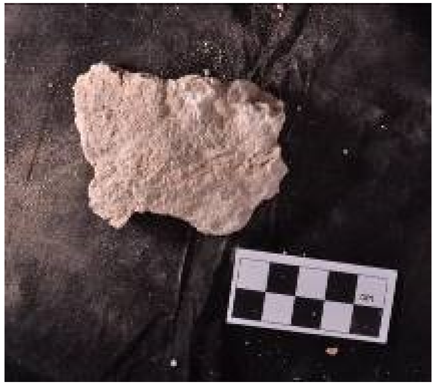	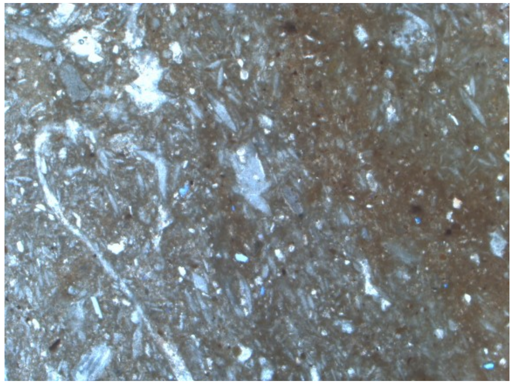	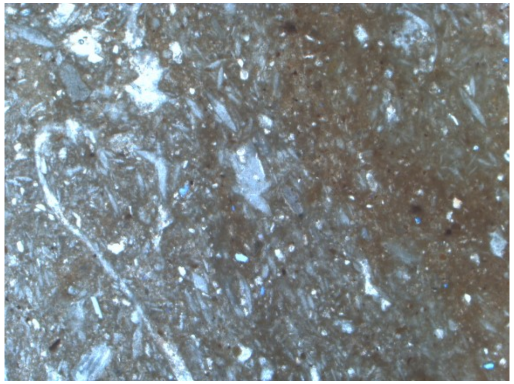
CBM2	6.2	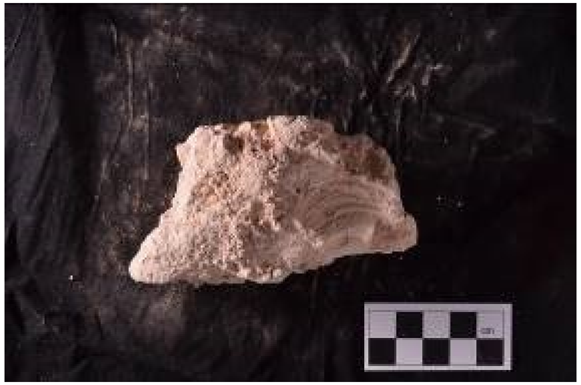	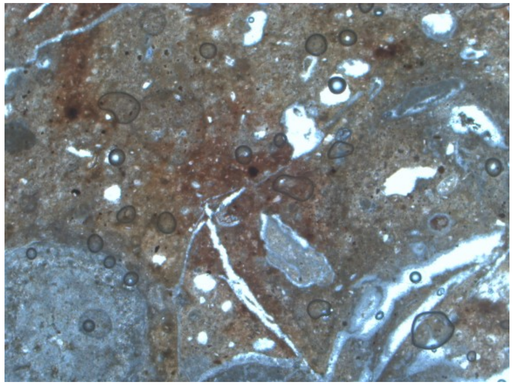	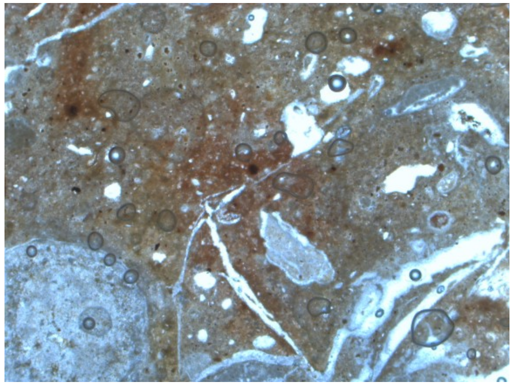
CBM3	6.2	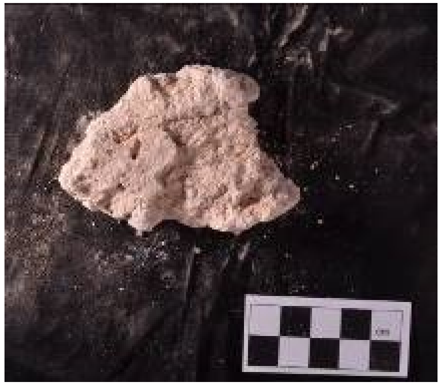	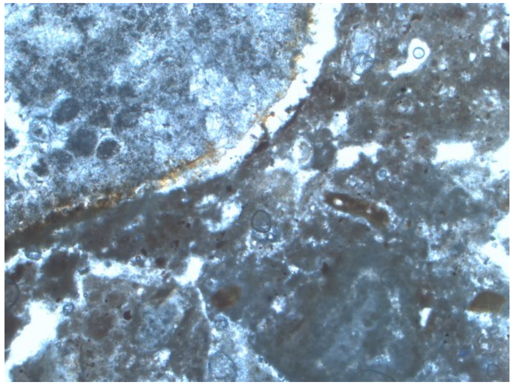	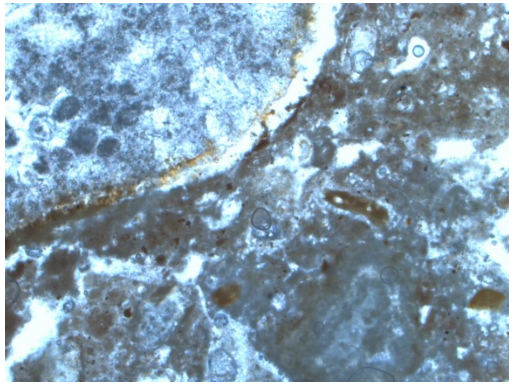

CBM10	6.1		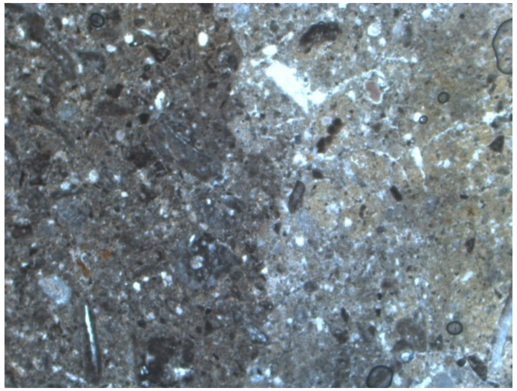	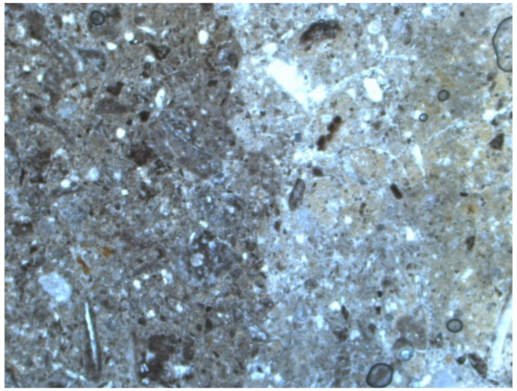
CP2.1	6.2	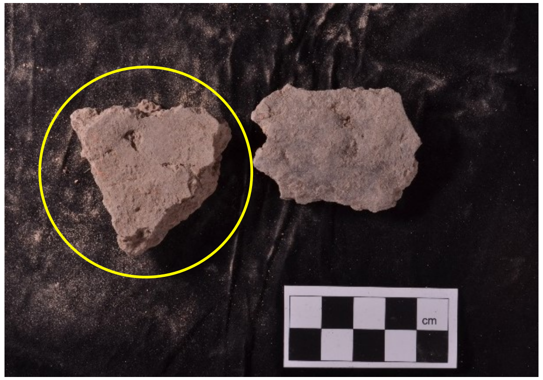	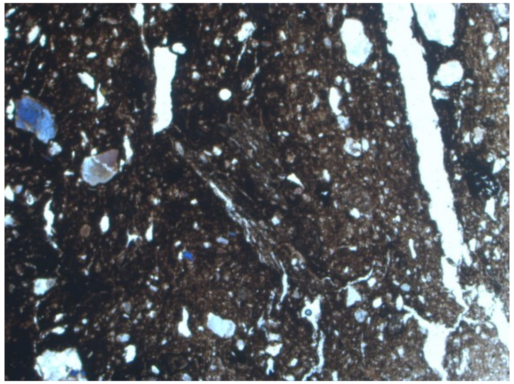	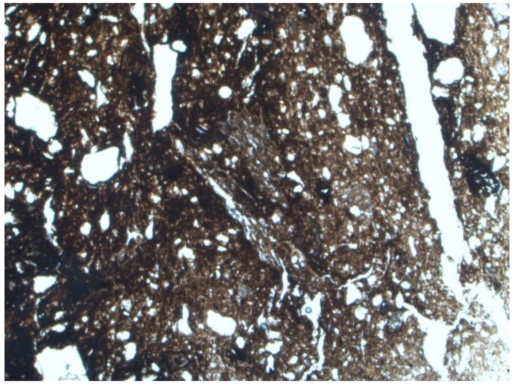
CP2.2	6.2	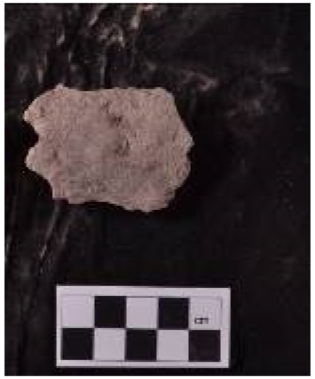	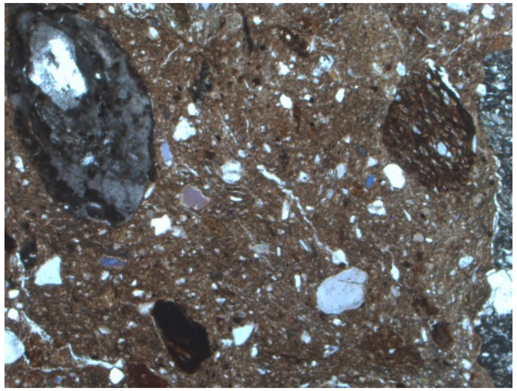	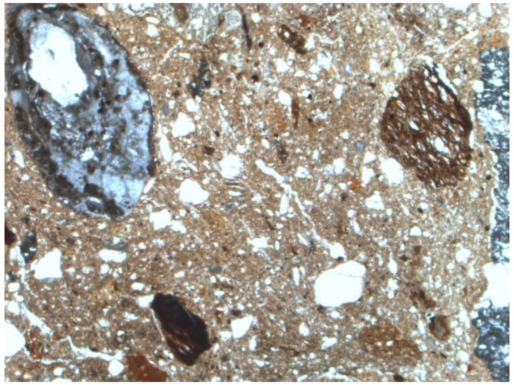

CP5	6.2	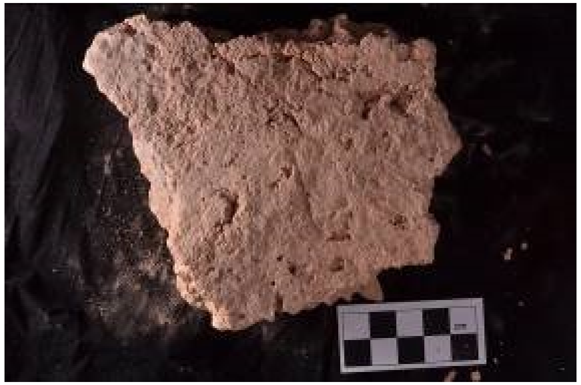	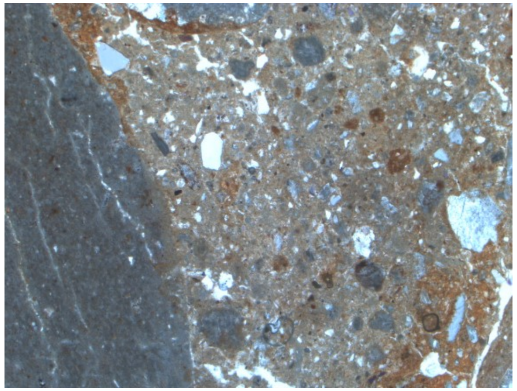	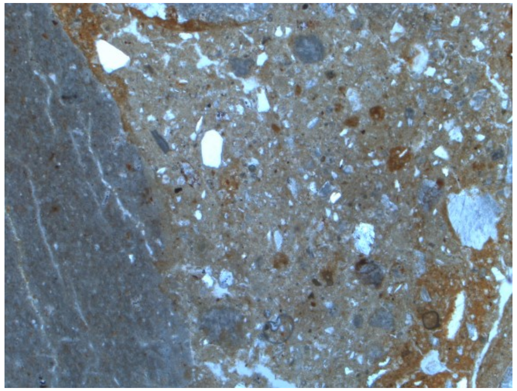

1.1 Calcareous clay C1.5

Inclusions
20%. eq & el. a-vr. <0.3 mm. Close-spaced to open-spaced. Poorly sorted. Randomly oriented. Moderately bimodal grain size distribution.

Coarse fraction
5%. 0.3-0.05 mm

Dominant:	Micrite; eq. vr. <0.3mm, mode = 0.2mm.
Common:	Quartz; el & eq. a–sa. <0.3mm, mode = 0.2mm.
Common:	Feldspar (orthoclase); el & eq. a–sa. <0.3mm, mode = 0.2mm.
Rare:	TFs; eq. r <0.3mm, mode = 0.2mm. Discordant, mid reddish-brown in XPL and PPL (x4) with clear boundaries, high optical density, iron nodules and opaques.

Fine fraction
95%. 0.05-0.01 mm

Dominant:	Micrite
Few:	Quartz
Rare:	Opaques

Matrix
79%. Calcareous. Mid brownish grey in XPL, mid greyish brown PPL (XPL x4). Optically inactive.

Voids
1% Consisting of meso vesicles. Weak alignment of voids to margins of section. Secondary calcite along the edge of the voids.

Comments
This fabric is characterised by the presence of abundant calcareous inclusions in coarse and fine fraction and within the matrix. The calcareous inclusions have a sparry texture suggesting these derive from micrite. There is only one sample with this fabric, which is a dark grey (reduced fired) slipped and painted body sherd. Due to its strongly calcareous fabric and because of its unusual surface- and firing treatments this sample is an outlier.

1.2 Clay with quartz and micrite C9.1

Inclusions
15%. eq & el. a-vr. 0.4-0.025 mm. Close-spaced to open-spaced. Poorly sorted. Randomly oriented. Moderately bimodal grain size distribution.

Coarse fraction
20%. 0.4-0.15 mm

Dominant:	Quartz; el & eq. a–sa. <0.4mm, mode = 0.2mm.
Common:	Feldspar; el & eq. a–sa. <0.4mm, mode = 0.2mm
Common:	Micrite; el & eq. r-vr. <0.3mm, mode = 0.15mm. Fine sparry texture. Pinkish to yellowish brown in XPL.
Rare:	Calcite; eq. a. 0.15mm. Twinning and high order interference colours.

Fine fraction
80%. 0.15-0.025 mm

Dominant:	Micrite
Common:	Quartz
Common:	Mica (rare muscovite)
Few:	Calcite
Rare:	Opaques

Matrix

85%. Moderately calcareous. Mid to dark brownish grey in XPL and PPL (XPL x4). Optically active.

Voids
N/A

Comments
This fabric is characterised by the presence of calcareous inclusions in coarse and fine fraction and within the matrix. The calcareous inclusions have a sparry texture suggesting these derive from micrite. It distinguishes itself from fabric 1.1 through the heterogeneity of the inclusions in coarse and fine fractions suggesting that different clays were used in its production.

2.1 Fine micrite and quartzite in a non-calcareous matrix C1.1, C1.2, C1.3, C1.4, C1.10, C1.11, C1.12, C1.14, C1.15, C6.1, C6.2, C6.3, C6.5, C7.1

Inclusions
5-12%. eq & el. a-vr. 0.3-0.05 mm. Close-spaced to open-spaced. Poorly to well-sorted. Moderately to strongly aligned to the vessel margins. Moderately bimodal grain size distribution.

Coarse fraction
50%. 0.3-0.05 mm

Common: Micrite; eq. vr. <0.3mm, mode = 0.1mm. (Few in C1.4 and C6.3)

Dominant (C1.12): Sparry calcite; el & eq. a-r. <0.3mm, mode =0.01mm

Common: Quartz; el & eq. a–sa. <0.3mm, mode = 0.1mm.

Common: Feldspar; el & eq. a–sa. <0.3mm, mode = 0.1mm

Rare: TFs; eq. r <0.3mm, mode = 0.1mm. Discordant, mid reddish-brown in XPL and PPL (x4) with clear boundaries, high optical density, iron nodules and opaques.

Fine fraction
50%. 0.1-0.01 mm
Dominant: Micrite
Common: Elongate mica
Few: Quartz
Rare: Opaques

Matrix
87-94%. Non-calcareous. Mid orangey-red to mid-greyish brown in XPL, bright orange to mid-grey in PPL (XPL x4). Optically inactive, moderate optical activity in C6.3, C7.1 and C1.12. Homogeneous. Inhomogeneity in C1.10, C1.14, C6.5 and C7.1 due to firing treatment (incomplete oxidisation) creating a grey core and orange/red vessel margins.

Voids
0-1%. Consisting of micro and meso vughs and vesicles. Strong alignment of voids to margins of section.

Comments
This fabric is characterised by the presence of fine micrite, quartz and rare iron-rich inclusions in a homogeneous, optically inactive matrix. The calcareous inclusions have a sparry texture suggesting these derive from micrite. The very fine nature of this fabric suggesting that clays were purified carefully, probably utilising levigation. The samples are fired in oxidising conditions resulting in bright orange and red surface colours. Incomplete oxidisation is visible in C1.10, C1.14, C6.5 and C7.1, resulting in a dark grey streak parallel to the margins of the sample.

The inclusions are usually strongly aligned to the vessel margins, indicating that a rotary device was used for shaping the vessels. In C1.3, C6.2 inclusions are more randomly oriented, perhaps clustering together at the coil breaks, although the sample is too small to be certain. Possibly vessels were shaped through wheel-coiling and wheel-throwing.

2.2 Fine quartz in a non-calcareous, optically active matrix C1.8, C1.9

Inclusions
5%. eq & el. a-vr. 0.25-0.01 mm. Open-spaced. Poorly sorted. Moderately aligned to the vessel margins. Moderately bimodal grain size distribution.

Coarse fraction
40%. 0.25-0.05 mm

Common: Quartz; eq. vr. 0.25-0.01mm, mode = 0.1mm.

Common: Feldspar; el & eq. a–sa. <0.3mm, mode = 0.1mm

Very rare: TFs; eq. r. 0.125-0.05mm, mode = 0.05mm. Discordant, mid reddish-brown in XPL and PPL (x4) with clear boundaries, high optical density, iron nodules and opaques.

Fine fraction
60%. 0.05-0.01 mm
Dominant: Quartz
Common: Elongate mica
Few: Opaques

Matrix
94%. Non-calcareous. Mid orangey-red in XPL, bright orange in PPL (XPL x4). Optically active. Homogeneous.

Voids
1%. Consisting of meso vughs. Strong alignment of voids to margins of section.

Comments
This fabric is characterised by fine quartz in an optically active, mica-rich matrix. The fabric differs from other fine fabrics by the absence for calcareous inclusions, indicating that different clays were used or that this is a sample imported to the site. The clay has been carefully purified and possibly levigated. The optical activity of the matrix suggests temperatures were not high enough to reach full vitrification (e.g. below 800-850°C).

2.3 Sand temper C1.7

Inclusions
24%. eq & el. a-vr. 0.6-0.025 mm. Close to open-spaced. Poorly sorted. Moderately aligned to the vessel margins. Bimodal grain size distribution.

Coarse fraction
40%. 0.6-0.1 mm

Predominant: Quartz; eq. a-r. 0.6-0.1mm, mode = 0.2mm.

Dominant: Feldspar (orthoclase); eq. a-r. 0.6-0.1mm, mode = 0.2mm

Common: Micrite; eq & el. r-vr. 0.3-0.1mm, mode = 0.2mm

Rare: TFs; eq. r. 0.125-0.1mm, mode = 0.1mm. Discordant, mid reddish-brown in XPL and PPL (x4) with clear to merging boundaries, high optical density, iron nodules and opaques.

Fine fraction
60%. 0.1-0.025 mm
Dominant: Quartz
Common: Elongate mica needles
Few: Opaques
Very rare: Subhedral muscovite

Matrix

72%. Non-calcareous. Mid orangey-brown and mid brownish-grey in XPL, mid to dark brown in PPL (XPL x4). Optically active. Streaking of clays visible in thin-section, which could be natural (as this is also visible in CG1).

Voids
4%. Consisting of macro planar voids and channels and meso vughs and vesicles. Strong alignment of voids to margins of section.

Comments
This fabric is characterised by the presence of abundant angular quartz and feldspar in a matrix with fine mica needles. The addition of temper is also suggested by the bimodal grain size distribution. There is some variation in the colour of the matrix, which could be natural, as this is also observed in the geological sample from a nearby deposit (CG1, described below).

3.1 Quartz-tempered fabric in micrite-rich matrix C1.6, C6.6, C8.1, C1.13, C7.2

Inclusions
5-18%. eq & el. a-vr. 1.25-0.05 mm. Single-spaced to open-spaced. Well sorted. Strong alignment to the vessel margins. Bimodal grain size distribution.

Coarse fraction
40%. 1.25-0.2 mm

Predominant:	Quartz; eq. a-r. 0.4-0.3mm, mode = 0.3mm.
Common:	Feldspar; el & eq. a–sa. <0.3mm, mode = 0.2mm
Common:	Micrite; eq & el. r-vr. 1.25-0.2mm, mode = 0.4mm. Well sorted, rounded inclusions of micrite
Rare-Very Rare:	TFs; eq. r. 0.3-0.2mm, mode = 0.2mm. Discordant, mid reddish-brown in XPL and PPL (x4) with clear to merging boundaries, high optical density, iron nodules and opaques. Rare in C8.1.

Fine fraction
60%. 0.2-0.05 mm

Predominant:	Micrite
Common:	Quartz
Few:	Opaques
Very rare:	Elongate mica (common in C8.1)

Matrix
80-93%. Moderate to strongly (C1.13) calcareous. Mid orangey-red, and light and mid brownish-grey in XPL, mid orangey-red to dark brown in PPL (XPL x4). Optically active. Heterogeneous. Heterogeneity due to core-margin differentiation related to firing treatment (incomplete oxidation). Homogeneous mid orangey-red in C8.1 and homogeneous mid grey in C1.13.

Voids
2%. Consisting of mego to meso vughs. Strong alignment of voids to margins of section.

Comments
This fabric is characterised by the presence of well-sorted, rounded micrite in coarse and fine fraction. Coarse fraction contains quartz and micrite of larger dimensions than the other fabrics, possibly suggesting that clays were less carefully purified. Due to the similarities in the inclusions in coarse and fine fraction it is likely that inclusions were present in the clay naturally. Nevertheless, the sorting of the inclusions suggests that clays were carefully kneaded and that larger inclusions were removed.

Within this fabric, samples C1.06 and C6.6 are very similar in terms of firing treatment, nature and sorting of the inclusions. Sample C8.1 has a red oxidised break with well-sorted inclusions and common platy mica in fine fraction. Sample C1.13 has a grey fabric colour and with more poorly sorted inclusions in coarse and fine-fraction. These were nevertheless grouped into this fabric due to the similar abundance and general

nature of inclusions in coarse and fine-fraction. Possibly, however, these fabrics point to different sites of clay procurement. Particularly C1.13 has a much lighter fabric colour due to the higher calcite content in its matrix.

3.2 Oolitic limestone and ferrogeneous inclusions C6.4

Inclusions
25%. eq & el. a-wr. 0.75-0.05 mm. Single-spaced to open-spaced. Poorly sorted. Weak alignment to the vessel margins. Bimodal grain size distribution.

Coarse fraction
40%. 1.25-0.2 mm

Predominant:	Calcareous inclusions; eq. a-r. 0.7-0.2mm, mode = 0.3mm. Composed of sparry calcite, polycrystalline calcite and oosparite. No twinning visible. Nature of the calcareous inclusions suggests that these derive from oolitic limestone bedrock.
Common:	Micrite; eq & el. r-wr. 1.25-0.2mm, mode = 0.4mm. Well sorted, rounded inclusions of micrite.
Common:	TFs; eq. sr-wr. 0.75-0.2mm, mode = 0.3mm. Discordant, mid reddish-brown to dark brown in XPL and PPL (x4) with sharp to diffuse boundaries, high optical density, iron nodules and opaques.
Few:	Quartzite; eq. sa-sr. 0.7-0.2mm, mode = 0.3mm. Composed of well-sorted quartz and weathered mica.

Fine fraction
60%. 0.2-0.05 mm

Predominant:	Sparite
Common:	Quartz
Few:	Opaques
Very rare:	Elongate mica

Matrix
73%. Moderately-calcareous. Mid orangey-brown in XPL and PPL (XPL x4). Optically active. Homogeneous.

Voids
2%. Consisting of mega channels which are strongly aligned to the margins of the section.

Comments
This fabric is characterised by the presence of poorly sorted calcareous inclusions that derive from oolitic limestone. The bimodal grainsize distribution and abundance of inclusions suggests fine calcareous sand might have been added as temper to purified non-calcareous clay.

Hand-made

4.1 Shale tempered fabric C3.1, C4.1, C4.2, C4.3, C10.2, C11.1, C11.2, C12.1, C13.4, C13.5

Inclusions
20-30%. eq & el. a-wr. 11.0-0.05 mm. Close to single-spaced. Poorly sorted. Weak to moderate alignment to the vessel margins. Some alignment along margins of coils. Bimodal grain size distribution.

Coarse fraction
60%. 11-0.2 mm

Predominant:	Shale; el. sa-sr. 11.0-0.2mm, mode = 1.0mm. Composed of coarse to medium inclusions of shale. Lamellar microstructure with coarser (±0.1mm-wide) grains of rounded sericite mica inclusions. Some of the more mica-rich inclusions are light in colour (light-greyish brown in XPL). Weathered fragments have a dark-brown colour. Sericite formed by the alteration of feldspar.

Common:	Micrite; eq & el. r-wr. 8.0-0.2mm, mode = 0.4mm. Well sorted, rounded inclusions of sparry calcite. Some are or contain microfossils (oolites).
Few:	TFs; eq. sr-wr. 5.0-0.2mm, mode = 0.3mm. Discordant, mid reddish-brown to dark brown in XPL and PPL (x4) with sharp to diffuse boundaries, high to neutral optical density, iron nodules, opaques, reddish-brown to dark-brown clay pellets with fine quartz and coarser clay pellets concordant clay pellets with sharp to merging boundaries. These are very common in C4.3 particularly.
Common:	Quartz; eq. sa-sr. 0.425-0.2mm, mode = 0.25mm. Composed of well-sorted, occasionally polycrystalline quartz.
Common:	Feldspar; el & eq. a–sa. <0.3mm, mode = 0.2mm
Rare:	Calcite; eq & el. sa-sr. 0.7-0.2mm, mode = 0.3 mm. Lamellar twinning.

Fine fraction
40%. 0.2-0.05 mm

Predominant:	Quartz
Common:	Opaques
Few:	Micrite
Few:	Shale
Few:	Iron nodules

Matrix
65-78%. Non-calcareous. Mid orangey-brown, reddish-brown and greenish-grey in XPL, greenish-grey to light-orangey brown and dark grey (C12.1, C13.8) in PPL (XPL x4). Optically active. Heterogeneous. Heterogeneity due to streaking, clay pellets, variation in density of inclusions in fine fraction and colour variation. Less obvious streaking in C3.1 and C4.1. Light fabric colours indicate the samples were fired in oxidising conditions.

Voids
2-5%. Consisting of mega channels which align to the margins of the section, encircle clay pellets and some inclusions. Aligned channels are the result of shrinkage of the matrix during firing.

Comments
This fabric is characterised by the presence of very coarse fine-grained clastic inclusions identified as shale. The shale is often micaceous with individual fine grains visible in the microstructure. This is probably sericite/muscovite created by the alteration of feldspar. Shale is represented in the geology of the town of Cella, some 3km south of El Cerrito. It is likely that local shale was crushed and added as a tempering agent. The shale inclusions are often strongly aligned to the margins of the section, particularly the larger fragments. Smaller fragments are more randomly oriented. Some inclusions seem to be aligned with coil or slab joins.

The matrix of most samples is heterogeneous due to elongate streaking of reddish-brown clay in a yellow and greenish-grey matrix. Streaking has also been observed in clay sample 1, deriving from a ditch nearby the site (fabric description below). Instead of indicating clay mixing it is therefore likely that the streaking observed in the ceramic samples of this fabric is natural to the clays used. It is likely that similar clays were used as those in fabric 3 and 4.2 given the nature of the inclusions. The larger micrite inclusions often contain oolites. Oolitic limestone is common north of the site as well as calcite and dolomite deposits. The fabric thus suggests that these ceramics were produced on site. Light fabric colours suggest the samples were fired in oxidising conditions. SEM imagery demonstrates that the fabric underwent sintering, suggesting the firing conditions were comparable to the wheel-made fabrics.

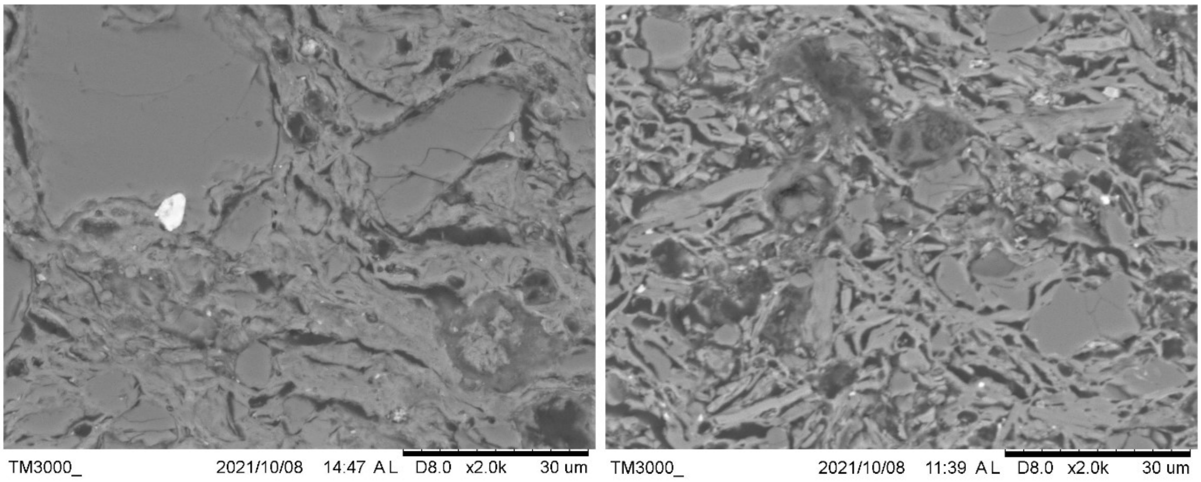

Fig S2.1 SEM photograph of sintering matrix of C11.2 (fabric 4.1, left) and C7.1 (fabric 2.1, right).

4.2 Shale and grog tempered fabric C10.1, C12.2, C12.3, C13.6, C13.7, C13.8, C14.1, C14.2, C14.3

Inclusions
20-30%. eq & el. a-wr. 11.0-0.05 mm. Close to single-spaced. Poorly sorted. Weak to moderate alignment to the vessel margins. Some alignment along margins of coils. Bimodal grain size distribution.

Coarse fraction
60%. 11.0-0.2 mm

Predominant: Shale; el. sa-sr. 11.0-0.2mm, mode = 1.0mm. Composed of coarse to medium inclusions of shale. Lamellar microstructure with coarser (±0.1mm-wide) grains of rounded sericite mica inclusions. Some of the more mica-rich inclusions are light in colour (light-greyish brown in XPL). Weathered fragments have a dark-brown colour. Sericite formed by the alteration of feldspar.

Common: Micrite; eq & el. r-wr. 8.0-0.2mm, mode = 0.4mm. Well sorted, rounded inclusions of micrite. Some are or contain microfossils (oolites).

Few: TFs; eq. sr-wr. 5.0-0.2mm, mode = 0.3mm. Discordant, mid reddish-brown to dark brown in XPL and PPL (x4) with sharp to diffuse boundaries, high to neutral optical density, iron nodules, opaques, reddish-brown to dark-brown clay pellets with fine quartz and coarser clay pellets concordant clay pellets with sharp to merging boundaries. These are very common in C04.3 particularly.

Few: Grog; el & eq. a-sr. 5.0-0.3mm, mode = 0.7mm. Discordant, mid to dark grey in XPL and PPL (x4) with sharp to clear boundaries and high to neutral optical density. Contains quartz, opaques, rare micrite and rare mica and rare sandstone or shale inclusions. The shape and angularity of the inclusions suggests they are grog instead. Possibly this is of reduced fired hand-made fine-ware pottery.

Common: Quartz; eq. sa-sr. 0.425-0.2mm, mode = 0.25mm. Composed of well-sorted, occasionally polycrystalline quartz.

Common: Feldspar; el & eq. a–sa. <0.4mm, mode = 0.2mm

Rare: Calcite; eq & el. sa-sr. 0.7-0.2mm, mode = 0.3 mm. Lamellar twinning.

Very rare: Quartzite (sample C12.2); eq. sa. 7.0mm. Very coarse fragment of polycrystalline quartz arenite which is moderately weathered.

Fine fraction
40%. 0.2-0.05 mm
Predominant: Quartz

Common:	Opaques
Few:	Marl
Few:	Shale
Few:	Iron nodules

Matrix
65-78%. Non-calcareous. Mid orangey-brown, reddish-brown and greenish-grey and dark-brownish grey in XPL, greenish-grey to light-orangey brown and dark grey (C12.1, C13.8) in PPL (XPL x4). Optically active. Heterogeneous. Heterogeneity due to streaking, clay pellets, variation in density of inclusions in fine fraction and colour variation. Dark brownish-grey colour in C10.1 due to the presence of charred organic material. Dark fabric colours suggest that the samples were fired in mixed and reducing atmospheres.

Voids
2-5%. Consisting of mega channels which align to the margins of the section, encircle clay pellets and some inclusions. Very rare organic inclusions in C10.1, where voids have appeared around charred plant material. Aligned channels result from the shrinkage of the clay during firing.

Comments
This fabric is characterised by the presence of very coarse fine-grained clastic inclusions identified as shale and grog. This fabric is similar in every way to the samples from fabric 5.1 except for the presence of grog. The fabric of the grog has relatively fine inclusions as compared to the fabric in which it is embedded. The inclusions in the grog are, however, not as fine and homogeneous as those of the wheel-made pottery category. Further, because the grog fragments are grey, they may derive from reduced fired hade-made pottery of a fine-ware category not included in the current sample.

5. Grog and calcite temper C13.1, C13.2, C13.3

Inclusions
35%. eq & el. va-wr. 1.75-0.05 mm. Close to single-spaced. Poorly sorted. Weak to moderate alignment to the vessel margins. Bimodal grain size distribution.

Coarse fraction
80%. 1.75-0.25 mm

Predominant:	Calcareous inclusions; el & eq. va-sa. 1.75-0.25mm, mode = 0.3mm. Often Rhomboidal with parallel twinning. Well-sorted. Some more micritic and polycrystalline calcite fragments.
Common:	Grog; el & eq. a-sr. 1.75-0.5mm, mode = 0.7mm. Discordant and concordant, mid to dark grey and light brown in XPL and PPL (x4) with sharp to clear boundaries and high optical density. Well sorted grog fragments containing calcite and dolomite inclusions, micrite and quartz. Often prolate and equant but sometimes more rounded.
Common:	TFs: el & eq. sa-wr. Concordant. Light brown in XPL and PPL (4x) with clear to merging boundaries and neutral to low optical density. Composed of sub-angular to well-rounded clay pellets, some containing dolomite and quartz, others containing fine rounded micrite inclusions. Probably deriving from the mixing of calcite-rich clay and/or ARFs with the non-calcareous matrix. Prolate, concoidal, equant and rounded fragments suggesting a heterogeneous mixture of TFs which could include both grog, ARFs and clay pellets. Possibly the mixture derives from unfired and crushed vessel fragments.
Very few:	Fine-grained clastic rock fragments (shale); el. sa-sr. 1.75mm and 0.75mm in CT13.3 and C13.2 respectively. Composed of coarse to medium inclusions of shale. Lamellar microstructure with a dark-brown colour.
Few:	TFs; eq. sr-wr. 0.5-0.2mm, mode = 0.3mm. Discordant, black in XPL and PPL (x4) with sharp boundaries, high optical density. Opaques.
Very few:	Quartz; eq. sa-sr. 0.5-0.2mm, mode = 0.3mm.

Very few:	Feldspar; el & eq. a–sa. <0.5mm, mode = 0.3mm

Fine fraction
20%. 0.2-0.05 mm

Predominant:	Quartz
Few:	Calcite
Few:	Opaques

Matrix
58%. Non-calcareous. Dark to light brown and mid-orangey brown in XPL, Light to dark-brown in PPL (XPL x4). Optically active. Heterogeneous. Heterogeneity due to core-margin variation in C13.1 and C13.2 and due to heterogeneous grog inclusions.

Voids
7%. Consisting of abundant planar voids and channels which align to the margins of the section, encircle clay pellets and some inclusions.

Comments
This fabric is characterised by the presence of abundant TFs including grog and clay pellets, and calcareous inclusions. The calcareous inclusions could be dolomite, and were probably added to the clay as temper because they appear well-sorted and angular and because few calcite fragments are present in fine-fraction. Grog fragments contain finer calcareous fragments than the surrounding matrix suggesting that crushed ceramics produced from calcite-rich clay, as well as fragments of crushed dolomite limestone, were added to a non-calcareous clay. Some clay pellets have a calcareous matrix suggesting that, alongside crushed grog, clay mixing might also have occurred.

Compared to fabric 5 the grog in this fabric is different in nature as it often contains dolomite, fewer quartz and no shale. Because dolomite is represented in the local geology around the site it is possible that this is a locally produced fabric. However, the reuse of dolomite-rich ceramics as grog distinguishes this fabric from the shale-rich grog in fabric 5.1. Another difference with fabric 5.1 is that fabric colours are often darker, pointing to firing in mixed atmospheres. It is therefore likely that these ceramics were fired in a bonfire instead of a kiln. The production process underpinning this fabric might thus be altogether different from the ceramics produced in the workshop, suggesting that these ceramics could have been brought in from a production location elsewhere, perhaps serving as cooking pottery.

6.1 Calcareous building material CBM10

Inclusions
10%. eq & el. a-wr. 5.0-0.025 mm.

Coarse fraction
60%. 5.0-0.25 mm

Predominant:	Weathered limestone; eq & el. r-wr. 1.5-0.05mm, mode = 0.5mm. Rounded sparry and polycrystalline calcite.
Few:	TFs; eq. r-wr. 5.0-0.05mm, mode = 1.3mm. Concordant, mid to dark brown in XPL and PPL (x4) with sharp to diffuse boundaries, neutral optical density. Clay pellets.
Few:	Quartz; eq. sa-sr. 0.4-0.05mm, mode = 0.2mm.
Few:	Feldspar; el & eq. a–sa. <0.4mm, mode = 0.2mm
Very rare:	Shells; el. sa-sr. 0.6-0.1mm, mode = 0.2mm.

Fine fraction
40%. 0.05-0.025 mm

Predominant:	Quartz
Common:	Feldspar
Few:	Sparite
Few:	TFs

Matrix

65-78%. Calcareous. Mid greyish-brown in XPL and PPL (XPL x4). Optically active. Heterogeneous due to clay pellets.

Comments
This fabric is represented by a piece of unfired calcareous clay utilised in the construction of the kiln. The use of the kiln will have heated the clay, affecting the calcite in the fabric. Shells might have been added as a tempering agent though it is likely they are present naturally in the calcareous matrix.

6.2 Calcareous clay with rounded oolitic limestone CBM1, CBM2.1, CBM2.2, CBM3, CBM5

Inclusions
30%. eq & el. wr. 13-1.25 mm.

Coarse fraction
60%. 130-20 mm

Predominant: Fossiliferous biomicrite; eq & el. r-wr. 13-2mm, mode = 4mm. Rounded micrite pebbles with microfossils and oolites.

Fine fraction
40%. 0.05-0.025 mm
Predominant: Micrite
Common: Quartz
Common: Calcite
Common: Sponge pellets (in CBM1)
Few: Ferruginous inclusions

Matrix
70%. Calcareous. Mid orangey-brown in XPL and PPL (XPL x4). Optically active. Heterogeneous due to clay pellets.

Comments
These are pieces of fired pottery that were used in the construction of the kiln. They are made from calcareous clay tempered with limestone pebbles. The clay and temper probably derive from the same location, near the site. The limestone are probably biomicrite as they contain abundant bioclasts (shells and oolites). The fabric differs from the other hand-made pottery due to the usage of calcareous clay and a gritty temper, probably deriving from loose alluvial deposits. The differences between the fabrics of building materials (both adobe and ceramic building material) confirms that the clay procurement and preparation strategies for the construction of the kiln and for the production of hand-made, or wheel-made, pottery do not overlap.

GEOLOGICAL SAMPLES

CG1

Non-calcareous clay with fine quartz and feldspar inclusions

Inclusions
8%. eq & el. a-wr. 0.2-0.02 mm. Single to close-spaced. Moderately sorted. Unimodal grainsize distribution.

Coarse fraction
30%. 0.2-0.02 mm

Predominant: Quartz; el & eq. a-r. 0.1-0.02mm, mode = 0.05mm.

Dominant: Feldspar; el & eq. a-r. 0.1-0.02mm, mode = 0.05mm.

Common: TFs (iron nodules); el & eq. r-wr. 0.2-0.02mm, mode = 0.2mm.

Fine fraction
70%. 0.2-0.05 mm

Dominant:	Quartz
Common:	Feldspar
Common:	Opaques and iron nodules
Few:	Elongate mica needles

Matrix
92%. Non-calcareous. Mid orange-brown, reddish-brown and grey in XPL and PPL (x4). Heterogeneous, greyish streaking through the reddish-brown matrix.

Comments
Non-calcareous clay with fine quartz and feldspar inclusions, ferruginous inclusions and fine mica needles. This is a likely source for the clay used in the production of wheel-made pottery due to its very fine matrix, which contains similar inclusions (quartz, feldspar and mica). The clay is naturally fine and probably needed little processing. Due to the natural heterogeneity of the clay it is likely that it was carefully mixed and tempered (in the case of fabric 2.3). This is also a possible source for the clays used in the production of hand-made pottery, to which shale and grog were added.

CG4

Calcareous clay with fine micrite inclusions

Inclusions
10%. eq & el. a-wr. 1.75-0.02 mm. Single to close-spaced. Moderately sorted. Unimodal grainsize distribution.

Coarse fraction
30%. 0.2-0.02 mm

Common:	Calcite; el & eq. r-wr. 1.75-0.02mm, mode = 0.75mm. Rounded weathered calcite inclusions.
Few:	TFs (iron nodules); el & eq. r-wr. 0.75-0.02mm, mode = 0.5mm.

Fine fraction
70%. 0.2-0.05 mm

Dominant:	Calcite
Common:	Opaques and iron nodules
Common:	Elongate mica needles

Matrix
90%. Calcareous. Mid orange-brown and greyish-brown in XPL and PPL (x4). Heterogeneous due grey-firing weathered calcite and micrite.

Comments
Calcareous clay with calcite inclusions and iron nodules.

Fabric descriptions of Late Iron Age ceramics from Monte Bernorio (Aguilar de Campoo, Palencia)

Table S2.3. Fabrics and associated samples

**Type**	**Fabric**	**Description**	**Samples**
Wheel-made	1.1	Fine sand fabric (*n* = 11)	MB1.2, MB1.3, MB1.4, MB1.5, MB1.6, MB1.7, MB1.8, MB1.11, MB1.13, MB1.16, MB1.19
Wheel-made	1.2	Moderately ferrous clay with fine sand (*n* = 7)	MB1.1, MB1.9, MB1.15, MB1.17, MB1.18, MB1.20, MB1.21
Wheel-made	1.3	Sand temper (*n* = 2)	MB1.10, MB1.12
Hand-made	2.1	Crushed gypsum (*n* = 1)	MB2.20
Hand-made (incised dec)	2.2	Crushed metamorphic inclusions (*n* = 1)	MB2.3
Hand-made	2.3	Calcareous clay with sparry limestone inclusions (*n* = 1)	MB2.10
Hand-made	3.1	Clay pellets/grog (*n* = 3)	MB2.16, MB2.25, MB2.27
Hand-made	3.2	Clay pellets/grog and calcareous inclusions (*n* = 2)	MB2.17, MB2.23
Hand-made	4	Calcite temper (*n* = 19)	MB2.1, MB2.2, MB2.4, MB2.5, MB2.6, MB2.7, MB2.8, MB2.9, MB2.11, MB2.12, MB2.13, MB2.14, MB2.15, MB2.18, MB2.19, MB2.21, MB2.22, MB2.24, MB2.26
Building material	5	Calcareous clay with microfossils (*n* = 3)	MBMW1.1, MBMW1.2, MBMW2.3

Table S2.4. Microphotographs of the Monte Bernorio fabrics.

**Sample nr.**	**Petro Fabric**	**Image** (not to scale)	**Micro-photograph** XPL, field of view: 3.0mm	**Micro-photograph** PPL, field of view: 3.0mm
MB1.1	1.2	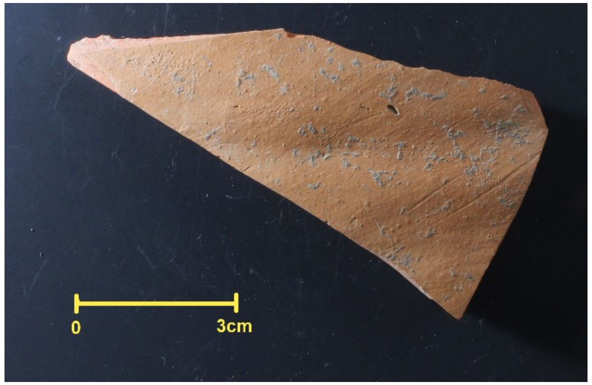	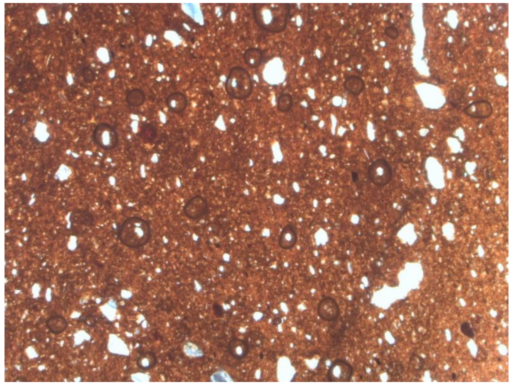	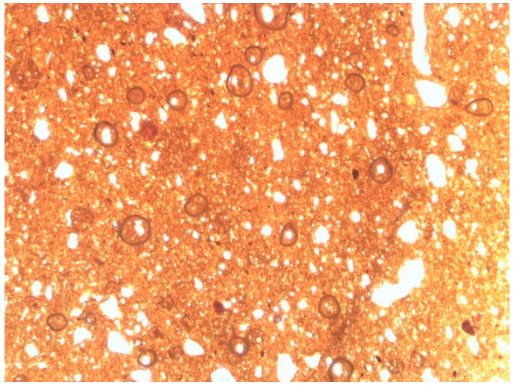
MB1.2	1.1	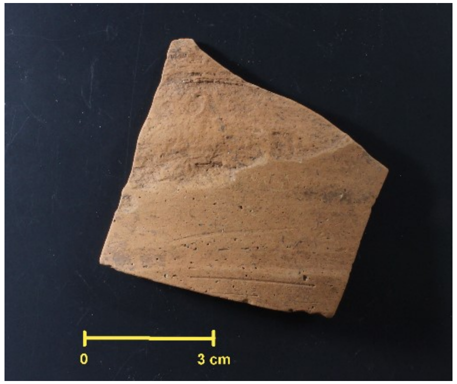	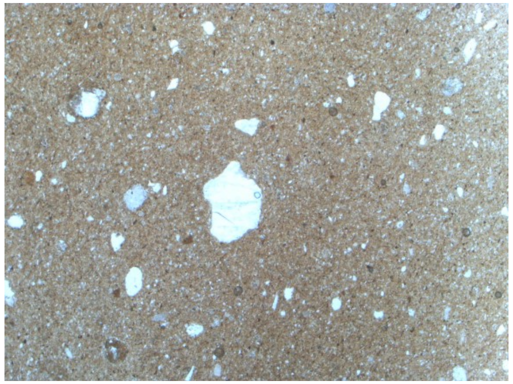	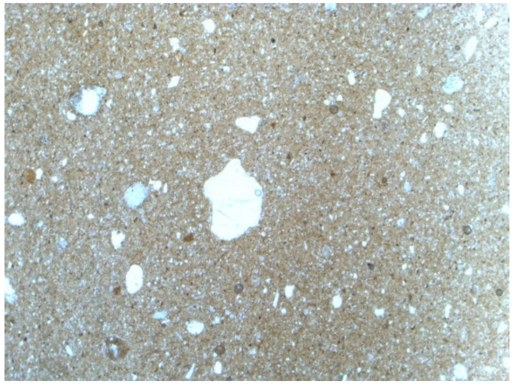

MB1.3	1.1	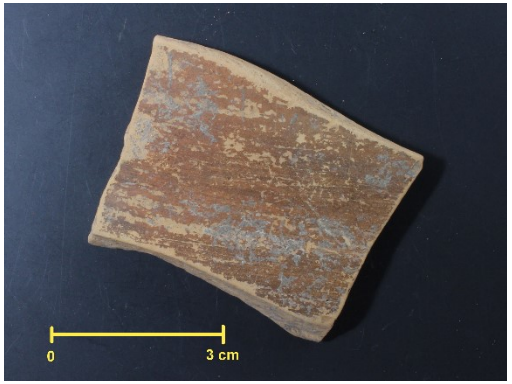	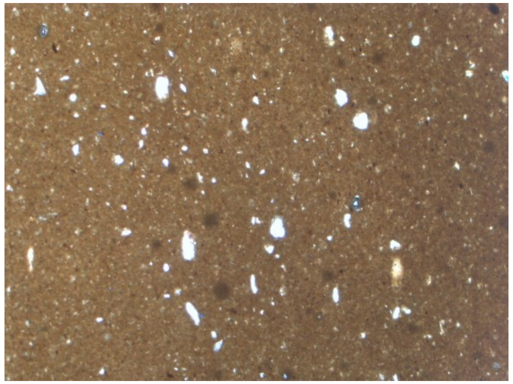	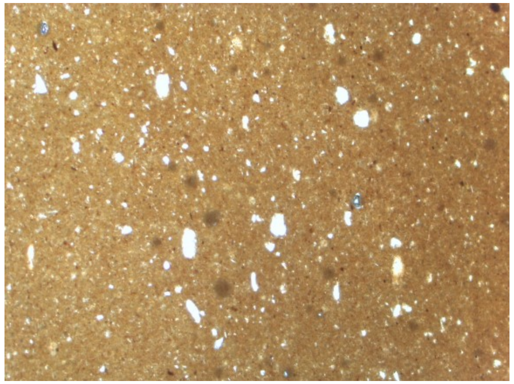
MB1.4	1.1	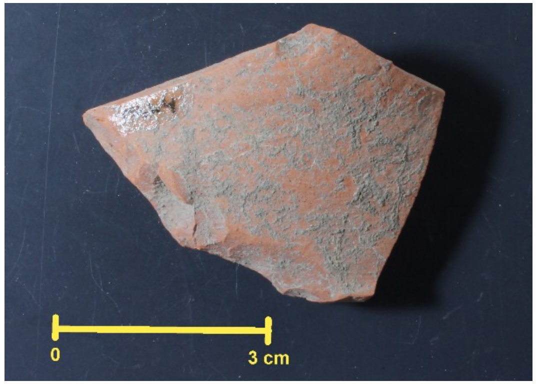	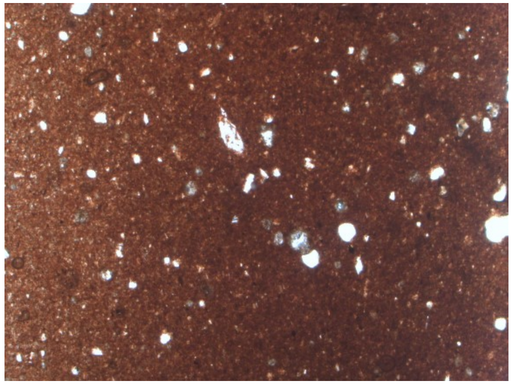	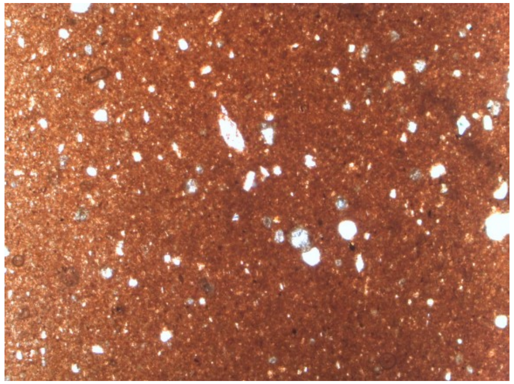
MB1.5	1.1	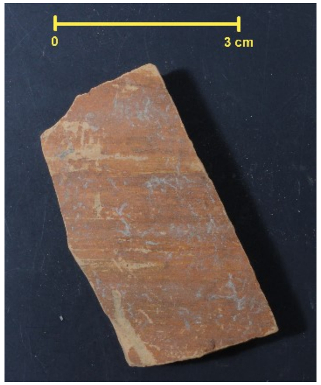	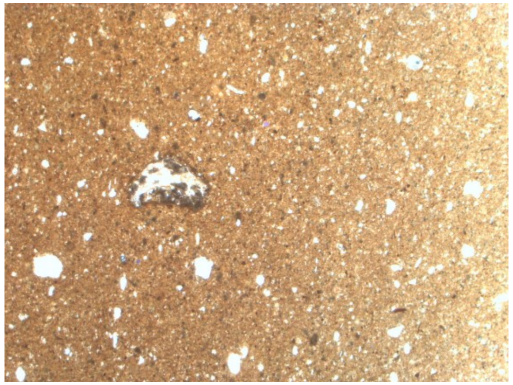	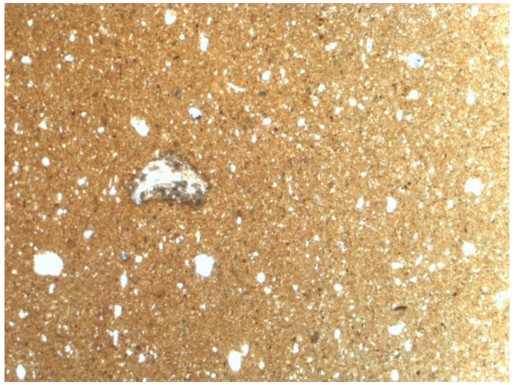

MB1.6	1.1	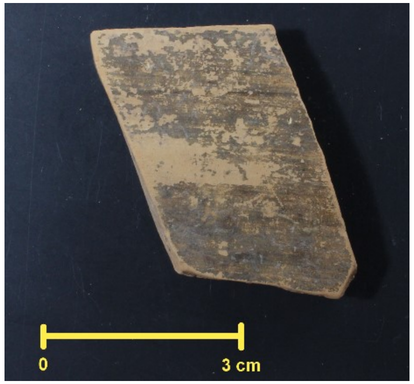	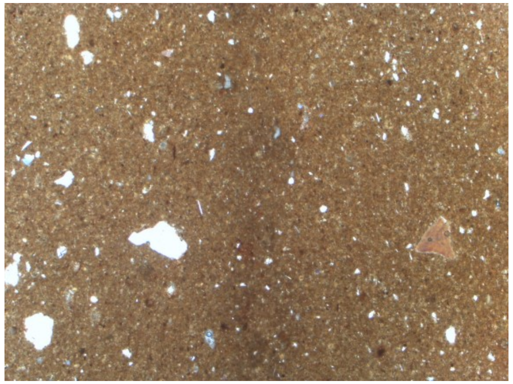	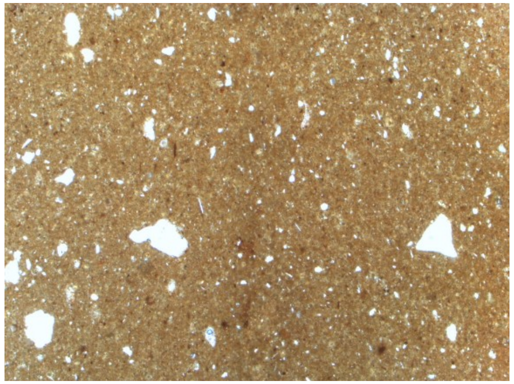
MB1.7	1.1	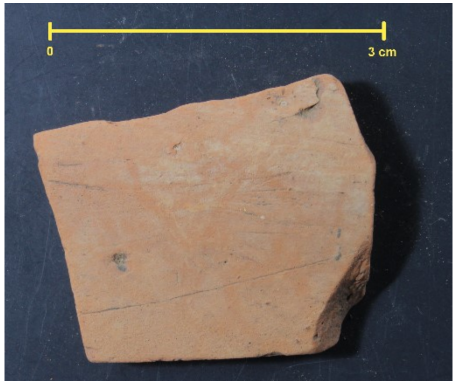	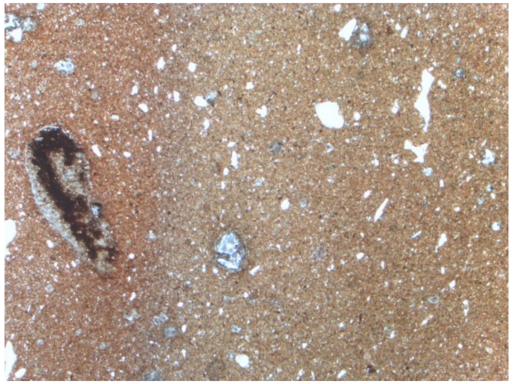	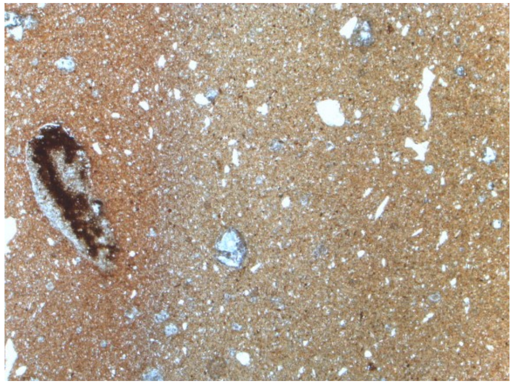
MB1.8	1.1	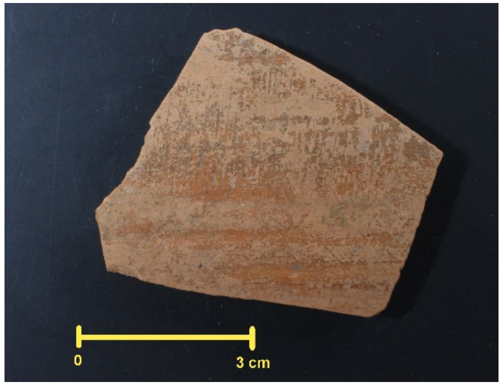	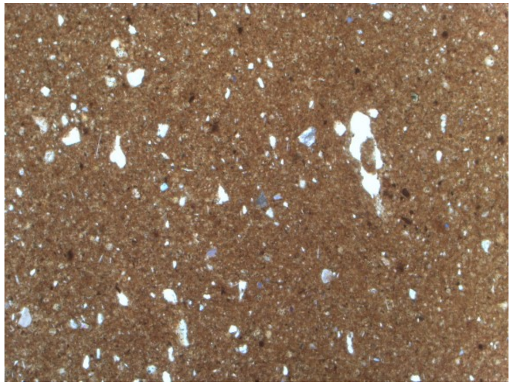	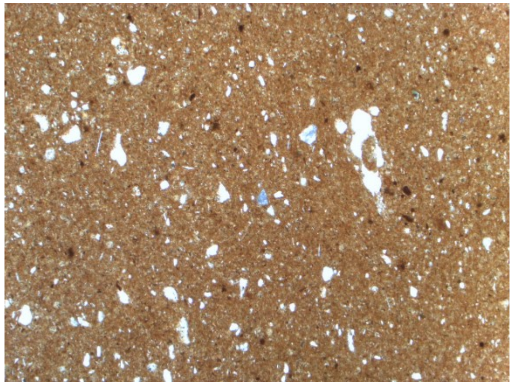

MB1.9	1.2	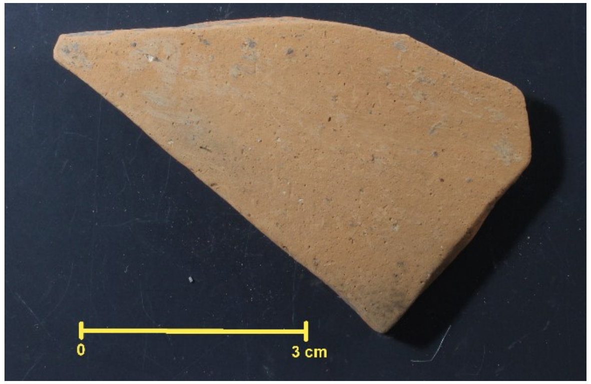	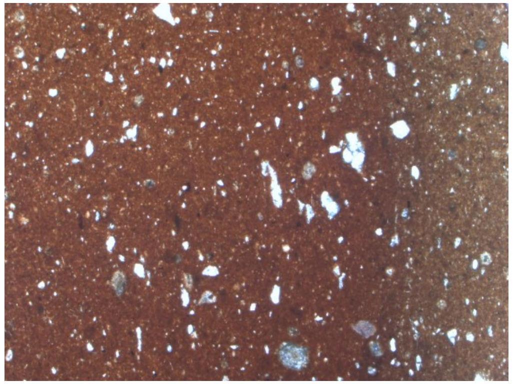	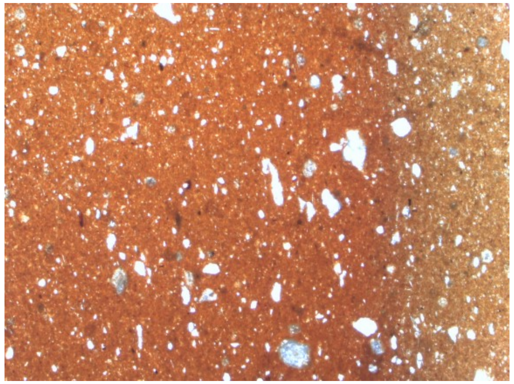
MB1.10	1.3	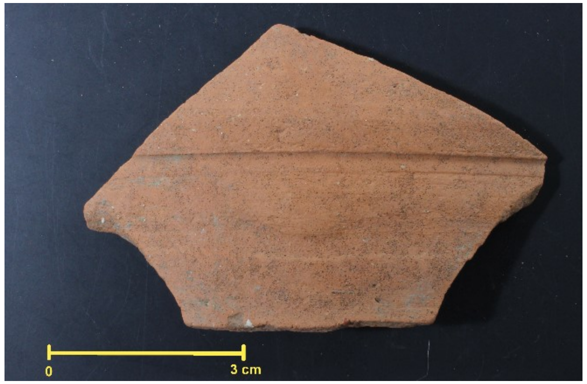	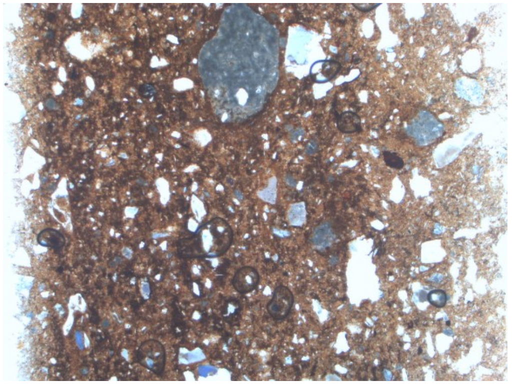	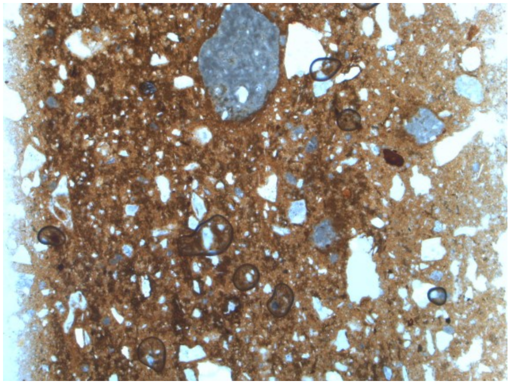
MB1.11	1.1	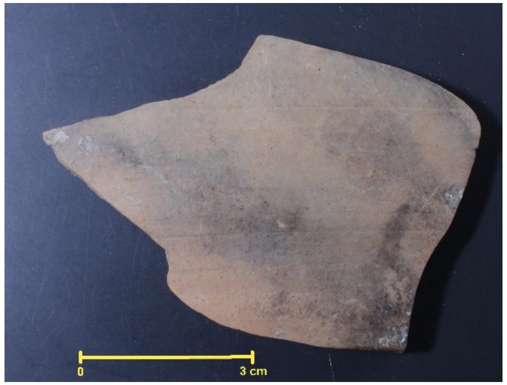	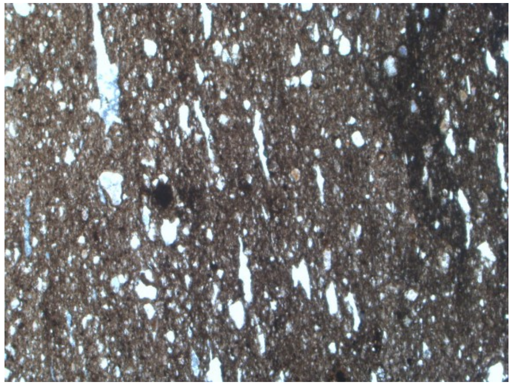	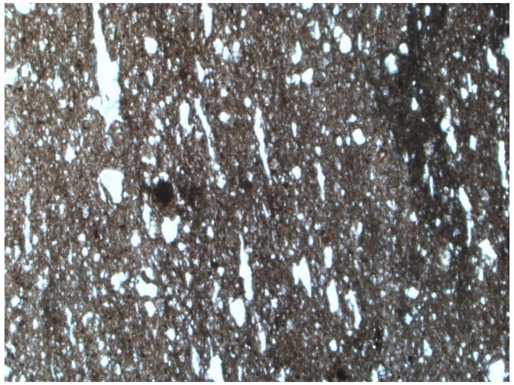

MB1.12	1.3	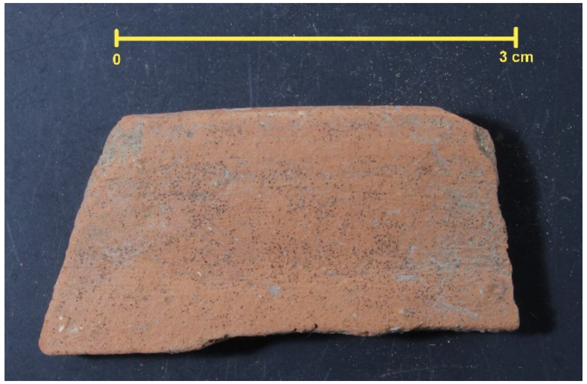	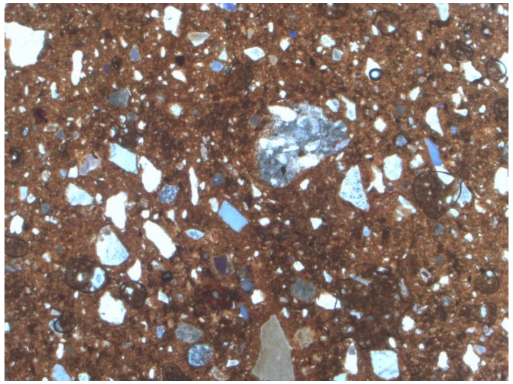	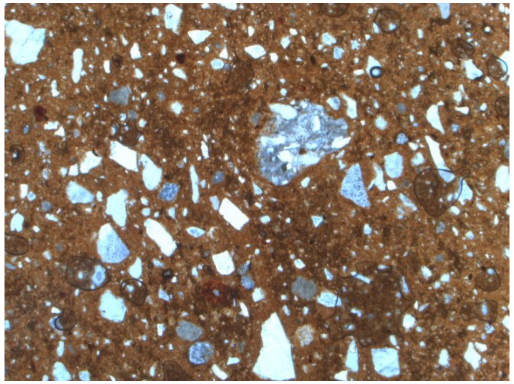
MB1.13	1.1	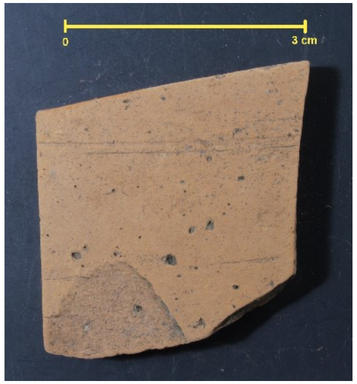	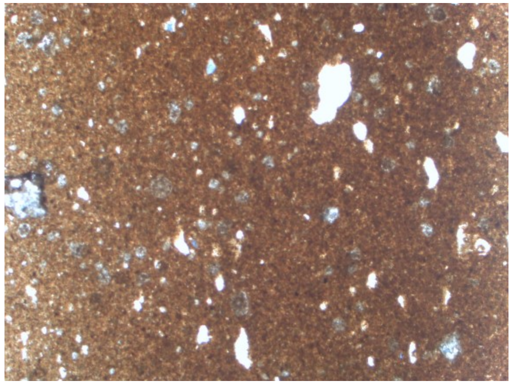	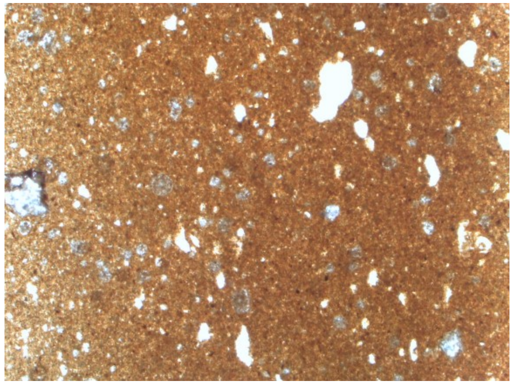
MB1.14	1.1	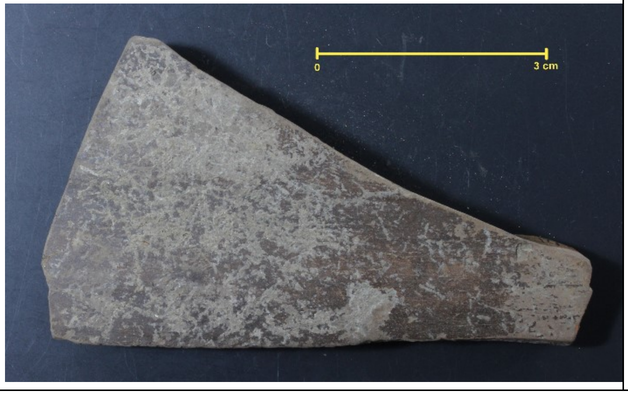	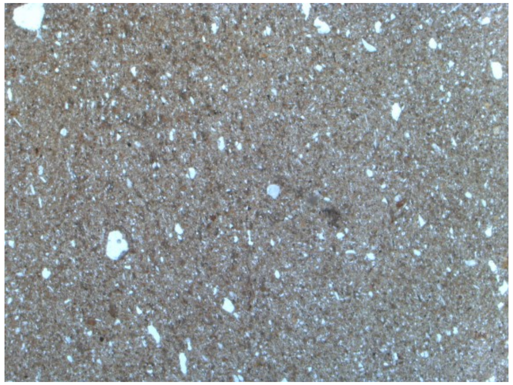	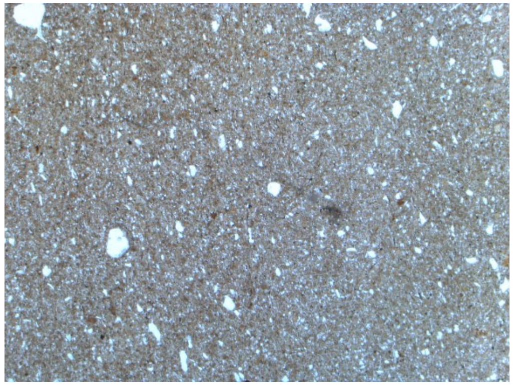

MB1.15	1.2	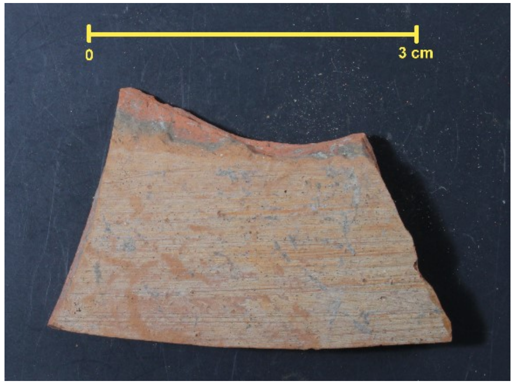	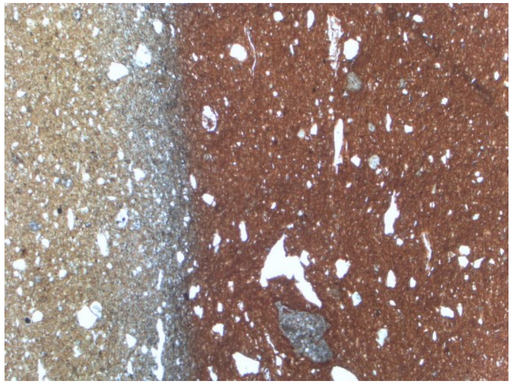	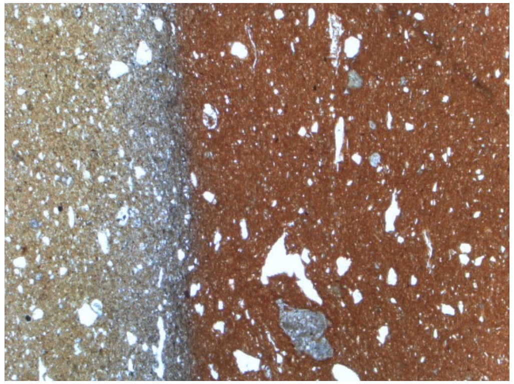
MB1.16	1.1	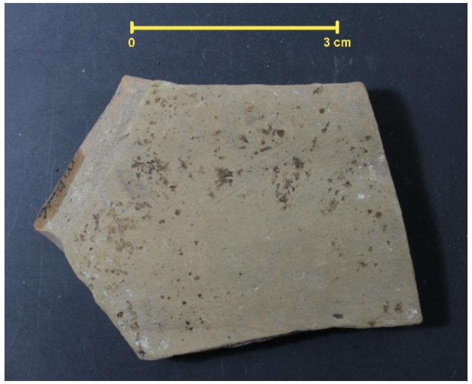	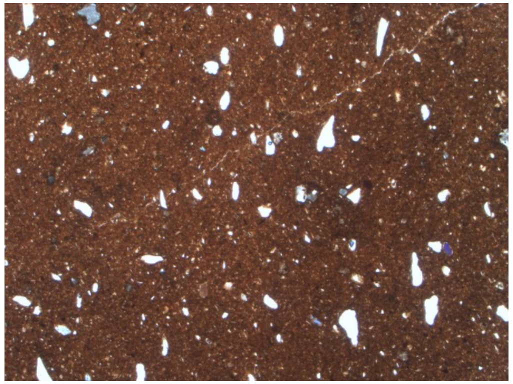	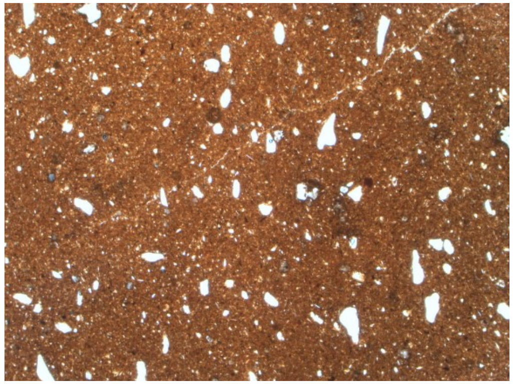
MB1.17	1.2	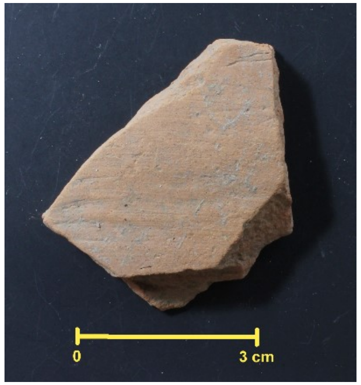	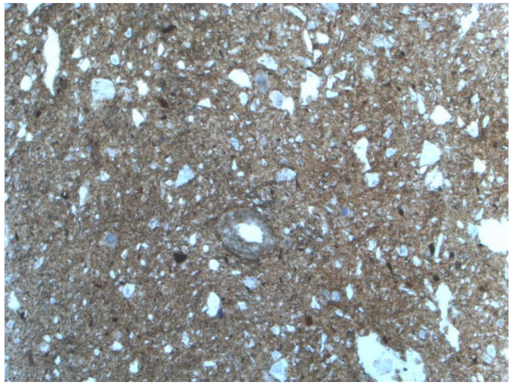	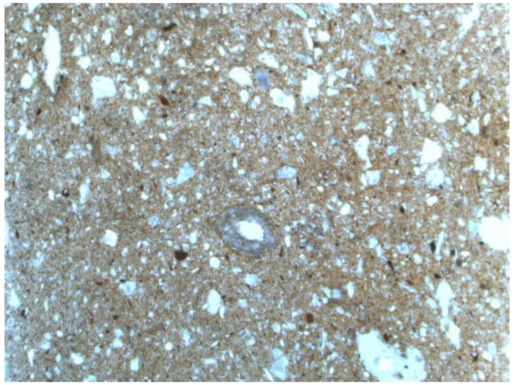

MB1.18	1.2	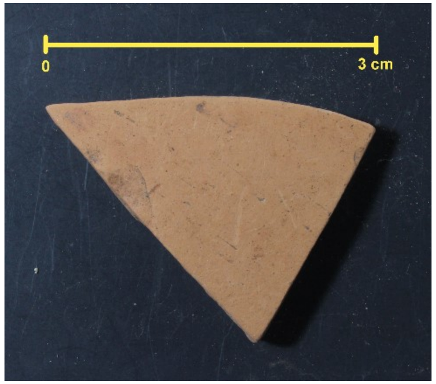	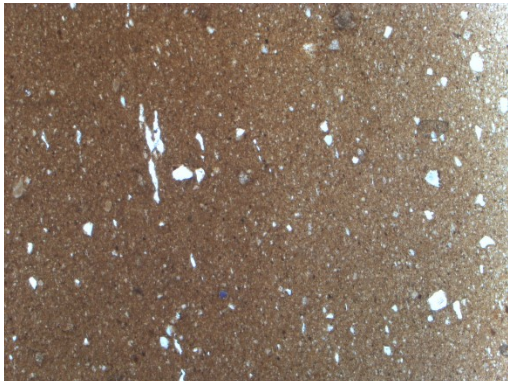	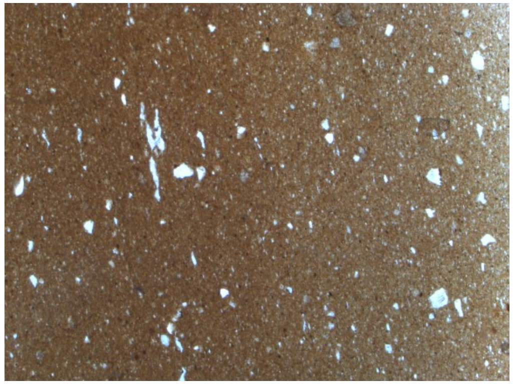
MB1.19	1.1	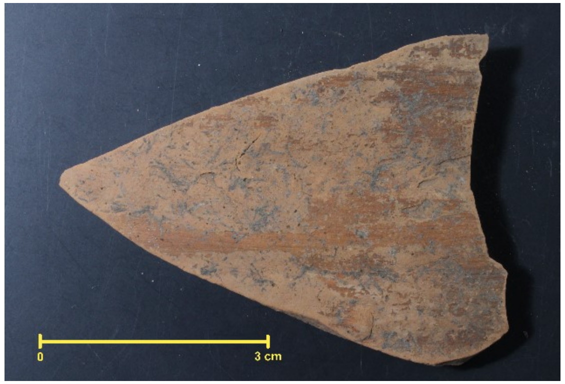	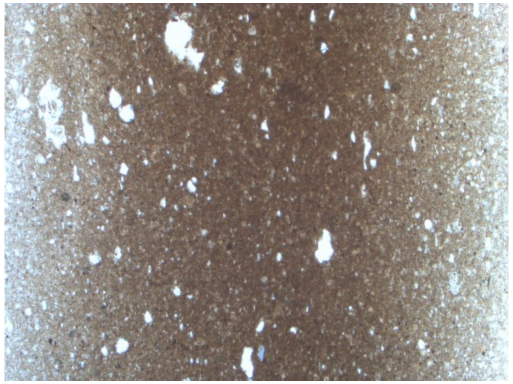	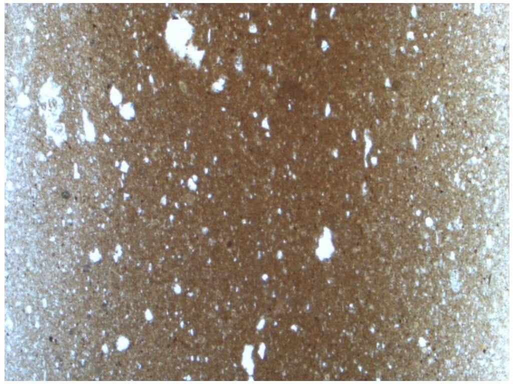
MB1.20	1.2	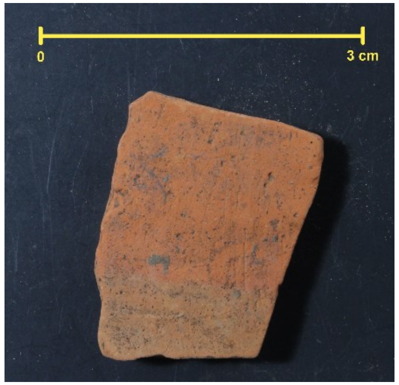	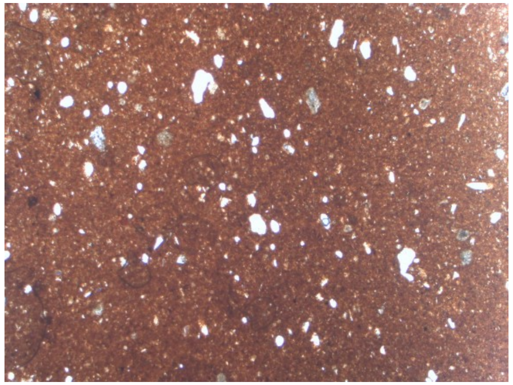	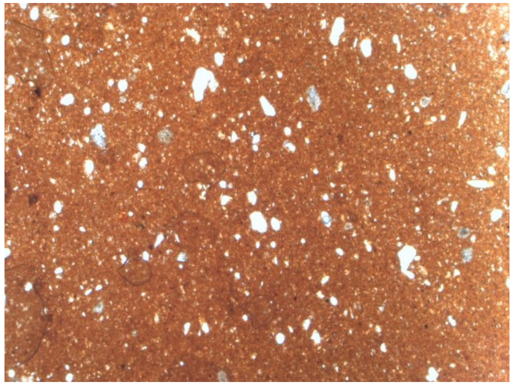

MB1.21	1.2	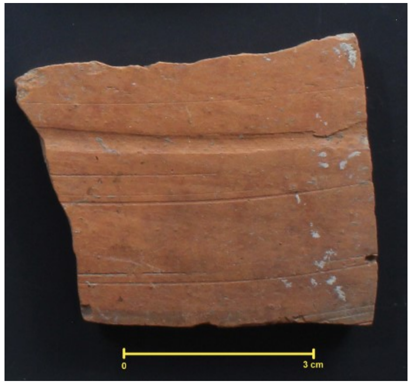	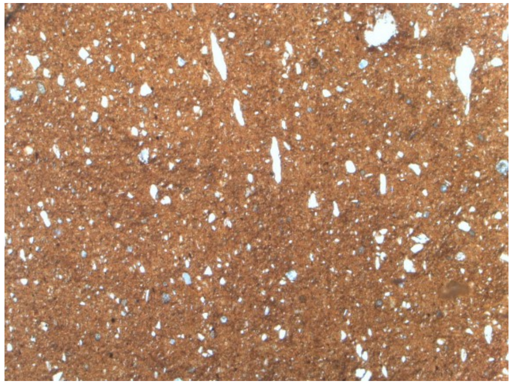	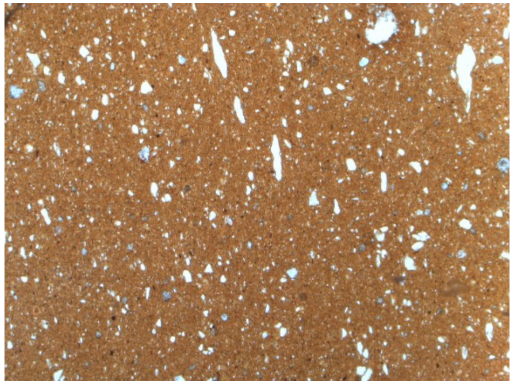
MB2.1	4	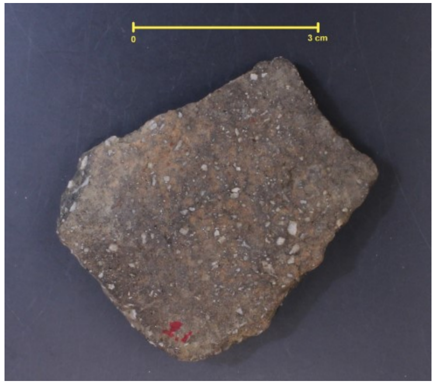	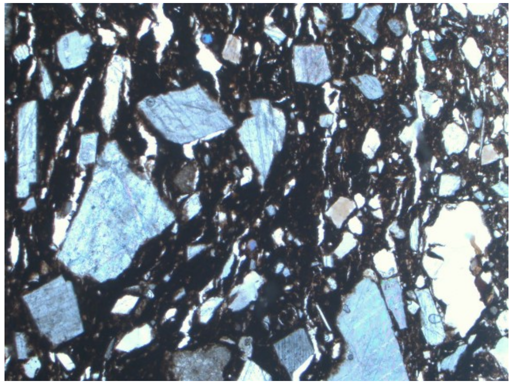	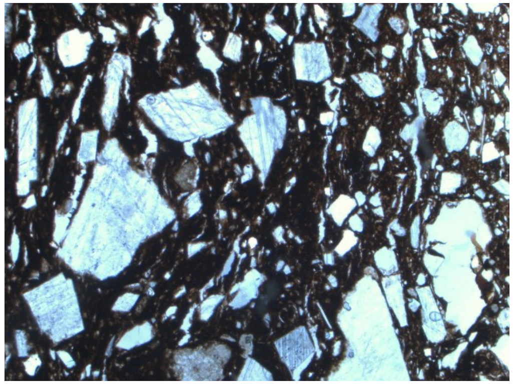
MB2.2	4	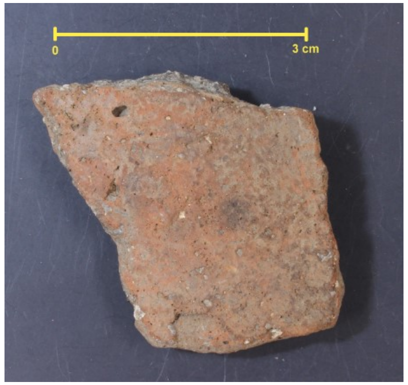	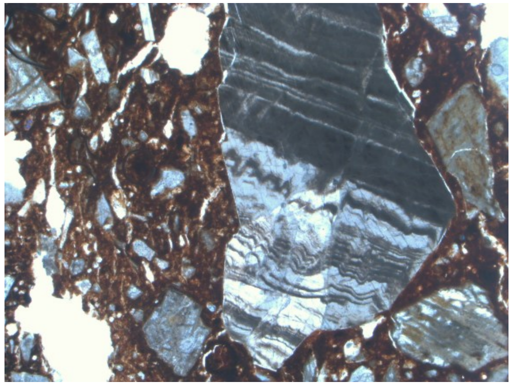	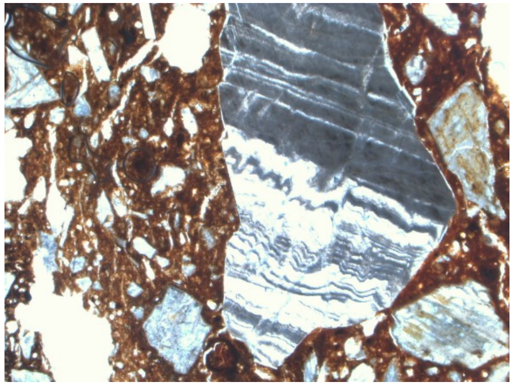

MB2.3	2.2	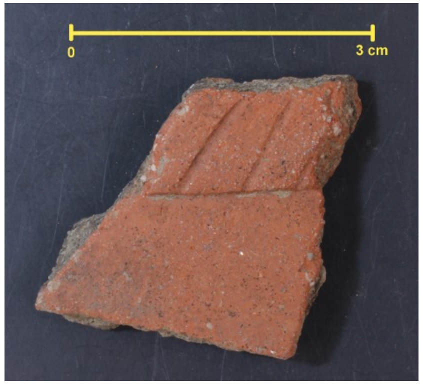	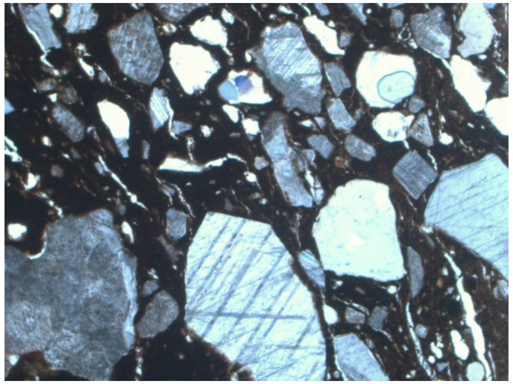	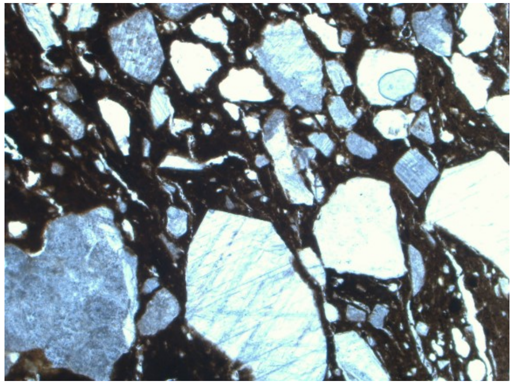
MB2.4	4	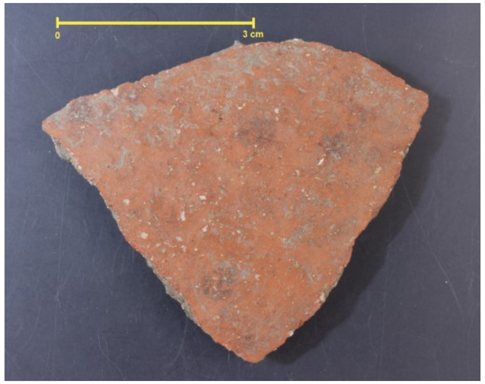	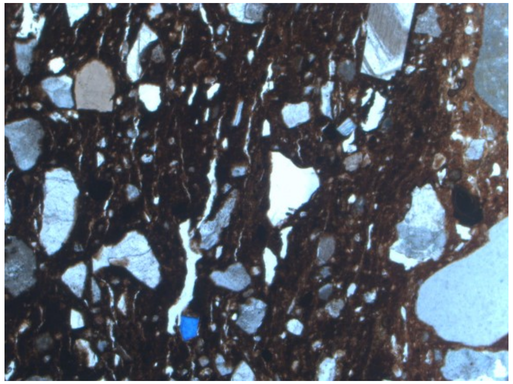	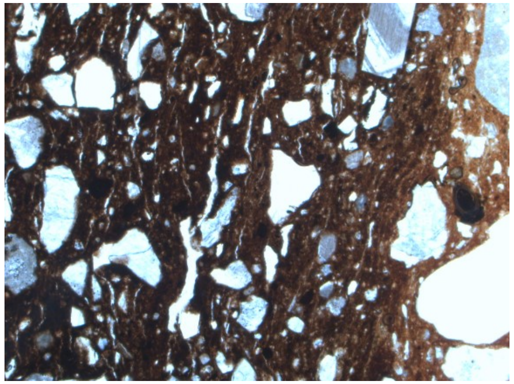
MB2.5	4	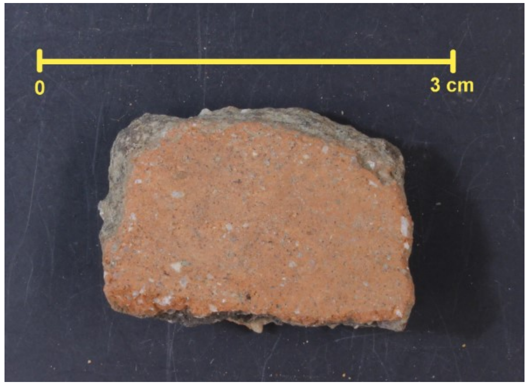	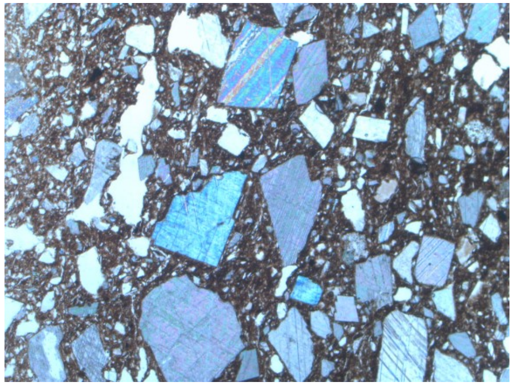	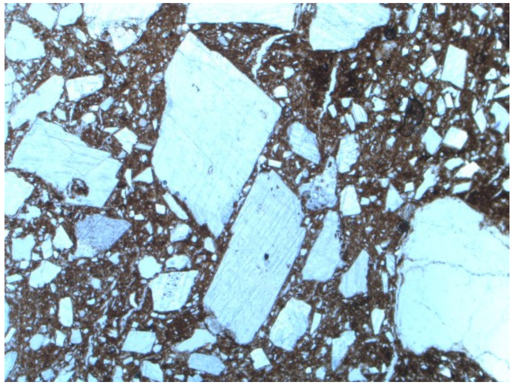

MB2.6	4	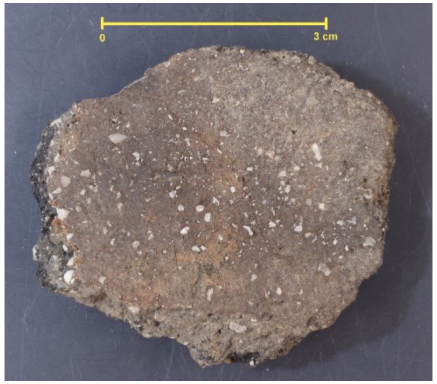	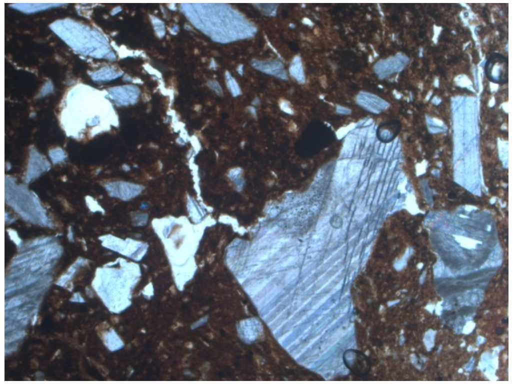	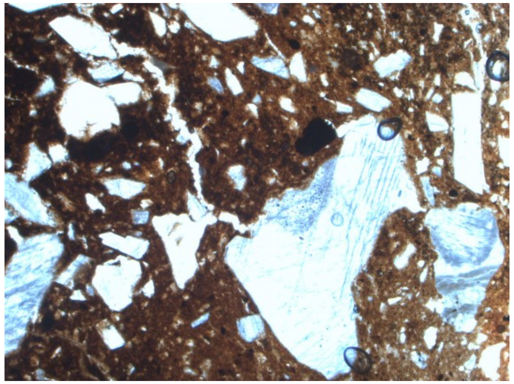
MB2.7	4	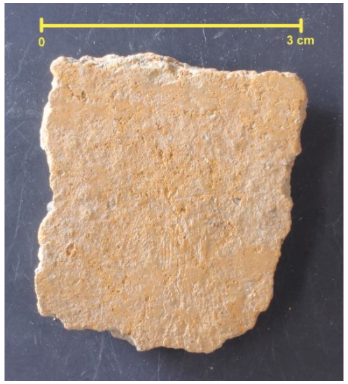	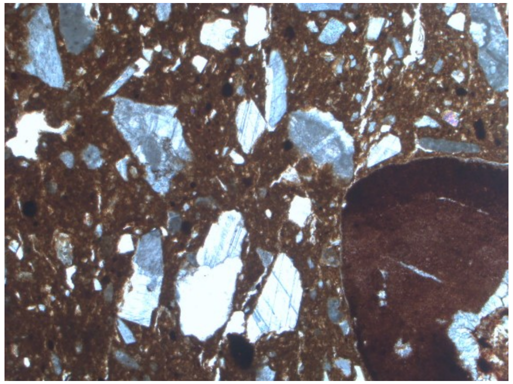	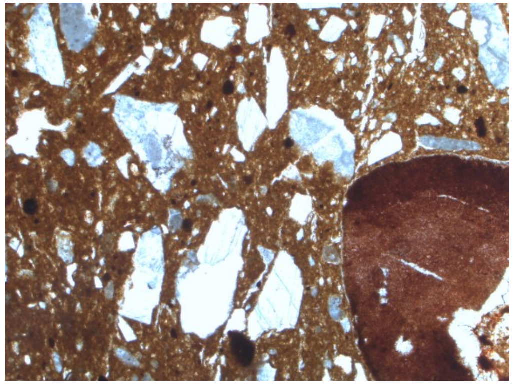
MB2.8	4	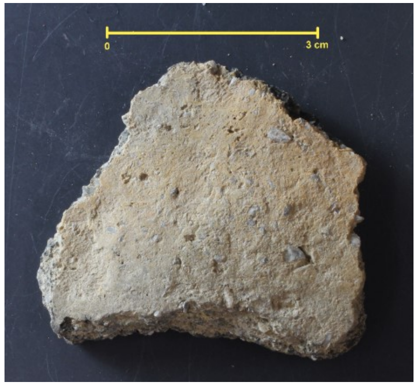	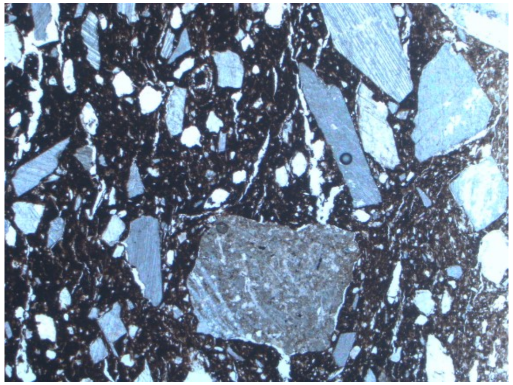	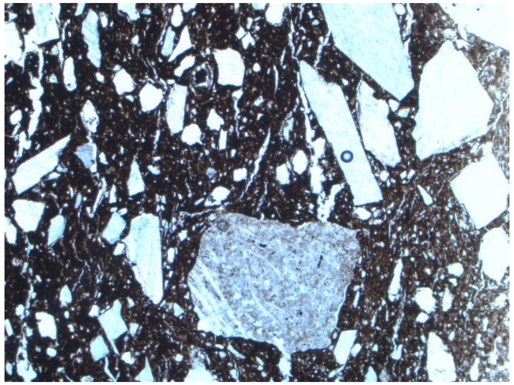

MB2.9	4	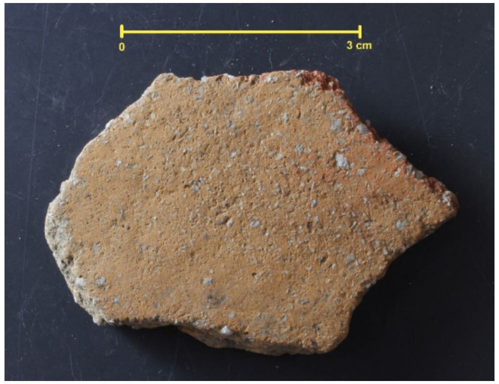	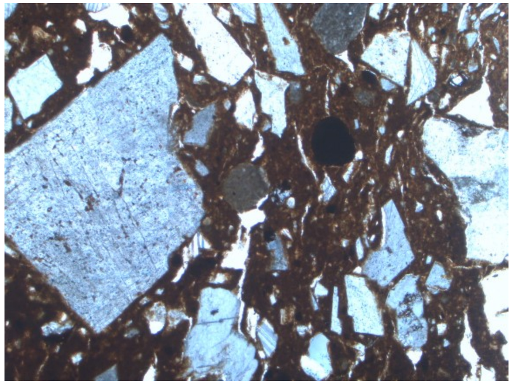	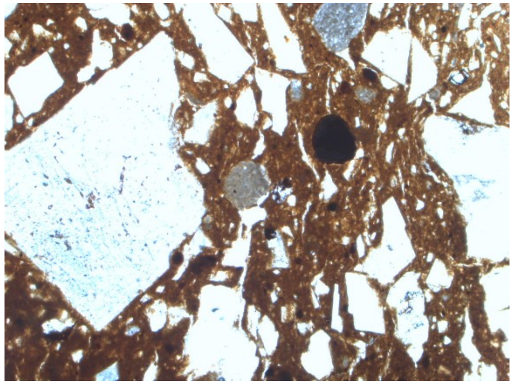
MB2.10	2.3	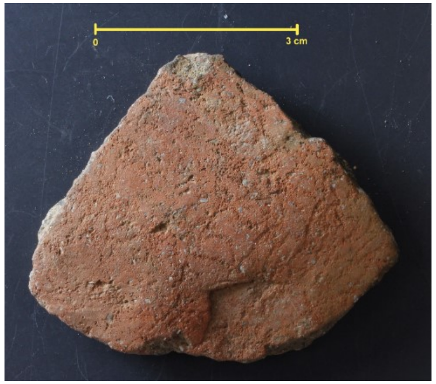	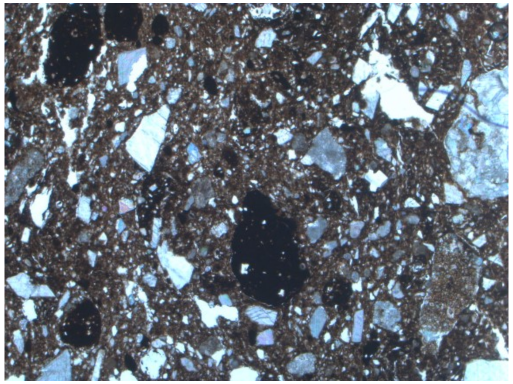	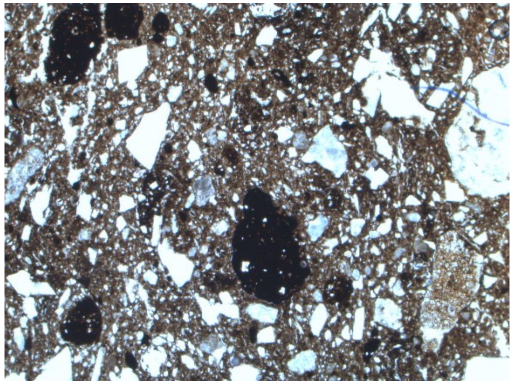
MB2.11	4	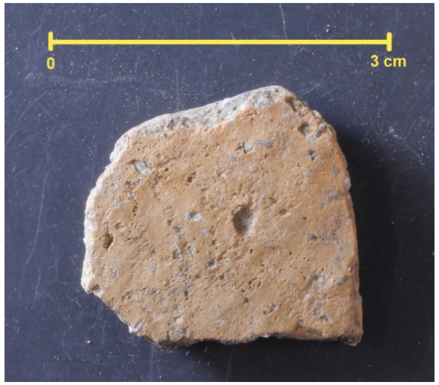	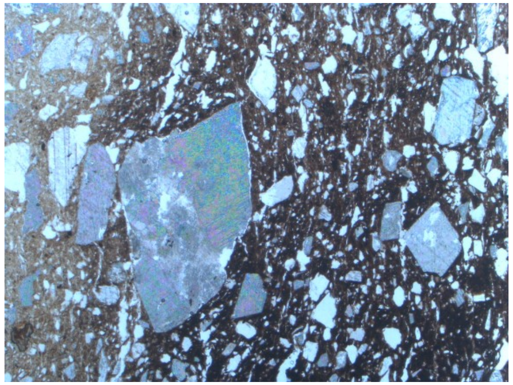	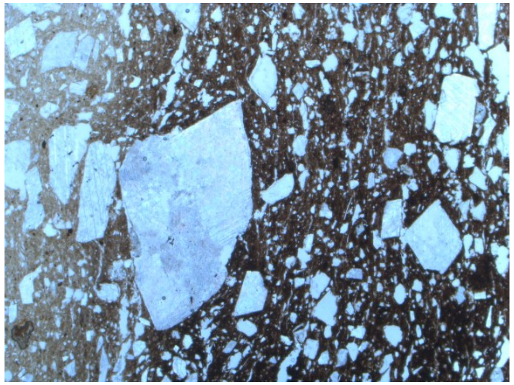

MB2.12	4	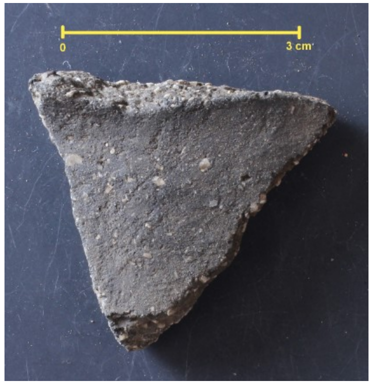	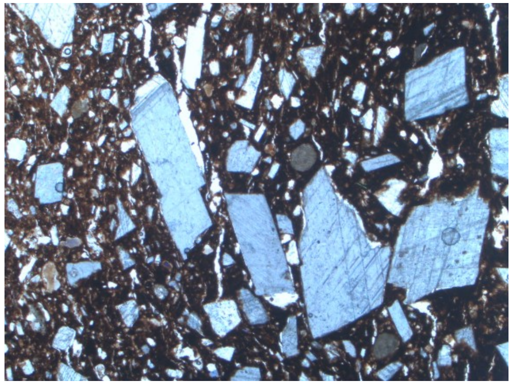	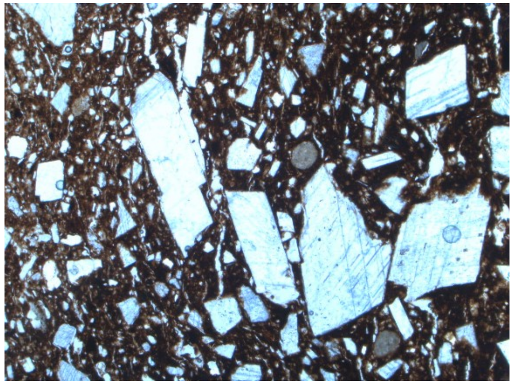
MB2.13	4	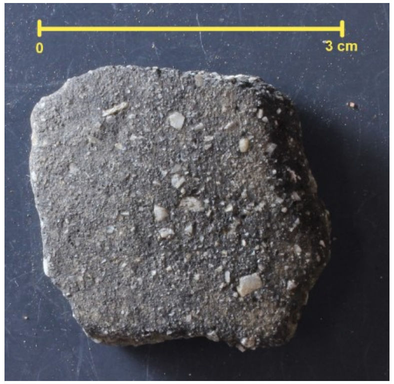	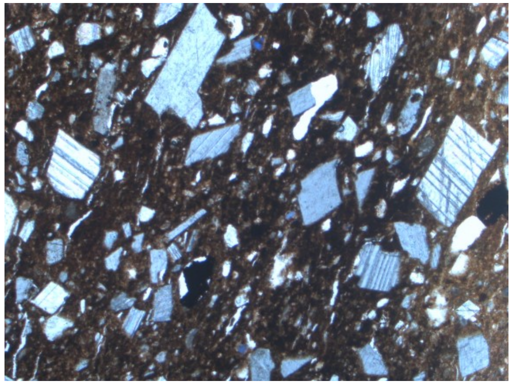	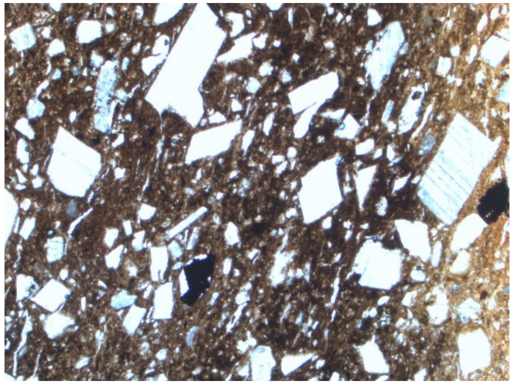
MB2.14	4	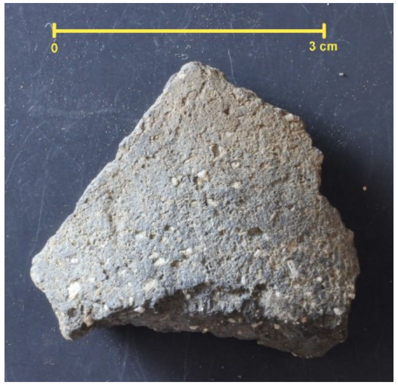	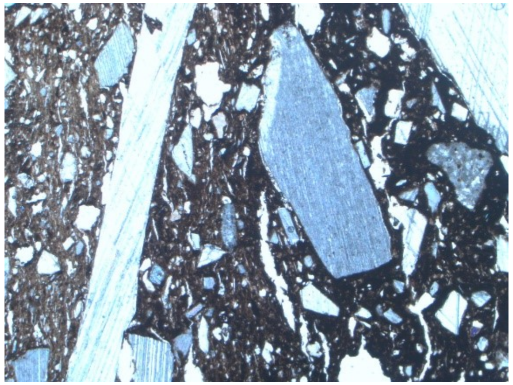	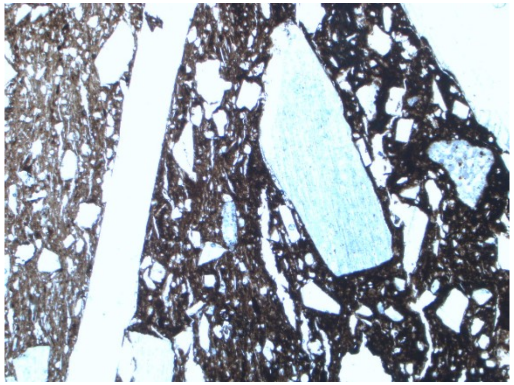

MB2.15	2.3	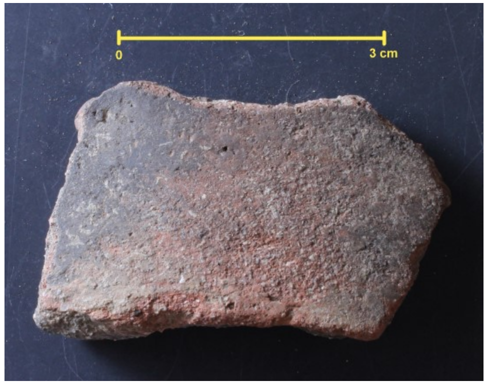	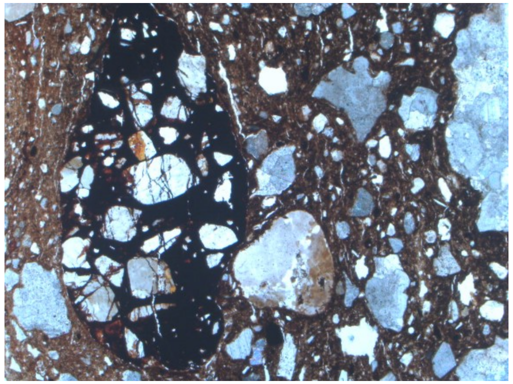	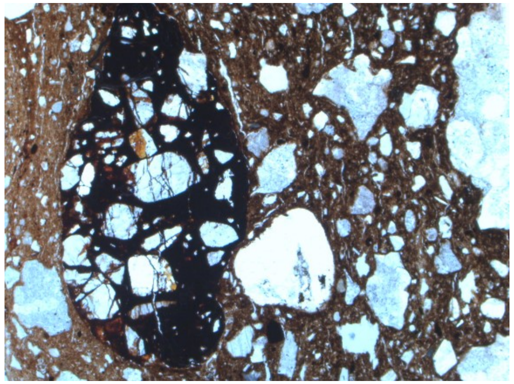
MB2.16	3.1	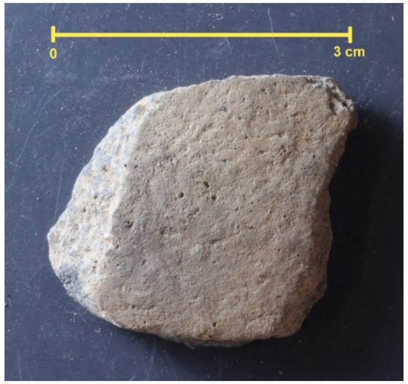	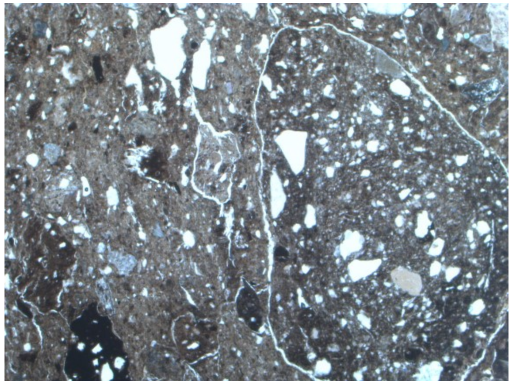	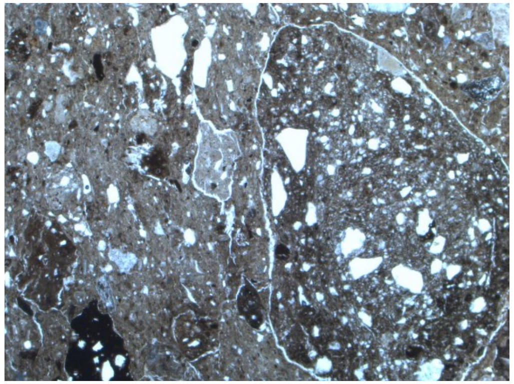
MB2.17	3.2	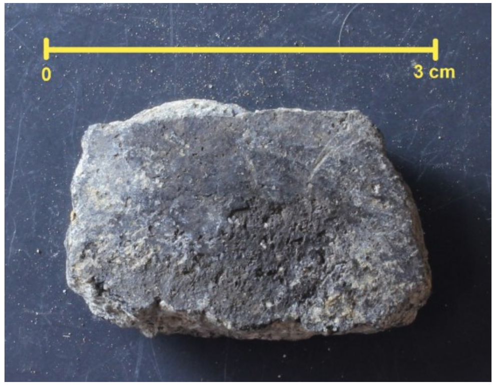	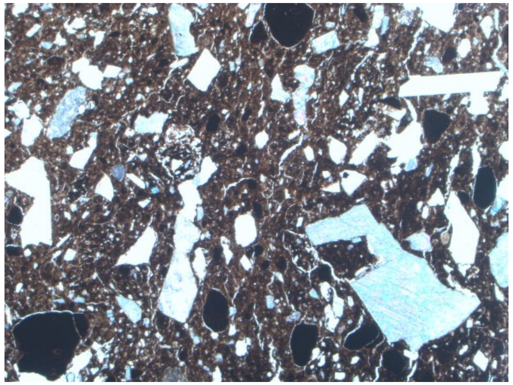	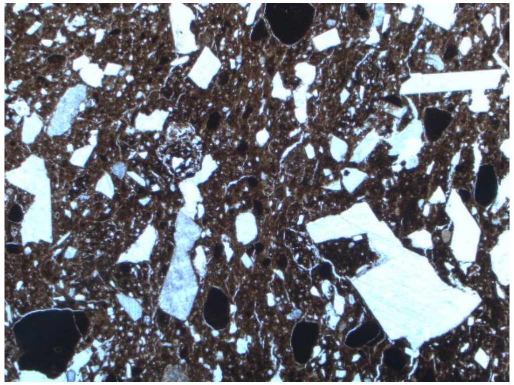

MB2.18	4	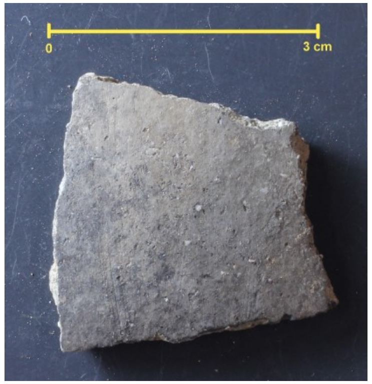	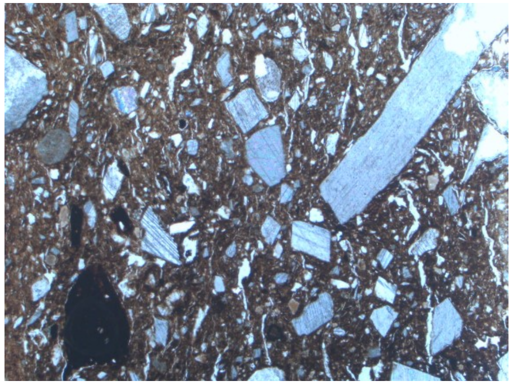	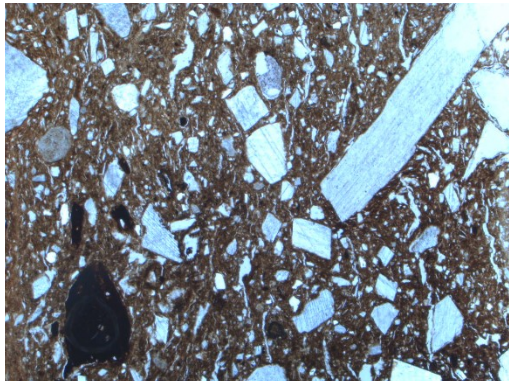
MB2.19	4	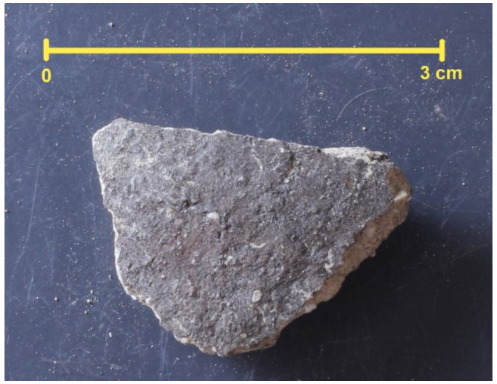	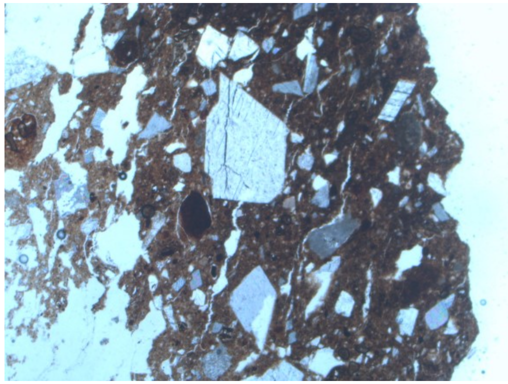	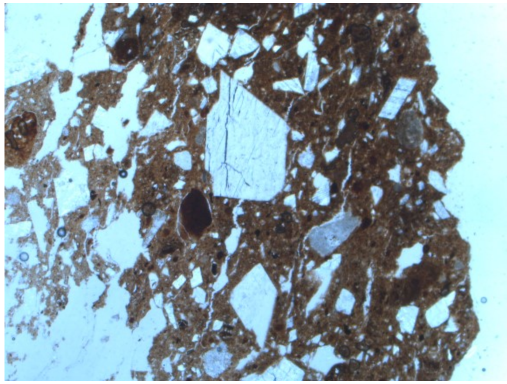
MB2.20	2.1	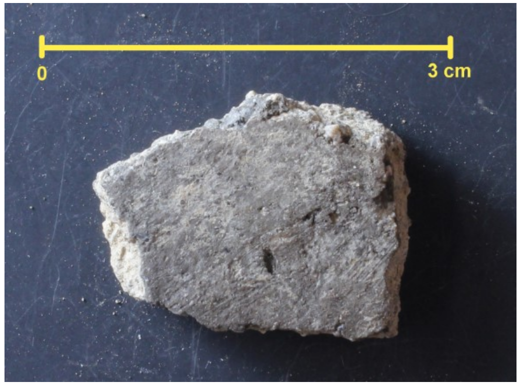	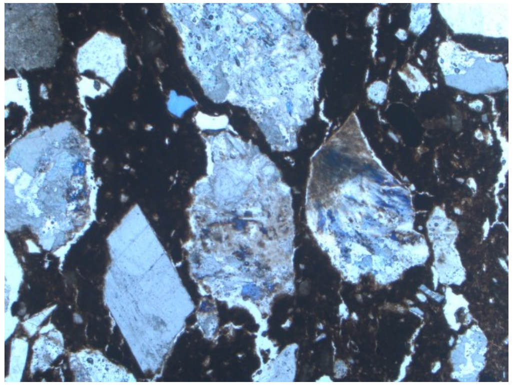	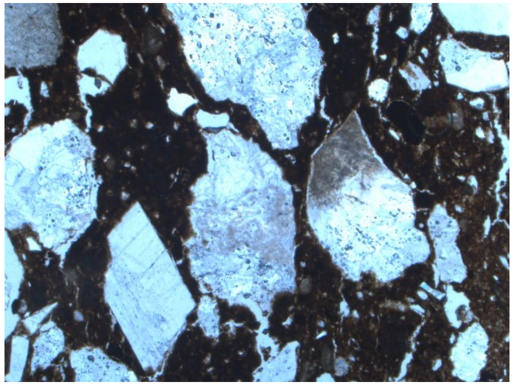

MB2.21	4	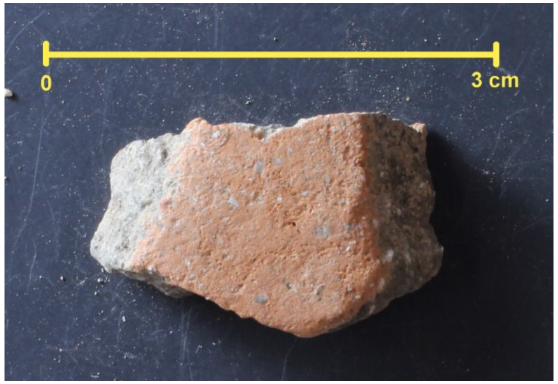	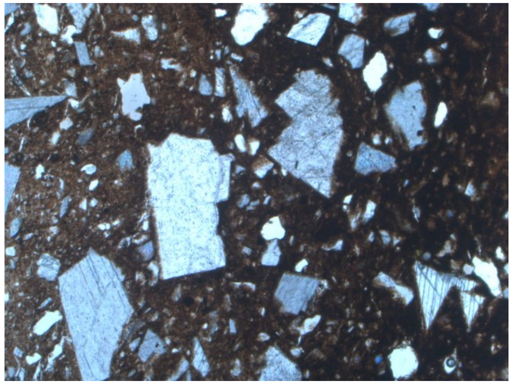	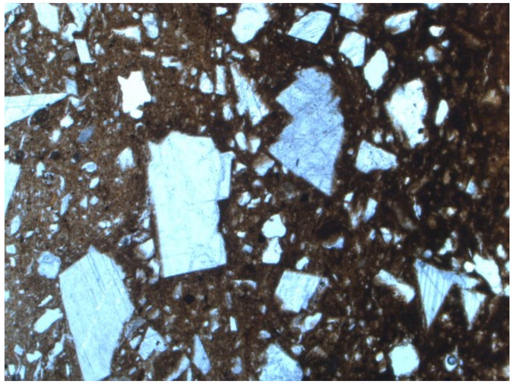
MB2.22	4	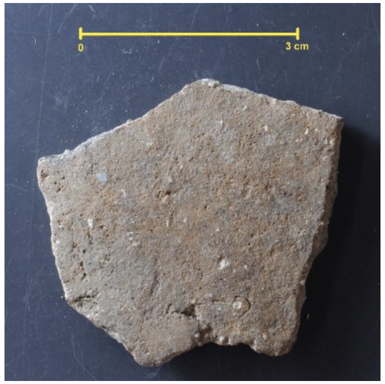	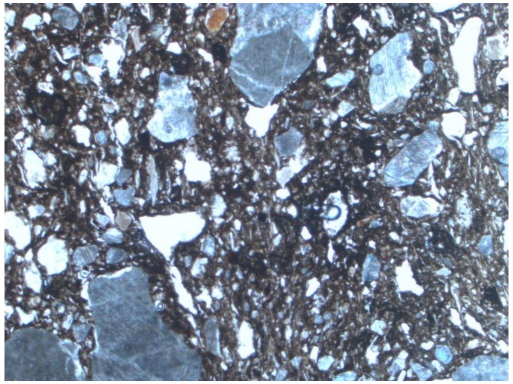	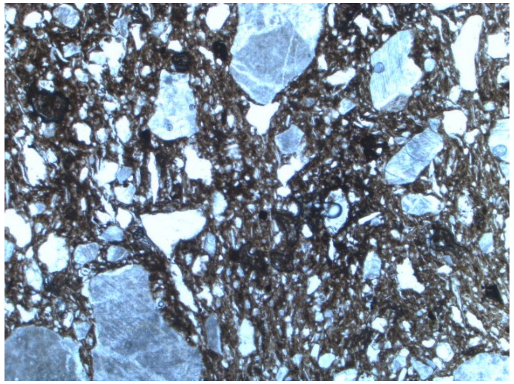
MB2.23	3.2	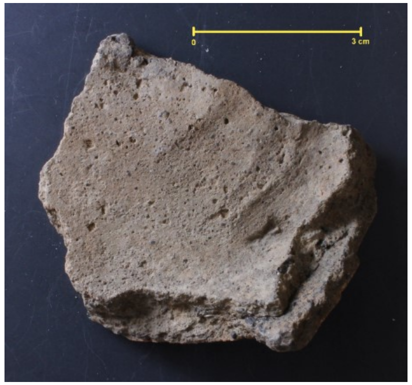	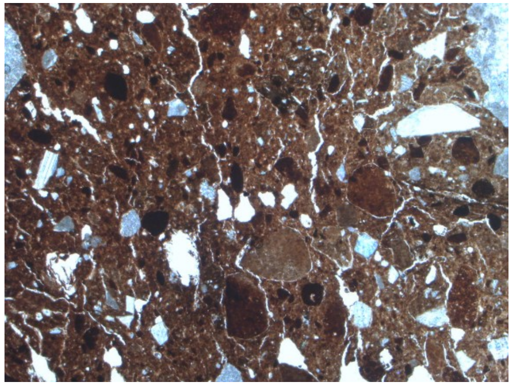	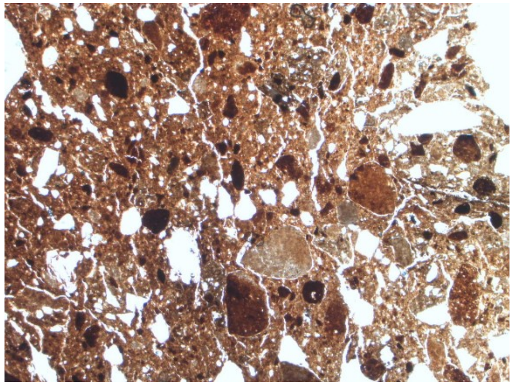

MB2.24	4	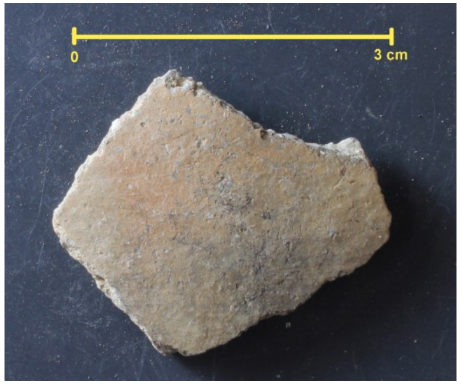	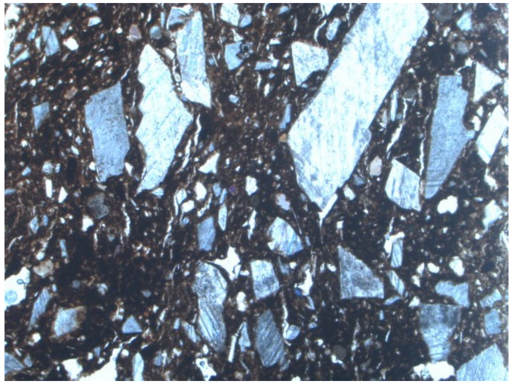	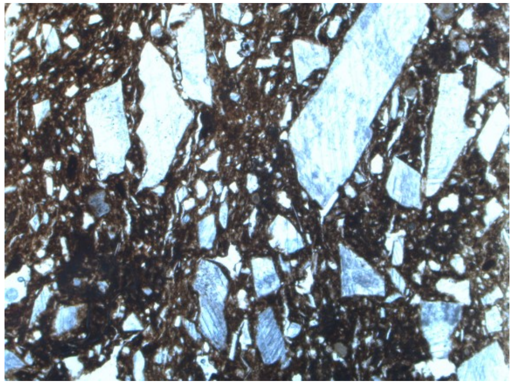
MB2.25	3.1	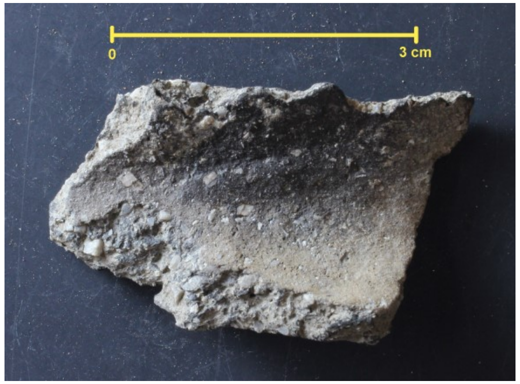	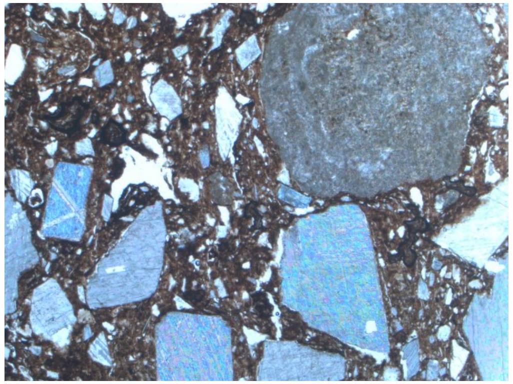	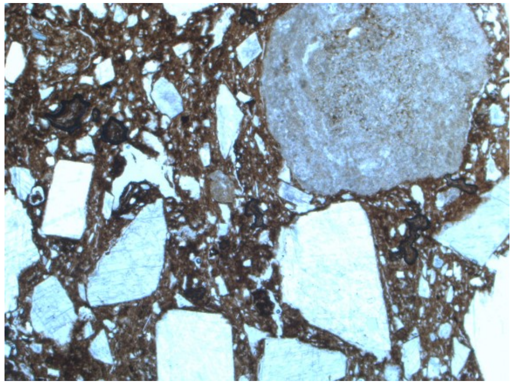
MB2.26	4	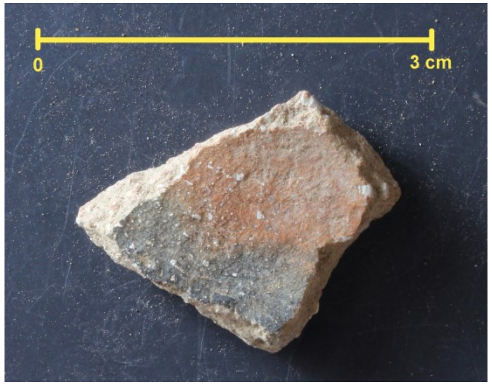	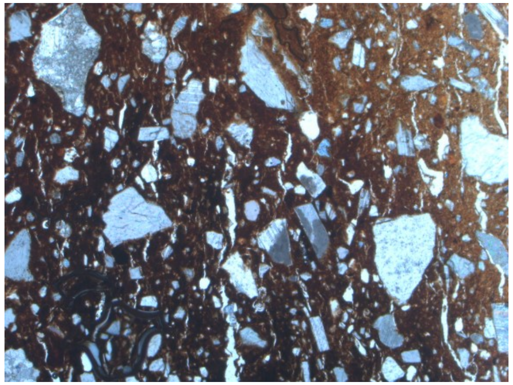	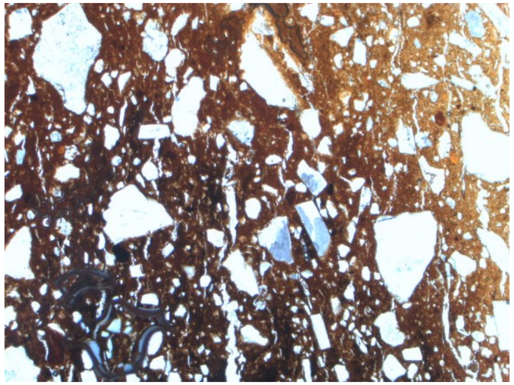

MB2.27	3.1	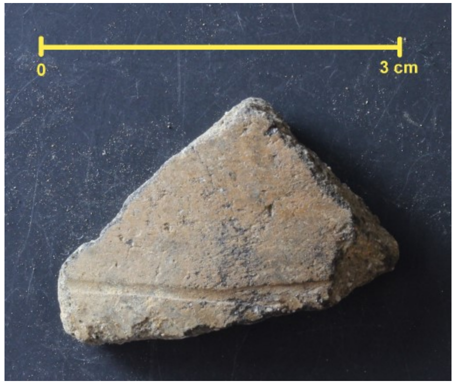	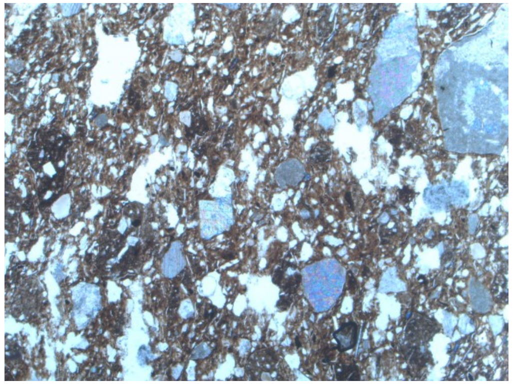	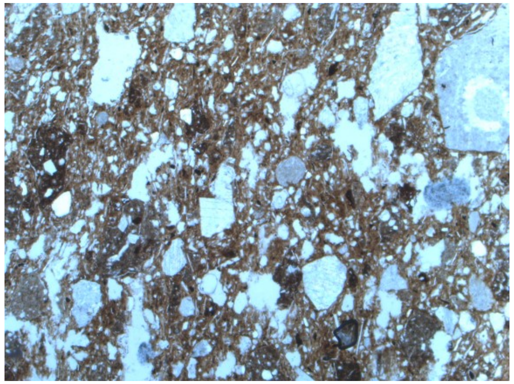
BMW1.<br>1		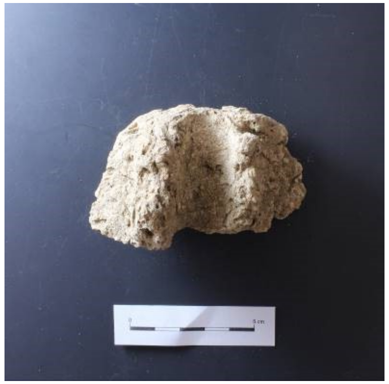	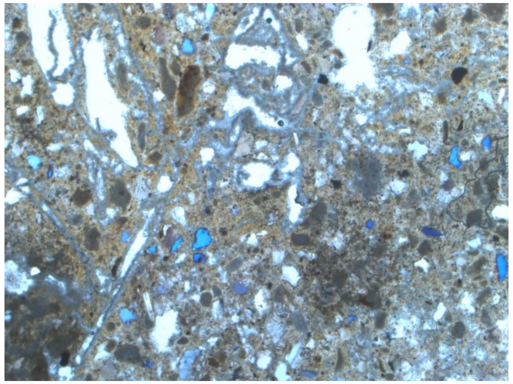	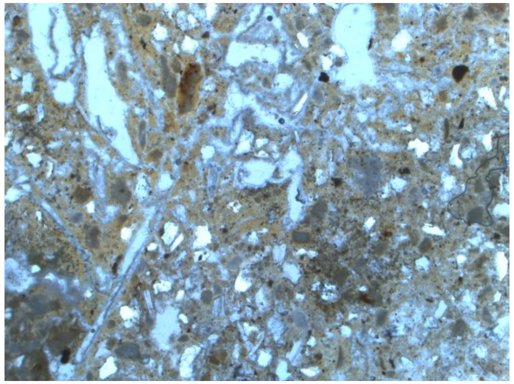
BMW1.<br>2		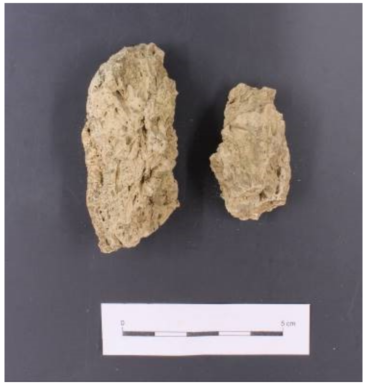	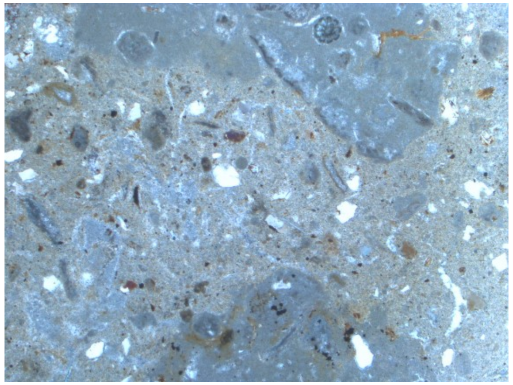	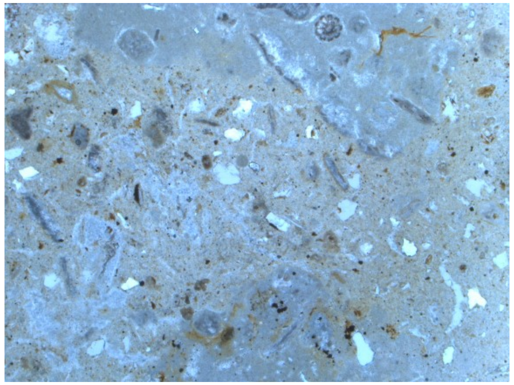

BMW1.3		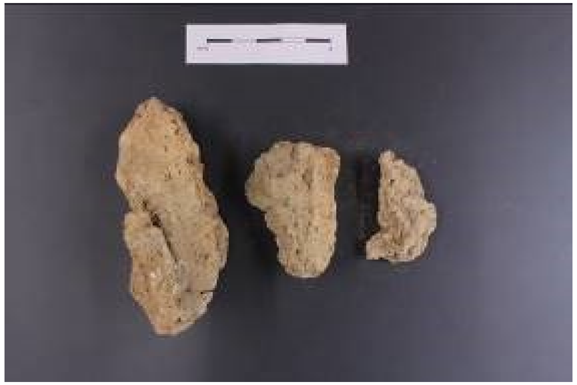	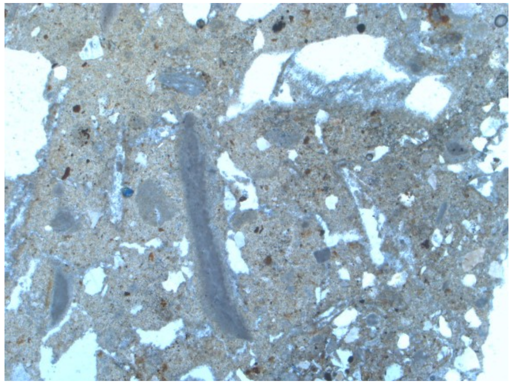	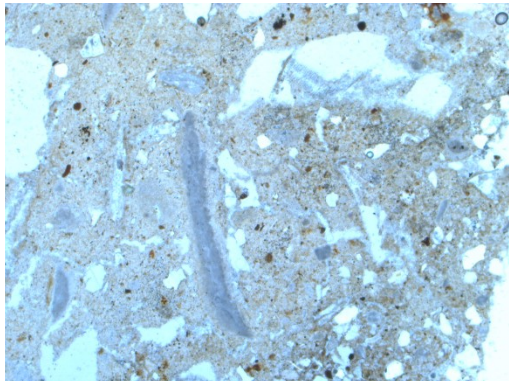

Wheel-made

1.1 Fine sand MB1.2, MB1.3, MB1.4, MB1.5, MB1.6, MB1.7, MB1.8, MB1.11, MB1.13, MB1.14, MB1.16, M1.19

Inclusions
3-5%. eq & el. va-wr. 0.75-0.01 mm. Open spaced. Poorly sorted. Weak alignment to the vessel margins. Unimodal grain size distribution.

Coarse fraction
60%. 0.75-0.1 mm

Predominant:	Quartz; el & eq. va-sr. 0.75-0.1mm, mode = 0.15mm. Some polycrystalline quartz fragments.
Common:	Feldspar: el & eq. va-sr. 0.75-0.1mm, mode = 0.15mm.
Common:	Micrite; el & eq. r-wr. 0.8-0.1mm, mode = 0.2mm.
Rare-Few:	TFs (in MB1.2, MB1.3, MB1.6, MB1.7, MB1.8); eq. sr-wr. 0.3-0.1mm, mode = 0.1mm. Discordant, red and black in XPL and PPL (x4) with sharp to clear boundaries and high optical density. Opaques and iron nodules.

Fine fraction
40%. 0.1-0.01 mm

Predominant:	Quartz
Common:	Micrite
Few:	Mica
Few:	Opaques

Matrix
94-96%. Moderately to non-calcareous (5-20%). Mid reddish- and orangey-brown to dark brown in XPL, light to mid orangey-brown and light to dark brown (MB1.11) in PPL (XPL x4). Optically inactive. Homogeneous. Silty iron and mica fragments in moderate alignment to the vessel margins. Flecks of sparry calcareous material visible at high magnification (40x), particularly in MB1.2, MB1.3, MB1.5, MB1.6, MB1.7, MB1.13, MB1.14. Abundant lathy mica, less so in MB1.5. Heterogeneous due to incomplete reduction in MB1.11 and MB1.16. SEM data confirmed the presence of zirconium in MB1.5, MB1.14 and rare earth elements Neodynium in MB1.16.

Voids
1%. Mega planar void in MB1.3 which is strongly aligned to the vessel margins. Meso vughs and vesicles in the other samples which are often aligned to the vessel margins, particularly in MB1.11.

Comments
This fabric is characterised by the presence of rare fine to coarse quartz in a moderately calcareous matrix. The fabric is very homogeneous and reddish in colour due to oxidised firing conditions. The paste might have been very well purified of non-plastic inclusions, probably through levigation. Other than the changes in wall-thickness in samples MB1.3 and MB1.5 there is little evidence for shaping methods utilised; the direction of the inclusions is largely random. In MB1.5 the alignment of quartz inclusions in the break might mark the edge of a coil which has been smoothed over but this is generally difficult to see. XRD analysis has shown no peak for calcite in sample 1.2.

The sintering of the matrix demonstrated by Fig S2.2 indicates firing temperatures were relatively high inside the kiln.

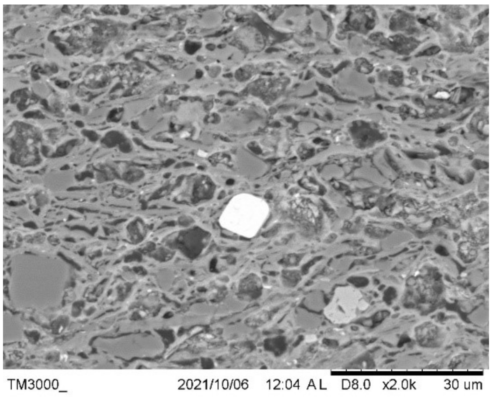

Fig S2.2 SEM image of MB1.14 showing sintering of matrix.

1.2 Moderately ferrous clay and fine sand MB1.1, MB1.9, MB1.15, MB1.17, MB1.18, MB1.20, MB1.21

Inclusions
5-15%. eq & el. va-wr. 0.8-0.01 mm. Open spaced. Poorly sorted. Weak alignment to the vessel margins. Unimodal grain size distribution.

Coarse fraction
40%. 0.75-0.1 mm

Predominant:	Quartz; el & eq. va-sr. 0.75-0.1mm, mode = 0.15mm. Some polycrystalline quartz fragments.
Common:	Feldspar; el & eq. va-sr. 0.75-0.1mm, mode = 0.15mm.
Few-common:	Micrite; el & eq. r-wr. 0.8-0.1mm, mode = 0.2mm. Particularly common in MB1.9 and MB1.15, with rounded well-sorted micrite and quartz.
Rare-Few:	TFs (in MB1.2, MB1.3, MB1.6, MB1.7, MB1.8); eq. sr-wr. 0.3-0.1mm, mode = 0.1mm. Discordant, red and black in XPL and PPL (x4) with sharp to clear boundaries and high optical density. Opaques and iron nodules.

Fine fraction
60%. 0.1-0.01 mm

Predominant:	Quartz
Few:	Micrite
Few:	Mica
Few:	Opaques

Matrix
80-94%. Non-calcareous. Mid reddish- and orangey-brown, dark-greyish brown in MB1.11 and MB1.16, multi-coloured in MB1.15, in XPL, light to mid orangey-brown in PPL (XPL x4). Optically active. Homogeneous. Silty iron and mica fragments in moderate alignment to the vessel margins. Heterogeneous due to incomplete incomplete oxidisation in MB1.15.

Voids
1-5%. Meso vughs and vesicles in the other samples which are often aligned to the vessel margins.

Comments
This fabric is characterised by the presence of fine to coarse quartz in a moderately ferrous matrix. Quartz is more abundant in fine fraction than in fabric 1.1. Optical activity of the matrix is common indicating that firing temperatures did not exceed 850°C. The abundance of calcareous material in the matrix is lower than in fabric 1.1 suggesting that, even though firing temperatures of fabrics 1.1 and 1.2 were the same, the former fabric might have lost optical activity sooner through the sintering of calcite in the matrix. Despite subtle variations between the matrices of fabrics 1.1 and 1.2 there are no clear diagnostic differences in the nature of the inclusions. It is thus possible that differences between the fabrics relate to natural variation in the clay source utilised.

1.3 Sand tempered fabric MB1.10, MB1.12

Inclusions
25%. eq & el. va-wr. 0.8-0.01 mm. Single to close-spaced. Well sorted. Weak alignment to the vessel margins. Bimodal grain size distribution.

Coarse fraction
40%. 0.75-0.1 mm

Predominant:	Quartz; el & eq. va-sr. 0.75-0.1mm, mode = 0.15mm. Some weathered, polycrystalline quartzite fragments that could be of metamorphic origin due to mildly granoblastic texture.
Common:	Feldspar; el & eq. va-sr. 0.75-0.1mm, mode = 0.15mm.

Common:	Micrite; el & eq. r-wr. 0.8-0.1mm, mode = 0.2mm. Composed of rounded micrite nodules.
Rare-Few:	TFs; eq. sr-wr. 0.3-0.1mm, mode = 0.1mm. Discordant, red and black in XPL and PPL (x4) with sharp to clear boundaries and high optical density. Opaques and iron nodules.

Fine fraction
60%. 0.08-0.01 mm
Predominant: Quartz
Few: Micrite
Few: Mica
Few: Opaques

Matrix
74%. Calcareous. Mid reddish-brown in XPL, light to mid orangey-brown in PPL (XPL x4). Optically active. Homogeneous. Silty iron and calcareous fragments in moderate alignment to the vessel margins.

Voids
1%. Mega planar void in MB1.3 which is strongly aligned to the vessel margins. Meso vughs and vesicles in the other samples which are often aligned to the vessel margins.

Comments
This fabric is characterised by the presence of abundant angular quartz and feldspar inclusions and a bimodal grain size distribution. The inclusions are well-sorted and are weakly aligned to the margins of the vessel. The fabric was probably tempered with sandy deposits of mixed origin. The matrix is optically active suggesting that firing temperatures were relatively low. Fine fraction is calcareous with flecks of sparry calcite visible at high magnification (40x).

2.1 **Crushed gypsum** MB2.20

Inclusions
50%. eq & el. va-sr. 2.5-0.02 mm. Single to close-spaced. Poorly sorted. Weak alignment to the vessel margins. Bimodal grain size distribution.

Coarse fraction
80%. 2.5-0.2 mm

Dominant:	Anhydrite/gypsum (calcite); eq & el. va-sa. 2.5-0.3mm, mode = 0.8mm. The altered calcite often has a radial pattern and feathery texture suggesting this has been formed by the evaporation of calcite. Anhydrite is found in calcareous deposits formed in sedimentary and evaporated calcite described as 'carniolas', which also includes dolomite. Carniolas can be found about 6km NW of Monte Bernorio.
Common:	Calcite; el & eq. va-a. 1.2-0.2mm, mode = 0.7mm.
Few:	Micrite; el & eq. r-wr. 2.4-0.5mm, mode = 0.5mm. Composed of rounded micrite nodules.
Few:	TFs; eq. sr-wr. 0.5-0.2mm, mode = 0.25mm. Discordant, dark-brown to black in XPL and PPL (x4) with sharp to clear boundaries and high optical density. Opaques and iron nodules.

Fine fraction
20%. 0.2-0.05 mm
Predominant: Calcite
Common: Micrite
Common: Opaques
Few: Feldspar
Rare: Lathy mica

Matrix

45%. Moderately calcareous. Mid brown in XPL, mid reddish-brown in PPL (XPL x4). Optically active. Homogeneous except for clay pellets and opaques.

Voids
5%. Macro channels which are strongly aligned to the vessel margins.

Comments
This fabric is characterised by the presence of coarse, angular fragments of anhydrite, which is a calcareous rock affected by evaporation, creating a feathery texture. This rock is recognised in sample MB2.20 by its fibrous texture that radiates from a centre. Other minerals have high relief calcite intergrowth and porphyroblasts. Together with sedimentary calcite and dolomite this type of rock is grouped under the name 'carniolas'. The bimodal grainsize distribution of the calcareous inclusions indicate tempering. Other than the nature of the temper this fabric is similar to fabric 4.

2.2 **Crushed metamorphic inclusions** MB2.3

Inclusions
40%. eq & el. va-wr. 2.5-0.05 mm. Single to close-spaced. Poorly sorted. Weak alignment to the vessel margins. Bimodal grain size distribution.

Coarse fraction
60%. 2.5-0.2 mm

Dominant:	Calcite; el & eq. a-sr. 2.5-0.2mm, mode = 1mm. Occasionally polycrystalline with irregular grain boundaries.
Common:	Dolomite: el & eq. a-sr. 2.5-0.2mm, mode = 1mm. Distinguished from calcite by rhombic shape and twinning along short axis of the rhombs.
Few:	Polycrystalline quartz; el & eq. sr-r. 2.0-0.8mm, mode = 1.0mm. Composed of polycrystalline feldspar and quartz. Polycrystalline inclusions have granoblastic texture with irregular grain boundaries between the quartz/feldspar minerals and undulate extinction of quartz, suggesting this could be metamorphic.
Few:	Feldspar; el & eq. sa-sr. 0.7-0.2mm, mode = 0.2 mm.
Few:	Quartz; el & eq. sa-sr. 0.7-0.2mm, mode = 0.2 mm.
Rare:	TFs; eq. sr-wr. 0.5-0.2mm, mode = 0.25mm. Discordant, dark-brown to black in XPL and PPL (x4) with sharp to clear boundaries and high optical density. Opaques and iron nodules.

Fine fraction
40%. 0.2-0.05 mm

Dominant:	Calcite
Common:	Opaques
Few:	Feldspar
Few:	Quartz
Rare:	Lathy mica

Matrix
55%. Moderately calcareous. Light to dark brown in XPL, mid reddish-brown to dark brown in PPL (XPL x4). Optically active. Homogeneous except for opaques.

Voids
5%. Macro channels which are strongly aligned to the vessel margins.

Comments
This fabric is characterised by the presence of coarse polycrystalline calcite. Polycrystalline fragments composed of quartz and feldspar could be part of a metamorphic rock as polycrystalline quartz has a granoblastic texture. There are no metamorphic rock outcrops nearby so it is possible sample MB2.3 is an

import. The matrix has common opaques suggesting that the clay was iron-rich. Polycrystalline calcite might have been added as temper to the moderately calcareous clay as suggested by the bimodal grain size distribution and crisp condition of the metamorphic inclusions. MB2.3 is decorated with incisions and has an oxidised surface. This could thus reflect a fine ware sherd.

2.3 **Calcareous clay with sparry limestone inclusions** MB2.10

Inclusions
40%. eq & el. a-wr. 2.25-0.05 mm. Single to close-spaced. Poorly sorted. Weak alignment to the vessel margins. Unimodal grain size distribution.

Coarse fraction
40%. 2.25-0.2 mm

Dominant:	Calcite; el & eq. a-r. 1.6-0.2mm, mode = 0.7mm. Sparry calcite with high interference colours. Also rhombic calcite with 120° twinning.
Common:	Micrite; el & eq. sr-wr. 1.1-0.2mm, mode = 0.7mm.
Few:	TFs; eq. sr-wr. 1.25-0.2mm, mode = 0.3mm. Discordant, dark-brown to black in XPL and PPL (x4) with sharp to clear boundaries and high optical density.
Rare:	Quartz; el & eq. sa-sr. 0.3-0.2mm, mode = 0.2 mm.

Fine fraction
60%. 0.5-0.05 mm

Dominant:	Calcite
Common:	Micrite
Few:	Opaques and iron nodules
Few:	Quartz

Matrix
57%. Calcareous. Light to dark brown in PPL and XPL (x4). Moderate optical activity. Homogeneous.

Voids
3%. Macro channels which are strongly aligned to the vessel margins.

Comments
This fabric is characterised by the presence of sparry calcite inclusions in a calcareous matrix. It is possible that calcite was added as temper to calcareous clay. The roundness of the inclusions suggests a sandy temper was used instead of crushed calcite. The fabric distinguishes itself from fabric 4 through the use of calcareous clay.

3.1 **Clay pellets/grog** MB2.16, MB2.25, MB2.27

Inclusions
40%. eq & el. va-wr. 2.25-0.05 mm. Single to close-spaced. Poorly sorted. Weak alignment to the vessel margins. Moderate bimodal grain size distribution.

Coarse fraction
30%. 2.25-0.2 mm

Common-Dominant:	TFs; eq. a-wr. 2.25-0.25mm, mode = 0.8mm. Discordant to concordant, black, dark-brown and light-brown in XPL and PPL (x4) with sharp to merging boundaries and low, neutral and high optical density. Clay pellets. Some have internal banding, particularly in MB2.16, in which clay pellets are dominant. Inclusions are quartzose or calcareous. TFs with calcareous inclusions are grog, as suggested by the shape of the TF. Inclusions in these fragments are oriented in alignment with the margin of the parent vessel in MB2.25.

Common: Calcite; el & eq. a-r. 1.75-0.2mm, mode = 0.5mm. Includes individual and polycrystalline calcite.

Common: Micrite; el & eq. sr-wr. 1.1-0.2mm, mode = 0.7mm.

Rare: Quartz; el & eq. sa-sr. 0.3-0.2mm, mode = 0.2 mm.

Fine fraction
70%. 0.5-0.05 mm
Dominant: Calcite
Frequent: Quartz
Common: Micrite
Few: Opaques and iron nodules
Rare (MB2.16) - common: Mica

Matrix
57%. Moderately calcareous. Light greyish-brown and mid orangey-brown in PPL and XPL (x4). Optically active. Heterogeneous due to TFs.

Voids
3%. Macro channels and meso vesicles which are moderately aligned to the vessel margins. Some voids contain charred organic material.

Comments
This fabric is characterised by the presence of TFs which might be grog or a mixture of clay pellets and grog. The TFs are heterogeneous with different sizes and degrees of angularity. In sample MB2.16 variation is observable in the inclusions in the TFs, which either contain predominantly quartz or calcareous inclusions. Some of the TFs with calcareous inclusions have a shape that is indicative of grog, with one straight side which could be the margin of a crushed pottery vessel.

The calcareous matrices of these vessels contain frequent quartz suggesting that different clays were used than in most of the other samples in the Monte Bernorio group. The addition of grog is another indication that this fabric represents a different recipe.

Relic organic inclusions in some of the voids might have entered the clay matrix during mixing of the clay and temper, as suggested by their low frequency. The XRF readings of MB2.16 and MB2.27 shows that these samples are geochemically more similar to the wheel-made pottery fabrics, which might be due to the lower frequency of calcareous inclusions in this fabric, which contains relatively more quartz.

The chemical signature of MB2.25 is very different from the other samples in this fabric and fits better with the samples of fabric 4. However, because grog temper was found in this specimen which also includes quartz and rare mica in fine fraction the sample was grouped in this fabric group.

3.2 **Clay pellets/grog and calcareous inclusions** MB2.17, MB2.23

Inclusions
30%. eq & el. va-wr. 1.5-0.05 mm. Single to close-spaced. Poorly sorted. Weak alignment to the vessel margins. Unimodal grain size distribution.

Coarse fraction
50%. 1.5-0.2 mm

Common-Dominant: TFs; eq. a-wr. 2.25-0.2mm, mode = 0.6mm. Discordant to concordant, dark-brown mid reddish-brown and mid greyish-brown in XPL and PPL (x4) with sharp to merging boundaries and neutral and high optical density. Well-rounded clay pellets of homogeneous iron-rich plastic material. Mid greyish-brown ARFs or grog with fine quartzose inclusions. A dark-brown grog inclusion in MB2.23 has straight boundary on one side and poorly sorted calcareous inclusions and TFs (>0.13mm).

Common: Calcite; el & eq. va-sr. 1.2-0.2mm, mode = 0.5mm.

Common: Micrite; el & eq. sr-wr. 1.0-0.2mm, mode = 0.6mm.

Fine fraction
50%. 0.2-0.05 mm
Dominant: Mid to dark-brown clay pellets
Frequent: Calcite
Common: Micrite

Matrix
67%. Moderately calcareous. Mid reddish-brown and mid brown in PPL and XPL (x4). Optically active. Heterogeneous due to TFs.

Voids
3%. Macro and meso channels and meso vesicles which are moderately aligned to the vessel margins. Some voids contain charred organic material.

Comments
This fabric is characterised by the presence of frequent well-rounded iron rich clay pellets, occasional grog fragments and angular calcite inclusions. Some of the TFs with calcareous inclusions have a shape that is indicative of grog, with one straight side which could be the margin of a crushed pottery vessel. This is particularly clear in MB2.23.

The fabric differs from 3.1 because iron-rich clay pellets are more common suggesting that different clay was used. The grain size of this inclusions in fabric 2.3 is more strongly unimodal suggesting that the size of added grog is smaller. Nevertheless, grog tempering is distinctive and therefore these different fabrics should be considered as part of a similar technological tradition. The chemical composition of these samples indicates that these samples contain more calcareous and more ferrous material than the samples of fabric 3.1.

4 Calcite tempered fabric MB2.1, MB2.5, MB2.6, MB2.7, MB2.8, MB2.9, MB2.11, MB2.12, MB2.13, MB2.14, MB2.18, MB2.21, MB2.22, MB2.24, MB2.2, MB2.4, MB2.15, MB2.19, MB2.26

Inclusions
30-40%. eq & el. va-wr. 3.5-0.05 mm. Single to close-spaced. Poorly sorted. Weak alignment to the vessel margins. Bimodal grain size distribution.

Coarse fraction
60%. 3.5-0.2 mm

Predominant: Calcite; el & eq. va-sr. 3.0-0.2mm, mode = 0.8mm. Predominantly coarse, angular crystalline calcite. Occasionally polycrystalline. Rounded and sparitic in MB2.22. Weathering also visible in MB2.6. Some long, thin laths.

Common: Micrite; el & eq. sr-wr. 3.5-0.2mm, mode = 0.8mm.

Rare-common: Quartz; el & eq. a-sr. 1.5-0.2mm, mode = 0.4mm. Occasionally polycrystalline. Undulate extinction pattern in MB2.5. Polycrystalline in MB2.7 and MB2.9 are silica-rich inclusions which might be of quartzite, strongly weathered and replaced by crystalline and sparitic calcite. Some inclusions of K-feldspar replaced by sparite.

Rare: TFs; eq. a-wr. 2.5-0.2mm, mode = 0.5mm. Discordant, dark-brown mid reddish-brown and black in XPL and PPL (x4) with sharp to clear boundaries and neutral to high optical density. Well-rounded clay pellets of homogeneous iron-rich plastic material. Fine quartz inclusions in clay pellet in MB2.5. Opaques with rounded feldspar inclusions (0.4-0.02mm, mode = 0.2) in MB2.15.

Few: Feldspar; eq. sa-r. 1.0-0.1mm, mode = 0.5mm. Anhedral to euhedral feldspar altered by sericite. In MB2.2, MB2.4 and MB2.15.

Very rare; Grog (MB2.26); a. 0.4 mm. Concordant with sharp boundaries and neutral optical density. Fine rounded and angular lathy and equant inclusions of possible mica and calcite.

Fine fraction
40%. 0.2-0.05 mm
Dominant: Calcite

Few-Common:	Quartz
Few:	Mica
Few:	Clay pellets and opaques
Rare:	Pyroxene (hornblende?)

Matrix
57-67%. Calcareous in MB2.5, moderately to non-calcareous in other samples. Light to mid orange-brown and dark-brown in XPL and PPL (x4). Optically active. Homogeneous and heterogeneous (in MB2.2, MB2.4, MB2.15, MB2.19, MB2.26).

Voids
3%. Macro and meso channels in moderate alignment to the vessel margins.

Comments
This fabric is characterised by the presence of coarse angular calcite inclusions, occasional micrite and clay pellets in a moderately to non-calcareous matrix with sub-angular to sub-rounded quartz. The bimodal grain size distribution of the inclusions, angularity and freshness of the calcite suggest that calcite was crushed and added as temper. The low prevalence of calcareous material in the matrix suggests also suggests that calcite was added instead of naturally present in the clay matrix. Exceptions to this statement are MB2.5 which has a higher prevalence of calcite in fine fraction and the matrix and MB2.22, which has more rounded, weathered calcareous inclusions that might be naturally present or added as sand temper.

The fabric is the most dominant in the hand-made group from Monte Bernorio. The shape of the calcite inclusions is often rhomboidal suggesting that dolomite was collected and crushed to produce temper. Dolomite is present at the site itself pointing to a possible local origin of tempering material. Clay, which is unlikely to be found on the top of the oppidum could be gathered in the valley where clay deposits are widespread. It is likely that clays were purified of larger inclusions prior to tempering since no diagnostic coarse inclusions are found in the matrix other than (polycrystalline) quartz and clay pellets.

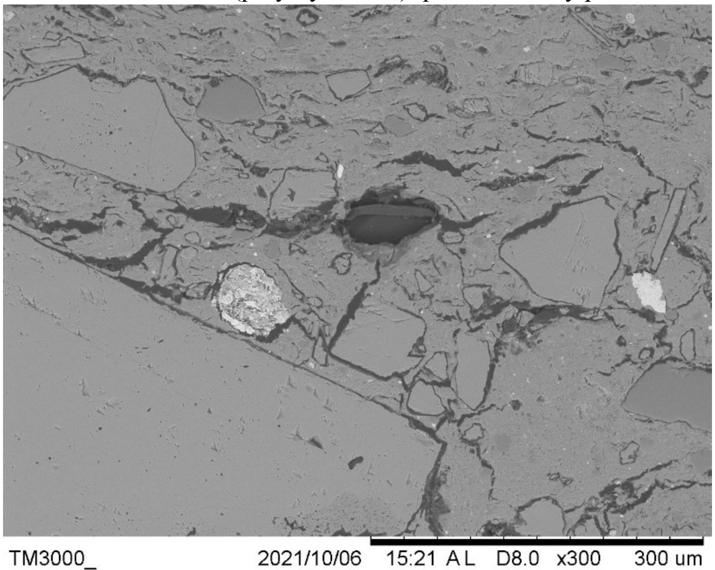

Fig S2.4 SEM image of MB2.26.

Streaking is visible in MB2.2, MB2.4, MB2.15, MB2.19, MB2.26, resembling the heterogeneous matrix of geological sample 5 (Keuper deposit). This fabric is heterogeneous with variation in the frequencies of different minerals in each of the samples. Particularly MB2.26 has more abundant calcite which is generally angular with smaller grain size in coarse fraction (0.6-02mm). MB2.26 has calcite with iron oxide banding while MB2.15 has more abundant silica-rich TFs with undulate extinction and granoblastic texture. MB2.7 and MB2.9 have a relatively high ferrous component in the matrix of these clays, which is also suggested by the XRF readings.

GEOLOGICAL SAMPLES

Terra rossa from site MG1

Inclusions
30%. eq & el. va-wr. 0.5-0.02 mm. Single to close-spaced. Moderately sorted. Unimodal grainsize distribution.

Coarse fraction
60%. 0.5-0.02 mm

Predominant: Calcite; el & eq. va-r. 0.5-0.02mm, mode = 0.3mm. Predominantly angular weathered calcite. High relief calcite crystals are strongly fractured and bright whitish in colour.

Common: Opaques (iron nodules); el & eq. r-wr. 1.5-0.02mm, mode = 0.6mm. Sometimes iron TFs with calcite inclusions.

Fine fraction
40%. 0.2-0.05 mm
Dominant: Calcite
Common: Opaques and iron nodules

Matrix
70%. Calcareous. Mid orange-brown in XPL and PPL (x4). Homogeneous.

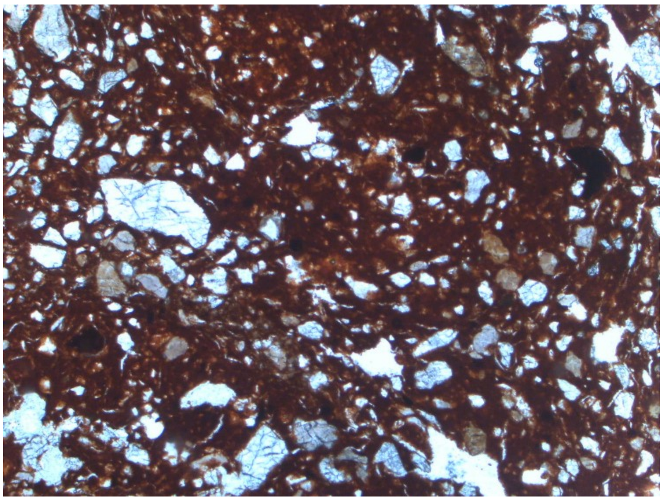

Fig S2.5 Microphotograph of MG1 (XPL), field of view = 3.0mm.

Comments
Strongly calcareous and moderately sorted clay. Firing at high temperature (900°C) may have caused cracking and fracturing of the calcite inclusions. The clay sample contains calcite in much greater abundance than the wheel-made samples and calcite is different in sorting and size than the calcite in the hand-made samples. This is not a likely source clay for the ceramics samples described above, predominantly due to the difference in fine fraction, which in the case of hand-made ceramics is more strongly bimodal.

Keuper deposit Aguilar del Campoo MG5

Inclusions
5%. eq & el. va-wr. 2.5-0.125 mm. Single to close-spaced. Moderately sorted. Unimodal grainsize distribution.

Coarse fraction
60%. 3.5-0.2 mm

Predominant: TFs; el & eq. wr. 2.5-0.4mm, mode = 1.5mm. Heterogeneous rounded calcareous clay pellets. Some contain fine texture with light yellowish or greyish-brown colours and occasional grains of subhedral feldspar. Other TFs are streaks of greyish clay.

Common: Feldspar; el & eq. sr-r. 1.5-0.125mm, mode = 1mm. Anhedral and subhedral feldspars, which have been altered by sericite.

Common: Quartz: el & eq. sr-r. 1.5-0.125mm, mode = 1mm

Fine fraction
40%. 0.125-0.05 mm
Dominant: Feldspar
Common: Quartz
Common: Iron nodules
Rare: Mica needles

Matrix

67%. Moderately-calcareous. Light orange-brown, reddish and grey in XPL and PPL (x4). Heterogeneous due to variation in oxidisation of Cuyper deposit. Grey streaking visible clearly as well as rounded grey nodules in fine fraction.

Comments

Moderately calcareous, poorly sorted clay. Obtained from Keuper deposit next to limestone intrusions. In this deposit we can expect calcareous inclusions, quartz and evaporites which are not represented in the sample. Instead, the sample contains fine quartz and feldspar which also occurs in the ceramic samples. It is therefore a likely source are for clays used at Monte Bernorio, both in the production of hand-made and wheel-made pottery.

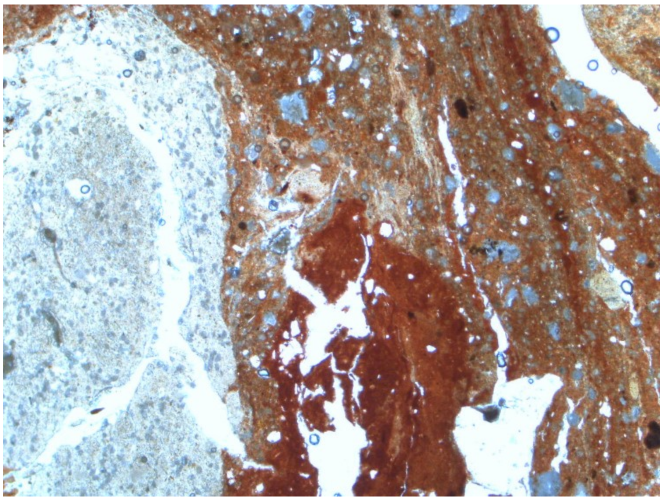

Fig S2.6 Microphotograph of MG5 (XPL), field of view = 3.0mm.
