# Supplementary material for: Capturing technological crossovers between clay crafts: An archaeometric perspective on the emergence of workshop production in Late Iron Age northern Spain
Source: PLoS One. 2023 May 5;18(5):e0283343. doi: 10.1371/journal.pone.0283343 (PMC10162541; doi:10.1371/journal.pone.0283343)
Supplement: S3 File — (PDF) [file pone.0283343.s003.pdf]

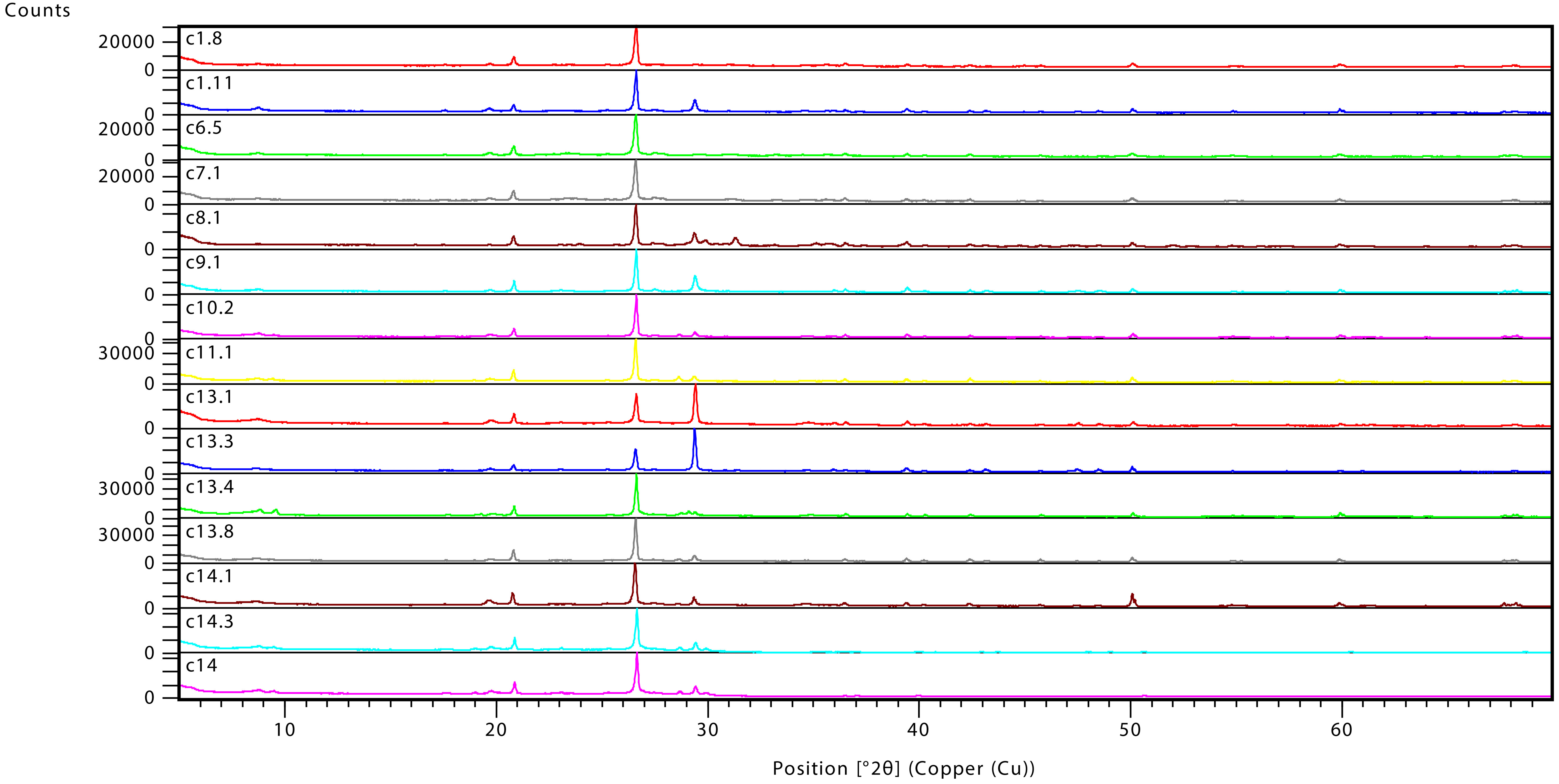

Counts
c1.8
c1.11
c6.5
c7.1
c8.1
c9.1
c10.2
c11.1
c13.1
c13.3
c13.4
c13.8
c14.1
c14.3
c14
20000
30000
0
10
20
30
40
50
60
Position [°2θ] (Copper (Cu))

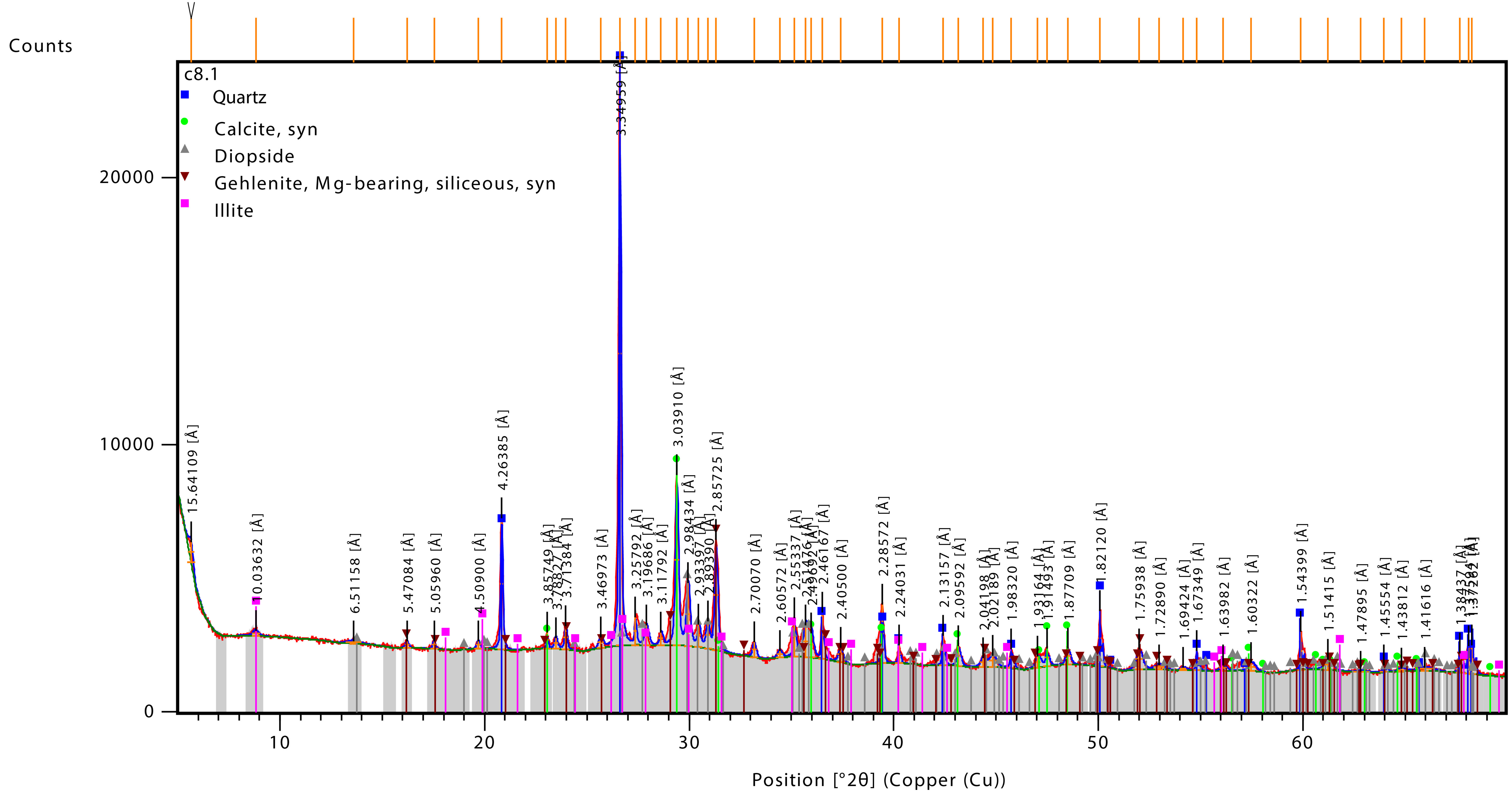

Counts
c8.1
Quartz
Calcite, syn
Diopside
Gehlenite, Mg-bearing, siliceous, syn
Illite
20000
10000
0
10
20
30
40
50
60
Position [°2θ] (Copper (Cu))
15.64109 [Å]
10.03632 [Å]
6.51158 [Å]
5.47084 [Å]
5.05960 [Å]
4.50900 [Å]
4.26385 [Å]
3.34959 [Å]
3.46973 [Å]
3.03910 [Å]
2.85725 [Å]
2.70070 [Å]
2.46167 [Å]
2.40500 [Å]
2.28572 [Å]
2.24031 [Å]
2.13157 [Å]
2.09592 [Å]
1.98320 [Å]
1.87709 [Å]
1.82120 [Å]
1.75938 [Å]
1.72890 [Å]
1.67349 [Å]
1.63982 [Å]
1.60322 [Å]
1.54399 [Å]
1.51415 [Å]
1.47895 [Å]
1.45554 [Å]
1.43812 [Å]
1.41616 [Å]

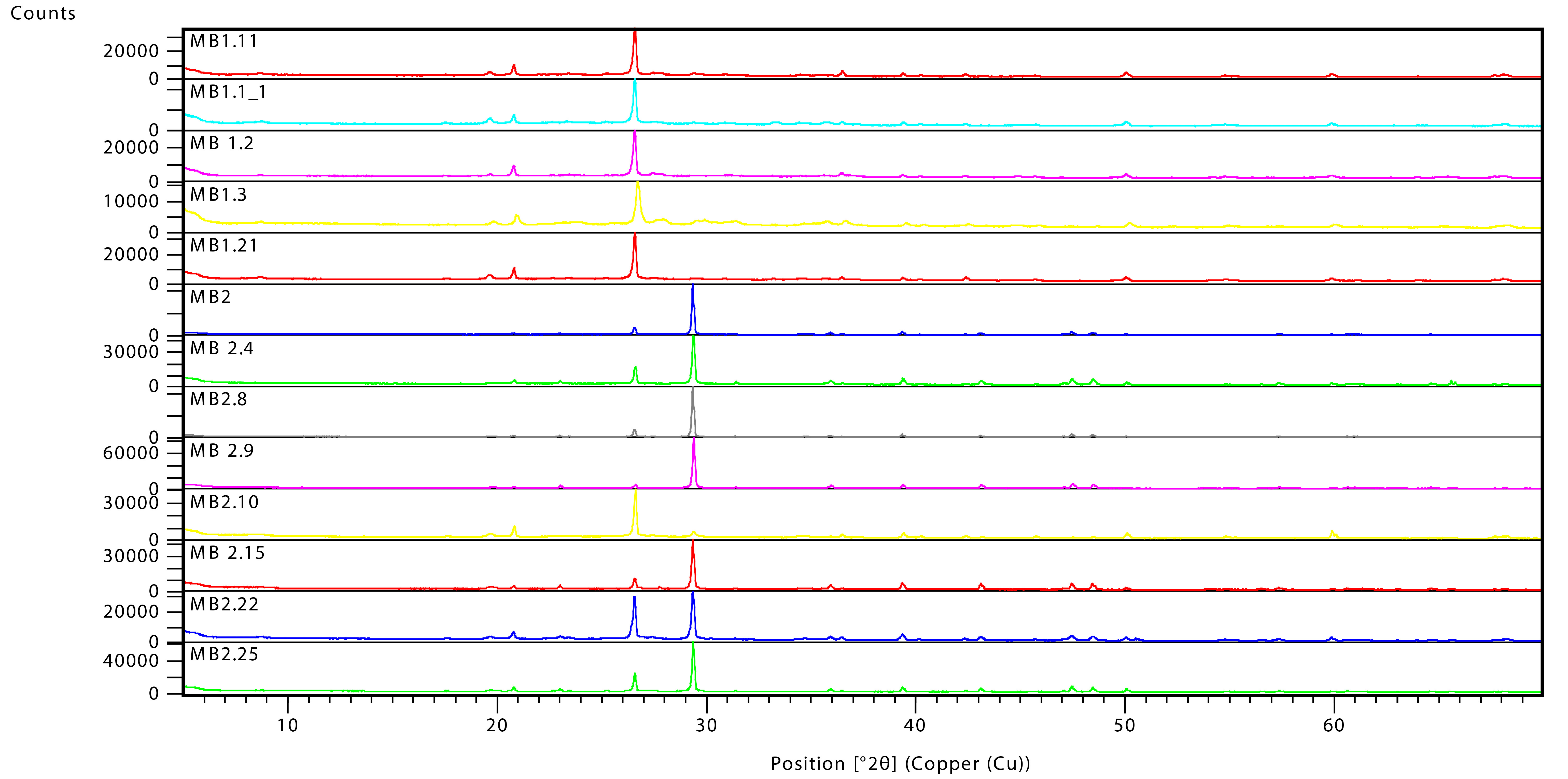
Counts
MB1.11
MB1.1_1
MB 1.2
MB1.3
MB1.21
MB2
MB 2.4
MB2.8
MB 2.9
MB2.10
MB 2.15
MB2.22
MB2.25
20000
10000
30000
60000
40000
0
10
20
30
40
50
60
Position [°2θ] (Copper (Cu))

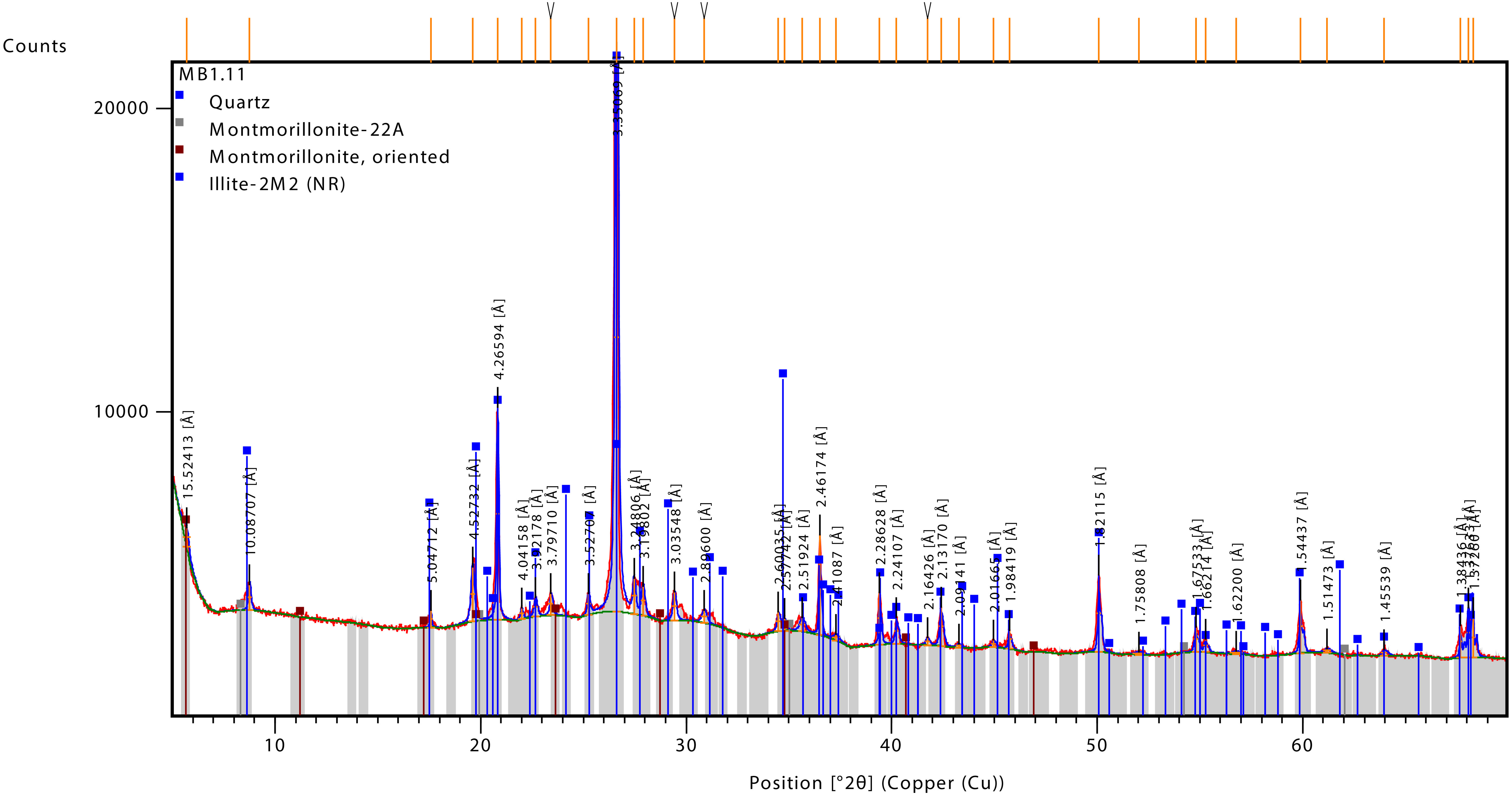
Counts
MB1.11
Quartz
Montmorillonite-22A
Montmorillonite, oriented
Illite-2M2 (NR)
20000
10000
15.52413 [Å]
10.08707 [Å]
5.04712 [Å]
4.52732 [Å]
4.26594 [Å]
4.04158 [Å]
3.79710 [Å]
3.03548 [Å]
2.89600 [Å]
2.51924 [Å]
2.46174 [Å]
2.28628 [Å]
2.24107 [Å]
2.16426 [Å]
2.13170 [Å]
1.98419 [Å]
1.75808 [Å]
1.62200 [Å]
1.54437 [Å]
1.51473 [Å]
1.45539 [Å]
10
20
30
40
50
60
Position [°2θ] (Copper (Cu))

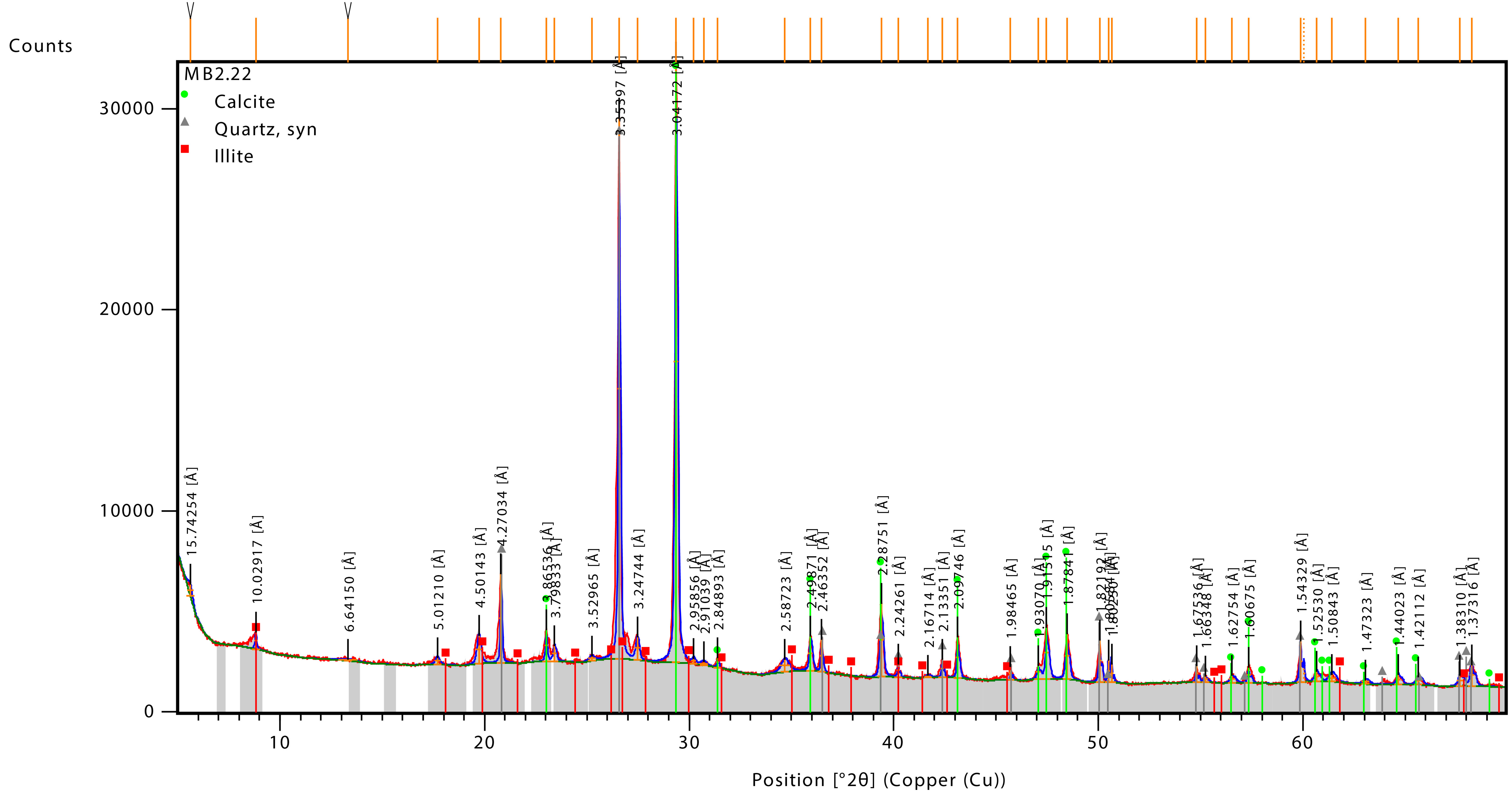
Counts
MB2.22
Calcite
Quartz, syn
Illite
30000
20000
10000
0
10
20
30
40
50
60
Position [°2θ] (Copper (Cu))
15.74254 [Å]
10.02917 [Å]
6.64150 [Å]
5.01210 [Å]
4.50143 [Å]
4.27034 [Å]
3.79833 [Å]
3.52965 [Å]
3.35397 [Å]
3.24744 [Å]
3.04172 [Å]
2.95856 [Å]
2.91039 [Å]
2.84893 [Å]
2.58723 [Å]
2.49871 [Å]
2.46352 [Å]
2.28751 [Å]
2.24261 [Å]
2.16714 [Å]
2.13351 [Å]
2.09746 [Å]
1.98465 [Å]
1.93070 [Å]
1.87841 [Å]
1.82192 [Å]
1.67536 [Å]
1.66348 [Å]
1.62754 [Å]
1.60675 [Å]
1.54329 [Å]
1.52530 [Å]
1.50843 [Å]
1.47323 [Å]
1.44023 [Å]
1.42112 [Å]
1.38310 [Å]
1.37316 [Å]
